# Real-world feasibility of co-administration of RSV, COVID-19, and influenza vaccines in older adults: a VAERS-based analysis

**DOI:** 10.3389/fphar.2025.1682119

**Published:** 2025-10-20

**Authors:** Zhen Wei, Shilun Yu

**Affiliations:** ^1^ Department of Respiratory and Critical Care Medicine, Tengzhou Central People’s Hospital, Tengzhou, Shandong, China; ^2^ School of Acupuncture and Tuina, Shandong University of Traditional Chinese Medicine, Jinan, Shandong, China

**Keywords:** RSV and COVID-19 co-administration group, RSV and seasonal influenza co-administration group, triple vaccination group, VAERS, adverse events following immunization

## Abstract

**Background:**

Respiratory syncytial virus (RSV), COVID-19, and seasonal influenza represent significant health threats to older adults. These pathogens frequently co-circulate during the same seasons. Co-administration of vaccines targeting these viruses is a practical strategy to enhance vaccination coverage and convenience. However, real-world safety data on the simultaneous administration of RSV, COVID-19, and influenza vaccines in adults aged 60 and older remain limited.

**Objective:**

This study aimed to explore the safety profile of co-administration of RSV, COVID-19, and influenza vaccines in adults aged 60 and older. Using data from the U.S. Vaccine Adverse Event Reporting System (VAERS), the study aimed to identify patterns of adverse events following immunization (AEFIs) and generate hypotheses regarding possible safety signals, to guide clinical practice and public health strategies. These findings should be interpreted as hypothesis-generating and require confirmation through robust observational studies.

**Methods:**

AEFI reports for individuals aged 60 years or older were retrieved from VAERS between 3 May 2023, and 1 January 2025. Three study cohorts were established: the RSV–COVID-19 co-administration group, the RSV–seasonal influenza co-administration group, and the triple-vaccination group. Four disproportionality analysis methods were applied—reporting odds ratio (ROR), proportional reporting ratio (PRR), Bayesian Confidence Propagation Neural Network (BCPNN), and Multi-item Gamma Poisson Shrinker (MGPS)—to detect potential safety signals.

**Results:**

A total of 479, 576, and 194 AEFI reports were gathered from the RSV–COVID-19, RSV–influenza, and triple-vaccination groups, respectively. Female reporters outnumbered male reporters by a factor of 2–3. More than 96% of AEFIs occurred within 30 days post-vaccination, with considerable variation in the onset time distribution. Non-serious AEFIs accounted for 85.6%–88.5%, with the majority of clinical outcomes classified as *recovered*. At the System Organ Class (SOC) level, AEFIs were primarily concentrated in general disorders and administration site conditions, as well as nervous system disorders and abnormal laboratory findings. Potential safety signals included ear and labyrinth disorders, skin and subcutaneous tissue disorders, and injury, poisoning, and procedural complications. At the Preferred Term (PT) level, commonly reported AEFIs included headache, fatigue, and injection site pain, while potential safety signals included aphasia, oral mucosal blisters, and gait disturbance. Among serious reports, high-confidence signals such as influenza-like illness, cerebrovascular accidents, and muscular weakness were identified across all groups. Some high-confidence signals, including sleep disorders and contusions, were also observed in non-serious reports.

**Conclusion:**

The overall safety profile of co-administered RSV, COVID-19, and influenza vaccines in older adults is favorable, with most AEFIs being expected and self-limiting. It is crucial to emphasize that this study is hypothesis-generating; the findings do not prove any causal association between vaccines and the reported adverse events. The potential signals of rare but high-risk events, such as neurological, psychiatric, and cardiovascular complications, must be investigated and confirmed in robust observational studies. Enhanced long-term surveillance is recommended to further understand these potential risks.

## 1 Introduction

Respiratory syncytial virus (RSV) is a significant pathogen that causes acute respiratory illness, affecting the upper and lower respiratory tracts (World Health Organization, 2025). While mild cases often resemble the common cold symptoms, severe infections can progress to critical conditions, including respiratory failure (Peña-López et al., 2024). Complications range from bacterial pneumonia and exacerbation of cardiopulmonary diseases to severe outcomes requiring mechanical ventilation or resulting in death (Woodruff et al., 2024; Yu et al., 2024). The severity of RSV infection is especially pronounced in older adults, with an estimated 123,000 to 193,000 RSV-related hospitalizations annually in the United States. Among individuals aged 60 years and older, approximately 24,400 to 34,900 require ICU admission, and RSV-related deaths range from 4,680 to 8,620 each year (Havers et al., 2024). This underscores the significant burden RSV places on older adults. In May 2023, the U.S. Food and Drug Administration (FDA) approved an RSV vaccine for individuals aged 60 and older. This vaccine has demonstrated efficacy in reducing RSV-related hospitalizations and emergency department visits, providing a critical tool to mitigate RSV-related disease burden in older adults (Payne et al., 2024).

In addition to RSV, older adults are also at heightened risk for both SARS-CoV-2 and seasonal influenza. These viruses frequently co-circulate during the same seasons, exacerbating their combined impact on public health (Goswami et al., 2025). Given the overlapping seasonal circulation of these viruses, the co-administration of vaccines targeting RSV, COVID-19, and seasonal influenza offers a practical and efficient strategy to reduce the overall disease burden in this vulnerable population. Co-administration of vaccines not only increases vaccination coverage but also alleviates the strain on healthcare systems, minimizes discomfort for individuals, and improves compliance by reducing the need for multiple separate vaccinations (Focosi, 2023). Furthermore, by addressing these pathogens simultaneously, combined vaccination helps establish a broad immune barrier quickly, ultimately reducing hospitalization and mortality rates (Bonanni et al., 2023). While this combined strategy is promising, its real-world safety profile requires robust evaluation.

Past studies have demonstrated the safety of individual RSV, COVID-19, and seasonal influenza vaccinations. However, real-world evidence regarding the safety of co-administration remains limited. Existing clinical trials have compared the co-administration of the RSV vaccine with COVID-19 or seasonal influenza vaccines to separate administration using randomized, controlled, double-blind methods (Dacso, 2025; Goswami et al., 2025). The results indicate that co-administration is non-inferior to separate administration in terms of immunogenicity and short-term safety, providing preliminary support for its real-world application (Dacso, 2025; Goswami et al., 2025). However, the practical application of co-administration still faces multiple limitations. Firstly, the clinical evidence supporting its widespread use, especially studies on the co-administration of the new RSV vaccine, remains limited (Maggi et al., 2025). Existing studies often suffer from insufficient sample sizes, underscoring the urgent need for large-scale, real-world adverse event monitoring data (Debbag et al., 2025; Maggi et al., 2025). Furthermore, the actual rate of co-administration remains well below expectations. Data shows that, even with policy support, the co-administration rate of COVID-19 and influenza vaccines remains low (Tzenios et al., 2022). This suggests that long-term safety, potential immune interference effects, and the occurrence of infrequent adverse events remain key concern for the public (Debbag et al., 2025). To gain a comprehensive understanding of the safety of co-administration, it is imperative to conduct broader real-world data studies.

As a national monitoring system jointly managed by the U.S. Centers for Disease Control and Prevention (CDC) and the U.S. Food and Drug Administration (FDA), the Vaccine Adverse Event Reporting System (VAERS) plays a crucial role in collecting post-vaccination adverse events and supporting post-market safety evaluations of vaccines. It has become an essential platform for vaccine safety monitoring (Shimabukuro et al., 2015). Drawing on the VAERS database, a series of studies have been conducted with in-depth analyses. For instance, the CDC’s Immunization Safety Office used VAERS data to evaluate the safety of RSV vaccination during pregnancy and found that preterm birth was the most commonly reported pregnancy-specific adverse event (Moro et al., 2025). Secondly, a research team from Shanxi Medical University analyzed VAERS data and reported that, among older adults, RSV vaccination was disproportionately associated with increased reports of inflammatory neurological events, immune thrombocytopenia, and atrial fibrillation (Bao et al., 2025). Thirdly, the University of Bologna team, utilizing the VAERS database, identified disproportionate associations between COVID-19 vaccination and rare but serious adverse events, including myocarditis, Guillain-Barré syndrome, anaphylaxis, and thrombocytopenia, thereby providing critical insights into these rare safety concerns (Santi Laurini et al., 2023). Notably, researchers from the CDC’s Immunization Safety Office also utilized the VAERS database to evaluate the safety of co-administration of COVID-19 and seasonal influenza vaccines (Moro et al., 2024). The results indicated that no new or unexpected safety concerns were identified, and the safety profile of co-administration was consistent with that of individual vaccinations (Moro et al., 2024). These findings provide a scientific basis for further research and public health decision-making regarding co-administration. In conclusion, these studies underscore the invaluable role of VAERS in identifying potential safety signals and evaluating vaccine safety, thereby demonstrating the system’s central contribution to global vaccine safety monitoring.

Building on this foundation, the present study utilizes VAERS data to assess the safety of co-administering RSV, COVID-19, and influenza vaccines in adults aged 60 and older. By applying multiple signal detection algorithms, we aim to identify potential safety signals, providing a comprehensive real-world safety assessment of the co-administration of these three vaccines. This study will supplement the existing literature on the safety of co-administration in older adults and exploring the safety profile and generating hypotheses regarding potential safety signals for the co-administration of these three vaccines.

## 2 Materials and methods

### 2.1 Data source

The data for this study were sourced from VAERS, a system established jointly by the CDC and FDA in 1990. The system monitors vaccine safety and generates hypotheses through spontaneous reports from healthcare providers, vaccine manufacturers, and recipients. VAERS collects data on gender, age, vaccine type, dose batch numbers, AEFI descriptions, and medical history. All AEFI information is standardized and coded using the Medical Dictionary for Regulatory Activities (MedDRA, version 27.0), with each report corresponding to multiple System Organ Classifications (SOC) and Preferred Terms (PT). According to the Code of Federal Regulations, reports are classified as severe or non-severe. Reports involving death, life-threatening illness, hospitalization, prolonged hospitalization, congenital anomalies, or permanent disability events must be classified as severe. For severe reports, VAERS staff request subsequent medical records for further review, and records submitted by vaccine manufacturers undergo a separate, independent follow-up process (United States Code of Federal Regulations, 2014).To ensure data confidentiality, patient information in the reports is de-identified and anonymized before inclusion in the database. As a result, this study was granted an exemption from ethical review by the University of Adelaide’s Human Research Ethics Committee.

### 2.2 Data extraction

We extracted AEFI reports from VAERS covering the period from 3 May 2023, to 1 January 2025. The data were processed through the following steps: (1) reports involving individuals aged ≥60 years at the time of vaccination were selected; (2) duplicate reports were excluded; (3) target vaccines were identified by filtering the “VAX_NAME” field for “RSV,” “COVID-19,” and “INFLUENZA (SEASONAL)”; (4) reports describing vaccination errors without associated AEFIs (e.g., incomplete vaccination procedures) were excluded.

Eligible reports were systematically compiled into an analytical dataset for pharmacovigilance evaluation. We analyzed the characteristics of the included cases and stratified the data based on vaccine co-administration patterns, including: (1) RSV and COVID-19 co-vaccination group, (2) RSV and seasonal influenza co-vaccination group, and (3) RSV, COVID-19, and influenza triple co-administration group. Descriptive statistics were performed for all included reports, summarizing demographic characteristics such as sex, age, geographic region, time interval from vaccination to symptom onset, clinical outcomes, and the proportion of serious AEFI reports. The causality of reported deaths was determined based on the consistency of evidence across autopsy reports, death certificates, and medical documentation.

### 2.3 Statistical analysis

In this study, signal detection for AEFIs was conducted using four disproportionality analysis methods: the reporting odds ratio (ROR), proportional reporting ratio (PRR), Bayesian confidence propagation neural network (BCPNN), and multi-item gamma Poisson shrinker (MGPS). These algorithms are among the most widely applied tools in pharmacovigilance and were chosen for their complementary strengths in signal detection. ROR and PRR are frequentist approaches well-suited to large-scale datasets, whereas BCPNN and MGPS are Bayesian approaches particularly effective for handling low-frequency events and minimizing false positives (Zhu et al., 2025). Collectively, these four algorithms enhance the robustness and reliability of signal detection. No single method is universally applicable; instead, each displays distinct sensitivities and limitations depending on signal frequency, background data processing, and control of false positives (Gravel et al., 2024).

The ROR is a case-control approach that compares the odds of reporting a specific AEFI in vaccinated individuals with the odds of reporting the same AEFI in the unvaccinated group. This method is particularly effective in reducing denominator bias, a crucial factor for accurate risk assessment. The signal detection criteria for ROR require that the number of reports (n) be greater than or equal to 3 and that the 95% confidence interval (CI) lower bound is greater than 1, indicating a statistically positive signal (Xia et al., 2024).

The PRR is a more straightforward yet powerful method, which compares the proportion of reports for a particular AEFI after vaccination to the proportion of reports for the same AEFI after all other vaccines. PRR does not require external data, making it a valuable tool for detecting both known and emerging risk signals. A signal is considered positive using PRR when the following criteria are met: PRR ≥2, χ^2^ ≥ 4, and n ≥ 3, ensuring statistical robustness of the signal (Cutroneo et al., 2023).

The BCPNN is a more advanced method that calculates the information component (IC) using Bayesian posterior probabilities. This method is well-suited for identifying signals from low-frequency events and offers uncertainty intervals for signal reliability. A positive signal in BCPNN is defined when the lower bound of the Information Component (IC025) exceeds 0, indicating a statistically significant association (Sankar et al., 2025).

The MGPS method employs hierarchical Bayesian shrinkage to mitigate the impact of false positives, which is particularly beneficial in large datasets where random noise could otherwise skew the results. It uses global optimization methods to improve the accuracy of signal detection. A positive signal in MGPS is identified when the 95% lower bound of the Expected Bayesian Geometric Mean (EBGM05) exceeds 2, indicating a significant association between the vaccine and AEFI (Xia et al., 2024).

To ensure analytical comprehensiveness and enhance specificity, we implemented a consensus criterion whereby an AEFI was considered a statistically significant potential signal only if it was independently detected by at least two algorithms. This rigorous cross-validation strategy leverages the complementary strengths of the methods and substantially reduces the risk of false positives that may arise from reliance on a single approach, thereby increasing the robustness of signal detection and the credibility of our findings (Fusaroli et al., 2024). Standardized formulas were applied for each method, and calculations were conducted using 2 × 2 contingency tables (see Supplementary Tables S1, S2 for details). All statistical analyses and data processing were performed using Microsoft Excel 2021 and R software (version 4.3.3). As VAERS is a publicly accessible database containing anonymized and de-identified patient information, neither informed consent nor ethical approval was required for this study.

## 3 Results

### 3.1 Baseline characteristics

**FIGURE 1:**
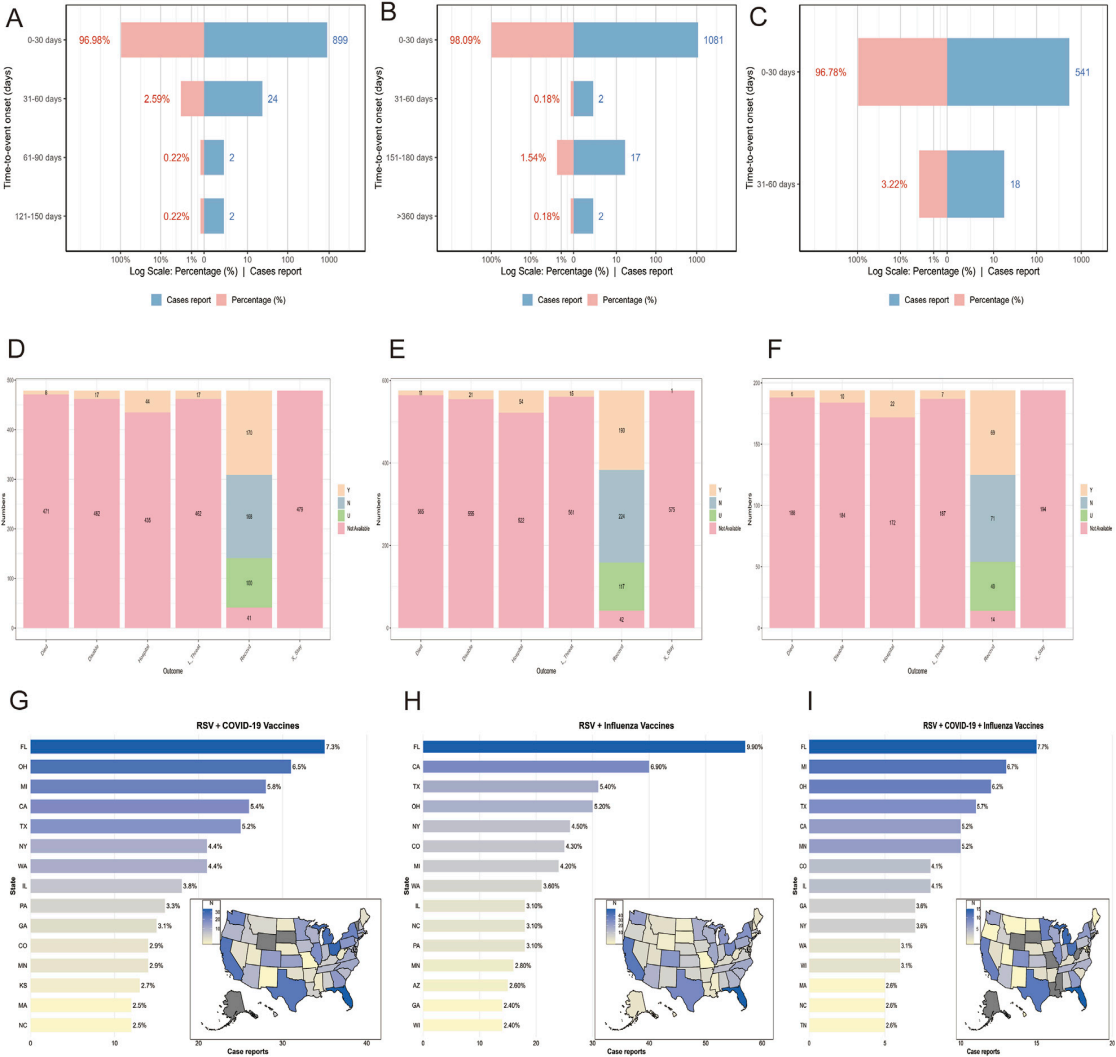
Time to event onset **(A–C)**, clinical outcomes **(D–F)**, and regional distribution of reports **(G–I)** in the RSV–COVID-19 co-administration group, RSV–seasonal influenza co-administration group, and triple vaccination group, respectively. Died: Reports of deaths following vaccination. L_threat: Reports of life-threatening events following vaccination. Hospital: Reports of hospitalization following vaccination. X_stay: Reports of prolonged hospitalization due to vaccine-related adverse events. Disable: Reports of disability following vaccination. Recovered: Reports of recovery from adverse events following vaccination.

Between 3 May 2023, and 1 January 2025, VAERS recorded 479 reports of RSV-COVID-19 co-administration, 576 reports of RSV-seasonal influenza co-administration, and 194 reports of triple vaccination (RSV, COVID-19, and seasonal influenza) in individuals aged 60 and older. Table 1 summarizes the clinical characteristics of these patients. In all three groups, reports from female patients outnumbered those from male patients by approximately two to three times. In terms of age distribution, individuals aged 65–84 years constituted the majority of safety reports across all groups. Regarding the timing of AEFI reports (Figures 1A–C), most AEFIs were reported within 30 days after vaccination: 899 (96.98%) in the RSV-COVID-19 co-administration group, 1,081 (98.09%) in the RSV-seasonal influenza co-administration group, and 541 (96.78%) in the triple vaccination group. The mean onset time of AEFIs was similar across groups (3.94, 3.56, and 3.63 days, respectively), but the standard deviations varied significantly: 10.8 for RSV-COVID-19 co-administration and 17.9 for RSV-seasonal influenza co-administration. The median onset time was 1 day in all groups, with a minimum of 0 days, but the maximum onset time differed considerably among the groups. In terms of severity, the number of serious reports was 55 (11.5%) in the RSV-COVID-19 co-administration group, 72 (12.5%) in the RSV-seasonal influenza co-administration group, and 28 (14.4%) in the triple vaccination group, while non-serious reports accounted for 424 (88.5%), 504 (87.5%), and 166 (85.6%), respectively. For clinical outcomes, recovery was the most frequently reported outcome in all three groups, with 170 (35.5%), 193 (33.5%), and 69 (35.6%) cases, respectively. Hospitalization was the second most frequently clinical outcome and the most frequently reported outcome among serious cases, with 44 (9.2%), 54 (9.4%), and 22 (11.3%) cases, respectively. Other clinical outcomes are detailed in Figures 1D–F. Regarding geographic distribution, Florida had the highest number of reports in all three groups (Figures 1G–I), with 35 (7.3%), 57 (9.9%), and 15 (7.7%) reports, respectively.

**TABLE 1:** Characteristics of reported cases by vaccine co-administration group.

No.	Characteristics	RSV + COVID-19 vaccines	RSV + influenza vaccines	RSV + COVID-19 + influenza vaccines
1	N	479	576	194
2	Sex			
2.1	Males	174 (36.3%)	195 (33.9%)	66 (34.0%)
2.2	Females	334 (62.1%)	379 (65.8%)	127 (65.5%)
2.3	Not reported	2 (0.4%)	2 (0.3%)	1 (0.5%)
3	Age groups			
3.1	60–64	79 (16.5%)	86 (14.9%)	31 (16.0%)
3.2	65–84	360 (75.2%)	445 (77.3%)	148 (76.3%)
3.3	85 plus	40 (8.4%)	45 (7.8%)	15 (7.7%)
4	Onset time			
4.1	Mean (SD)	3.94 (10.8)	3.56 (17.9)	3.63 (8.09)
4.2	Median [Min, Max]	1.00 [0, 139]	1.00 [0, 364]	1.00 [0, 53.0]
4.3	Missing	16 (3.3%)	22 (3.8%)	8 (4.1%)
5	Serious			
5.1	No	424 (88.5%)	504 (87.5%)	166 (85.6%)
5.2	Yes	55 (11.5%)	72 (12.5%)	28 (14.4%)

### 3.2 AEFI mining and signal detection based on SOC level

**FIGURE 2:**
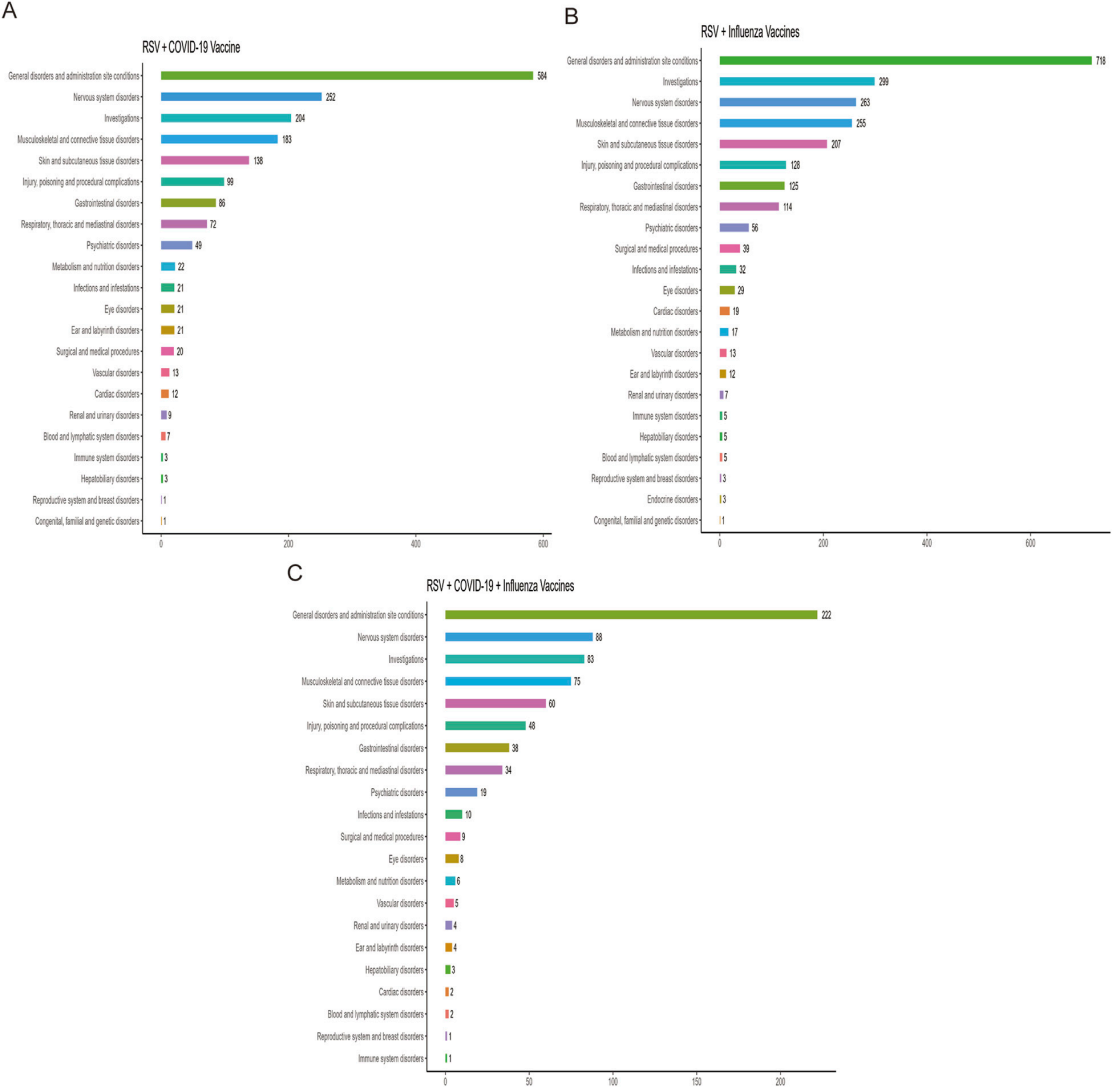
Distribution of adverse events across system organ classes (SOCs) in the RSV–COVID-19 co-administration group **(A)**, RSV–seasonal influenza co-administration group **(B)**, and triple vaccination group **(C)**.

Figure 2 ranks the SOCs based on the number of reported AEFIs. The RSV-COVID-19 co-administration group, the RSV-seasonal influenza group, and the triple vaccination group consisted of 22, 23, and 21 SOCs, respectively. The top three SOCs were consistent across all groups: general disorders and administration site conditions, nervous system disorders, and investigations. Supplementary Table S3 presents the signal detection results at the SOC level for the three vaccination strategies. Using four disproportionality analysis methods, we identified several SOCs with positive signals Table 2. Notably, in addition to commonly observed post-vaccination SOCs, several potential safety signals emerged, including ear and labyrinth disorders in the RSV-COVID-19 co-administration group (n = 21, ROR: 1.89 [1.18–3.01], IC025: 0.14), skin and subcutaneous tissue disorders in the RSV-seasonal influenza group (n = 207, ROR: 1.51 [1.29–1.76], IC025: 0.26), and injury, poisoning and procedural complications in the triple vaccination group (n = 48, ROR: 1.57 [1.16–2.12], IC025: 0.15). These relatively strong signals suggest that the above SOCs may be associated with co-administration and warrant further investigation and ongoing surveillance to assess the safety of combined vaccination strategies comprehensively.

**TABLE 2:** Strength of positive signals for AEFIs at the SOC level.

No.	SOC	N	ROR (95% CI)	IC (IC025)
1	RSV + COVID-19 vaccines			
1.1	Nervous system disorders	252	1.44 (1.23–1.66)	0.42
1.2	General disorders and administration site conditions	585	1.35 (1.22–1.50)	0.25
1.3	Ear and labyrinth disorders	21	3.89 (1.98–7.61)	1.29
1.4	Psychiatric disorders	49	1.53 (1.13–2.07)	0.54
1.5	Skin and subcutaneous tissue disorders	138	1.25 (1.04–1.50)	0.27
1.6	Musculoskeletal and connective tissue disorders	185	1.39 (1.18–1.64)	0.59
2	RSV + Influenza Vaccines			
2.1	Musculoskeletal and connective tissue disorders	255	1.54 (1.34–1.78)	0.53
2.2	General disorders and administration site conditions	718	1.25 (1.14–1.37)	0.22
2.3	Injury, poisoning and procedural complications	128	1.28 (1.06–1.55)	0.43
2.4	Skin and subcutaneous tissue disorders	251	1.51 (1.29–1.76)	0.65
3	RSV + COVID-19 + Influenza Vaccines			
3.1	Skin and subcutaneous tissue disorders	60	1.36 (1.04–1.78)	0.41
3.2	Musculoskeletal and connective tissue disorders	75	1.44 (1.11–1.89)	0.43
3.3	Injury, poisoning and procedural complications	48	1.57 (1.16–2.12)	0.59

### 3.3 AEFI mining and signal detection based on PT level

**FIGURE 3:**
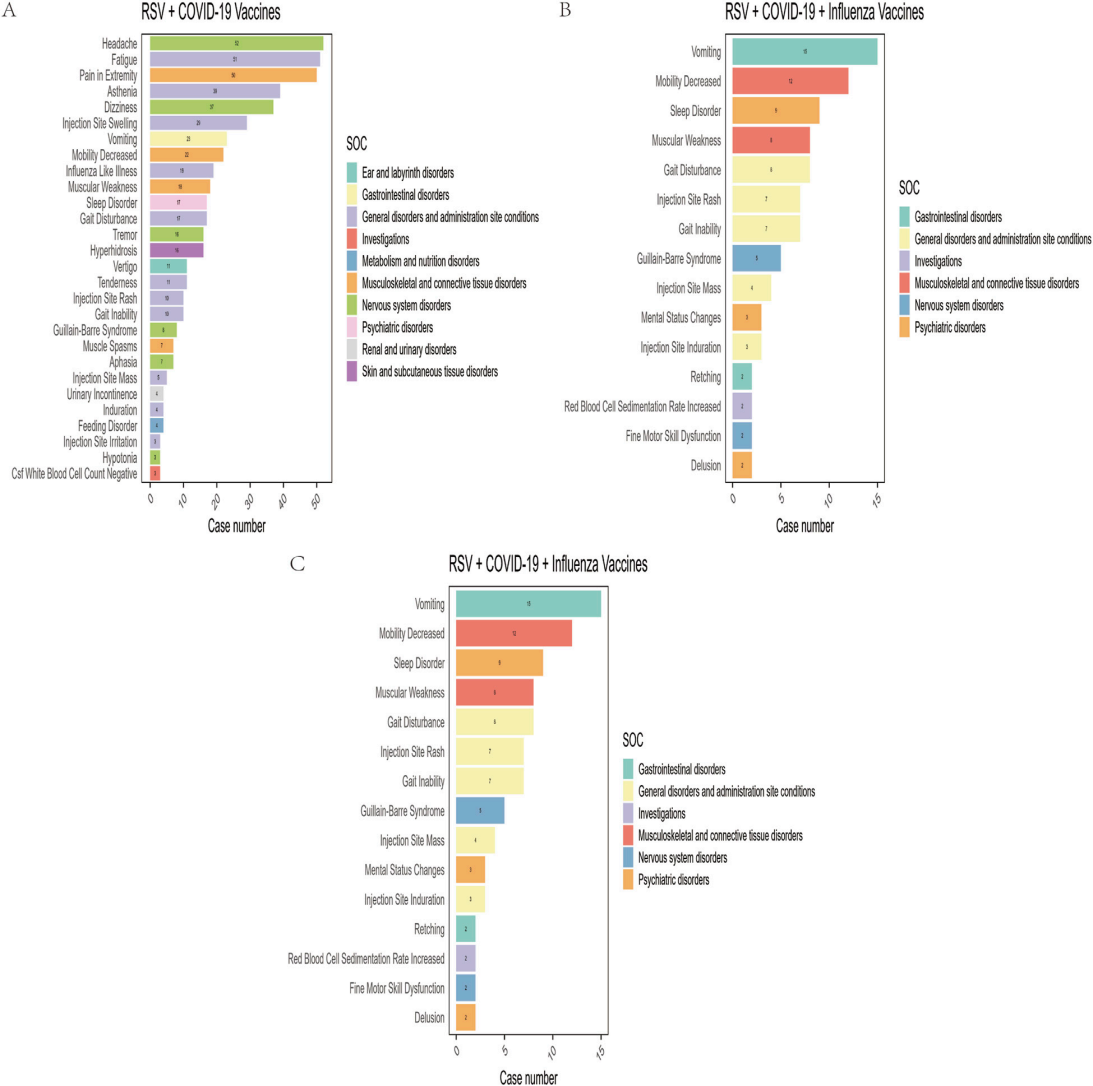
Preferred terms (PTs) with positive signals of adverse events in the RSV–COVID-19 co-administration group **(A)**, RSV–seasonal influenza co-administration group **(B)**, and triple vaccination group **(C)**.

In this study, after excluding missing data and non-relevant signals, a total of 28, 27, and 15 positively signaled PTs were identified in the RSV-COVID-19, RSV-influenza, and triple co-administration groups, respectively, based on the criteria of four signal detection algorithms (Supplementary Table S4). Figure 3 presents the PTs categorized by their respective SOCs and ranked by reporting frequency. The top five most frequently reported PTs were as follows: for the RSV-COVID-19 group—headache (n = 52), fatigue (n = 51), pain in extremity (n = 50), asthenia (n = 39), and dizziness (n = 37); for the RSV-influenza group—pain (n = 68), pain in extremity (n = 67), erythema (n = 49), injection site pain (n = 44), and injection site swelling (n = 38); and for the triple co-administration group—pain in extremity (n = 22), vomiting (n = 15), pruritus (n = 12), mobility decreased (n = 12), and sleep disorder (n = 9).

**FIGURE 4:**
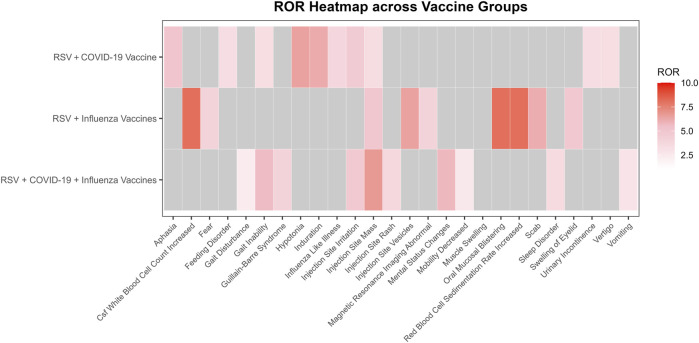
Heatmap of reporting odds ratios (ROR) for adverse events following different vaccine co-administration groups.

Among the four signal detection algorithms, the ROR algorithm identified the highest number of positive signals and is considered to provide more substantial evidence, with higher ROR values indicating more robust associations. Based on this, the top 10 PTs with the highest ROR values were selected for each group (Figure 4) (Sakaeda et al., 2013). In addition to commonly observed vaccine-related AEFIs, several rare but high-ROR PTs warrant attention. These include aphasia in the RSV-COVID-19 group, oral mucosal blistering, increased red blood cell sedimentation rate, and increased CSF white blood cell count in the RSV-influenza group, as well as mental status changes and gait inability in the triple co-administration group. Although these PTs were reported infrequently, their elevated ROR values suggest potential safety signals that merit further investigation in future studies.

### 3.4 AEFI mining and signal detection based on reported severity

**FIGURE 5:**
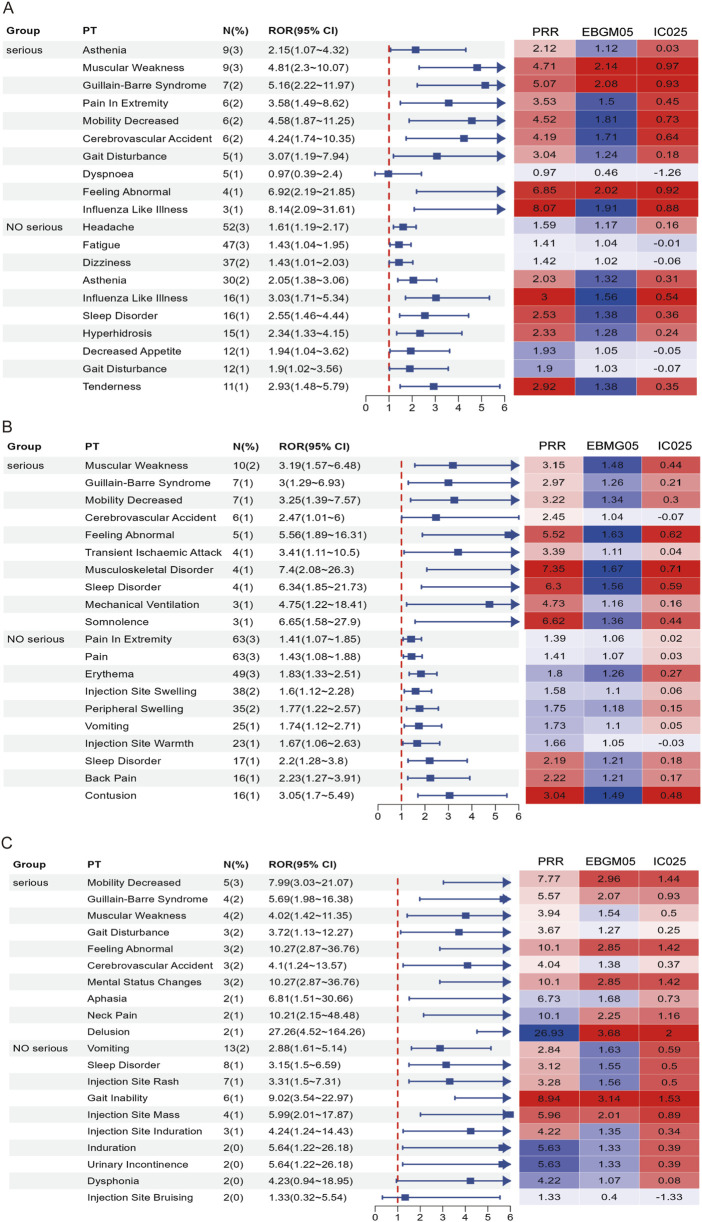
Forest plots and heatmaps illustrate the top 10 most frequently reported PTs and the corresponding signal strength in serious and non-serious reports for the RSV–COVID-19 co-administration group **(A)**, RSV–seasonal influenza co-administration group **(B)**, and triple vaccination group **(C)**. In the heatmaps, red indicates that the signal meets the criteria of the respective algorithm, while blue indicates that it does not. A deeper red color represents a stronger signal.

In this study, AEFIs were stratified by severity (severe vs. non-serious) and analyzed using four signal detection algorithms—ROR, PRR, BCPNN, and MGPS. After excluding missing data, product issues, and non-associated signals, a total of 115, 117, and 58 positively signaled PTs were identified in the serious reports of the RSV-COVID-19, RSV-influenza, and triple co-administration groups, respectively (Supplementary Table S4), In contrast, 223, 255, and 102 PTs were detected in the non-serious reports of the respective groups (Supplementary Table S5). Figure 5 presents the top 10 most frequently reported PTs in each group by severity, along with their RORs (95% CIs) and a signal heatmap based on the four algorithms.

In serious reports, although overall frequency was relatively low, many PTs exhibited signal strengths substantially exceeding threshold values across multiple algorithms, indicating positive signals that suggest rare but potentially significant risks, particularly involving musculoskeletal, neurological, and psychiatric events. In the RSV-COVID-19 group, asthenia was the most frequently reported serious PT and met the criteria of ROR, PRR, and BCPNN, while influenza-like illness, gait disturbance, cerebrovascular accident, mobility decreased, and pain in extremity demonstrated stronger signals; muscular weakness, Guillain-Barré syndrome, and feeling abnormal satisfied all four algorithms, indicating a stronger potential association. In the RSV-influenza group, muscular weakness was the most frequent PT, and other positive signals included musculoskeletal disorder, sleep disorder, somnolence, and feeling abnormal, with all but cerebrovascular accident meeting multi-algorithm thresholds. In the triple co-administration group, mental status changes were the most frequently reported PT, and along with feeling abnormal, mobility decreased, and Guillain-Barré syndrome, met all four algorithm criteria; muscular weakness, gait disturbance, and cerebrovascular accident met three. In non-serious reports, common PTs such as headache, fatigue, and pain in extremity showed high reporting frequency but low signal strength, indicating they are likely expected post-vaccination reactions. However, specific PTs—including influenza-like illness, tenderness, sleep disorder, hyperhidrosis, contusion, vomiting, and injection site rash—had lower frequency but higher signal strength. Specific events, such as gait inability and injection site mass, met all four algorithm thresholds, indicating positive signals and raising potential safety concerns. Overall, while the majority of non-serious AEFIs appeared to be common and anticipated, serious AEFIs revealed several rare but strong positive signals that warrant further clinical evaluation and mechanistic investigation. These findings contribute valuable evidence to inform optimization of vaccine safety strategies in older adults.

## 4 Discussion

Overall, our analysis demonstrated that co-administration of RSV, COVID-19, and seasonal influenza vaccines in older adults was generally safe, with most AEFIs being mild and expected, such as fatigue, injection site pain, and headache. This is consistent with the generally reported acceptable safety and immunogenicity patterns in other vaccine co-administration studies. For instance, Moro et al., using VAERS data, described AEFIs following the co-administration of the influenza and COVID-19 vaccines, finding similar mild adverse reactions, primarily injection site pain and fatigue, with no significant safety concerns observed in the short term (Moro et al., 2024). However, we also identified several positive signals, including some rare but potentially clinically significant events, particularly within the neurologic and psychiatric disorder SOCs, which align with observations in other studies. Bao et al., using VAERS data to monitor post-market AEFIs for the RSV vaccine, also identified issues related to neurological adverse events, such as Guillain-Barré syndrome (Bao et al., 2025). Additionally, Hause et al. collected AEFI data from five large-scale COVID-19 vaccination sites, where 64 cases of anxiety were reported (Hause et al., 2021). AEFIs observed with co-administration may also occur with individual vaccines. Although AEFIs can impact individual patients, the co-administration strategy is generally safe for the elderly population. Dacso et al. demonstrated through a randomized controlled trial that co-administration of the mRNA COVID-19 vaccine with the influenza vaccine did not increase the incidence of adverse events compared to separate administration. This strategy is increasingly being adopted to improve vaccination coverage (Dacso, 2025). Future studies should aim to prospectively validate these signals and more robustly assess causality to guide safer and more effective vaccination policies for the elderly population.

Regarding the baseline characteristics of co-administration, the proportion of females was significantly higher than that of males. This phenomenon is primarily attributed to the fact that females generally exhibit stronger antibody responses in immune reactions (Klein et al., 2010; Furman et al., 2014), making them more likely to experience more pronounced AEFIs, such as fever, pain, and inflammation. These findings are consistent with past research. Rosenblum et al. observed that, during the first 6 months of the U.S. vaccination program, the incidence of adverse events following the administration of two mRNA vaccines was significantly higher in females (Rosenblum et al., 2022). In addition, survey data from Green et al. also show that the incidence of AEFIs following COVID-19 vaccination is higher in women than in men (Green et al., 2022). Although women face a higher risk of adverse reactions post-vaccination, this risk does not contraindicate vaccination. Therefore, in clinical practice, it is essential to clearly inform recipients during the informed consent process and further explore the possibility of adjusting co-administration doses based on gender to minimize the occurrence of adverse events.

Regarding the timing of AEFIs, over 96% of reports occurred within 30 days post-vaccination, with a median onset time of 1 day. This indicates that most AEFIs occur in the short term after vaccination, aligning with the time window for the activation of innate immunity and early adaptive immunity (Hervé et al., 2019; Pollard and Bijker, 2021). The CDC has stated in its clinical trials for COVID-19, seasonal influenza, and RSV vaccines that the vast majority of local and systemic AEFIs are mild to moderate, typically occurring within 1–2 days post-vaccination and resolving on their own within 1–3 days (Centers for Disease Control and Prevention, 2024; Centers for Disease Control and Prevention, 2025a; Centers for Disease Control and Prevention, 2025b). However, unlike previous studies, our research found that the standard deviation for the RSV-influenza co-administration group was significantly higher than that for the other two groups. In prior research, Clark et al. did not observe a significant increase in the standard deviation when evaluating the safety and immunogenicity of co-administering the RSV and seasonal influenza vaccines (Clark et al., 2024). This difference may stem from the fact that RSV and influenza vaccines elicit distinct patterns of immune activation, with differences in the timing and magnitude of innate versus adaptive immune responses. Recent systems immunology studies have demonstrated that innate and adaptive responses do not always synchronize, and their temporal asynchrony can influence reactogenicity profiles. For instance, Schramm et al. showed that early innate activation following vaccination can shape, but also be temporally misaligned with, the subsequent adaptive immune response (Schramm et al., 2023). Similarly, Kazmin et al. highlighted that innate immunity may exhibit “memory-like” features after vaccination, leading to enhanced or altered responses upon subsequent stimulation, which can further modify the timing of onset of adverse events (Kazmin et al., 2023). Moreover, Hellgren et al. demonstrated that preexisting adaptive immune memory can modulate early innate activation, reinforcing the bidirectional crosstalk and temporal complexity between the two arms of immunity (Hellgren et al., 2024). Taken together, these findings suggest that in the RSV–influenza co-administration group, AEFIs may arise at more dispersed time points, with early events likely mediated by innate pathways and later events driven by adaptive responses. Clinical evidence from recent co-administration trials supports this biological plausibility, showing overall acceptable safety profiles but heterogeneous reactogenicity timing (Buynak et al., 2024; Chandler et al., 2024). Furthermore, in all three groups, some AEFIs occurred more than 30 days after vaccination, with the longest lasting over 360 days. This indicates that rare delayed adverse events may still occur long after vaccination. Therefore, to effectively monitor delayed AEFIs, it is recommended to establish a long-term follow-up system to identify and address potential immune responses promptly.

At the SOC level, AEFIs were primarily concentrated in systemic disorders, administration site reactions, neurological diseases, and various abnormal laboratory findings. Previous studies have shown that the most commonly reported AEFIs in co-administration are typically mild to moderate injection site pain (Toback et al., 2022; Naficy et al., 2024). For example, Naficy et al. found that the incidence of injection site pain was higher in the co-administration group (Naficy et al., 2024). Toback et al. also noted that the reactogenicity of co-administration, including injection site pain, fatigue, and muscle pain, was more common than with the individual administration of the COVID-19 vaccine (Toback et al., 2022). Additionally, Moro et al., using VAERS data, further validated the higher frequency of reactogenicity associated with co-administration (Moro et al., 2024). In addition to these expected reactions, this study also identified some potential safety signals. For example, in the RSV-COVID-19 co-administration group, ear and vestibular disorders were detected as positive signals. Dorney et al. collected AEFI reports following COVID-19 vaccination and found that the risk of new-onset tinnitus was higher after among individuals who received the COVID-19 vaccine (Dorney et al., 2023). Given the distinct immune mechanisms of the RSV and COVID-19 vaccines, this may lead to an enhanced reactogenicity in the ear and vestibular systems, thereby increasing the risk of adverse reactions such as tinnitus. A similar situation was observed in the RSV-seasonal influenza co-administration group, where skin and subcutaneous tissue disorders were identified as positive signals. In the triple co-administration group, most positive signals were concentrated in SOCs related to injuries, poisonings, and surgical complications. This pattern reflects known post-vaccination reactions, and other studies have reported similar findings. For example, a Phase III clinical trial evaluating the safety of co-administering the RSV and seasonal influenza vaccines in individuals aged 65 and older found that the incidence of skin and subcutaneous tissue disorders (such as rashes and injection site reactions) was slightly higher in the co-administration group than in the individual vaccine groups. However, all reactions were mild to moderate (Rizkalla et al., 2023). While these SOCs met the criteria of multiple algorithms and were considered positive signals, they are broadly consistent with known post-vaccination reactions, demonstrating the effectiveness of our signal detection method.

At the PT level, common AEFIs in the RSV-COVID-19 co-administration group, such as headache, fatigue, and injection site pain, were consistent with previous studies on these vaccines (Centers for Disease Control and Prevention, 2025c; Centers for Disease Control and Prevention, 2025b). The relatively higher reporting frequency of sleep disorders in the triple co-administration group may be related to increased discomfort caused by multiple vaccinations. In a cross-sectional study, Al Katatbeh et al. found that the occurrence of sleep disturbances and fragmented nighttime sleep was associated with the number of vaccine doses administered (Al Katatbeh et al., 2024). Similarly, in a cohort study, Kim et al. observed a positive correlation between the number of COVID-19 vaccine doses and the incidence of sleep disorders (Kim et al., 2024). These findings suggest that multiple vaccinations may have a cumulative effect on symptoms. Additionally, this study observed some low-frequency reported AEFIs. Despite their lower occurrence, the corresponding high ROR values suggest a potentially strong association, warranting further attention. For example, aphasia was reported as an AEFI in the RSV-COVID-19 co-administration group, indicating that co-administration may have a potential adverse effect on the nervous system. A meta-analysis by Riccò et al. highlighted that respiratory viral infections could exhibit neurotropism, similar to RSV infections, where the virus may directly invade the central nervous system, leading to neuronal damage and resulting in neurological symptoms such as aphasia (Riccò et al., 2024). This mechanism suggests that co-administration could trigger similar side effects by enhancing the immune response or inducing immune-mediated neurological damage. In the RSV-seasonal influenza co-administration group, we also observed oral mucosal vesicles as an AEFI, which may be exacerbated by pre-existing oral lesions in the patients. Riad et al., through database analysis, found that oral herpes was significantly more common after COVID-19 vaccination compared to influenza vaccination (Riad et al., 2023). De Simone et al. reviewed cases of herpes outbreaks following vaccination and found that individuals with a history of herpes may develop new erosions and vesicular lesions after receiving the influenza vaccine (De Simone et al., 2008). This phenomenon may be related to the disruption of the balance between the immune system and potential underlying autoimmune diseases after vaccine stimulation, thereby triggering disease manifestation. It is also possible that the vaccine induces a hypersensitive reaction in the oral mucosa, leading to these symptoms. In the triple co-administration group, some patients reported symptoms of gait impairment, suggesting that co-administration may trigger neuromuscular complications. Kim et al. summarized various factors contributing to gait impairment, including biological, environmental, and psychological factors. Some patients may experience more pronounced physical discomfort due to side effects such as muscle soreness and fatigue following vaccination (Kim et al., 2021). Edwards et al. suggested that some patients’ excessive focus on the side effects of co-administered vaccines, coupled with psychological suggestion, could heighten concerns and anxiety about their health, intensifying their perception of discomfort (Edwards et al., 2012; Kim et al., 2021). This psychological response may further activate abnormal cognitive and neurological mechanisms, leading to the onset of neuromuscular complications such as gait impairment. Therefore, the co-administration of RSV, COVID-19, and influenza vaccines may lead to a variety of adverse reactions, including neurological, oral mucosal, and neuromuscular complications. Multiple vaccinations may result in a cumulative effect of symptoms, highlighting the need for further attention and validation of their potential immune-mediated damage and the role of psychological factors, especially in high-risk populations such as the elderly.

In the serious adverse event reports for co-administered vaccines, the RSV-COVID-19 co-administration group showed particularly prominent signal values for Influenza-Like Illness and Cerebrovascular Accident, with higher frequencies among the top ten PTs. This suggests that these two adverse events require special attention. According to the CDC’s definition, Influenza-like illness refers to acute respiratory infection symptoms, including a temperature of 100°F (approximately 37.8 °C) or higher, along with cough and/or sore throat. Common viral pathogens that cause this condition include RSV and SARS-CoV-2, among others (Centers for Disease Control and Prevention, 2025d). The CDC states that the RSV vaccine can be co-administered with other adult vaccines during the same session, but it may increase the incidence of common side effects. Therefore, in the context of co-administering the RSV and COVID-19 vaccines, the immune activation effects induced by both vaccines may have a synergistic effect, leading to more pronounced influenza-like symptoms than those observed with the individual administration of either vaccine (Centers for Disease Control and Prevention, 2025e). Regarding Cerebrovascular Accident, a study by Greinacher et al. found that COVID-19 vaccination may trigger thrombocytopenia and thrombosis-related complications, significantly increasing the risk of intracranial thrombosis and bleeding (Greinacher et al., 2022). In a self-controlled case series study, Lu et al. assessed the risk of stroke following the administration of the bivalent COVID-19 vaccine (Lu et al., 2024). The study found no significant association between the bivalent COVID-19 vaccine and the risk of stroke. However, when co-administering high-dose or adjuvanted influenza vaccines, the researchers observed a slight increase in the risk of non-hemorrhagic stroke within a 22- to 42-day risk window (Lu et al., 2024). The study suggests that although co-administration may slightly increase the risk of stroke, the clinical significance of this association requires further investigation. While there is currently no direct evidence of a clear association between the RSV vaccine and cerebrovascular events, in the context of co-administration, cerebrovascular events are reported more frequently with significant signal strength in AEFI reports, indicating the need for enhanced monitoring and mechanistic research of such events. It is noteworthy that Guillain-Barré syndrome exhibited a strong signal in the cross-validation using the four algorithms, indicating that this adverse event warrants special attention. Guillain-Barré syndrome, a rare autoimmune peripheral neuropathy, has been reported as an infrequent but serious adverse reaction to the RSV, COVID-19, and influenza vaccines (Centers for Disease Control and Prevention, 2024; Centers for Disease Control and Prevention, 2025b; Centers for Disease Control and Prevention, 2025c). Research by Moro et al. suggests that co-administration of vaccines may lead to heightened reactogenicity (Moro et al., 2024). Therefore, as a rare but serious adverse event triggered by the combination of all three vaccines, Guillain-Barré syndrome should be closely monitored for potential risks following co-administration. In the RSV-seasonal influenza co-administration group, muscular weakness was the most frequently reported PT, while musculoskeletal disorder displayed the strongest signal strength. Previous studies have shown that influenza vaccination alone typically does not induce or exacerbate symptoms of muscular weakness (Zinman et al., 2009; Auriel et al., 2011). Relatedly, sleep disorders and somnolence also exhibited significant signal strength, suggesting that sleep-related issues, particularly somnolence, should be closely monitored in the RSV-seasonal influenza co-administration group. Tsai et al., through a clinical database study on AEFIs following H1N1 influenza vaccine Pandemrix administration, found a significantly increased risk of narcolepsy, although no evidence currently suggests an association between other influenza vaccines and somnolence symptoms (Tsai et al., 2011). Wu et al. conducted a cross-sectional study, finding that among middle-aged and older European individuals who received co-administration of COVID-19 and influenza vaccines, the incidence of sleep disorders was higher, and this co-administration may exacerbate the symptom (Wu et al., 2024). Therefore, it is recommended that sleep disorders and somnolence symptoms be closely monitored in the RSV-seasonal influenza co-administration group, as potential serious AEFIs requiring attention. In the severe AEFI reports for the triple vaccination group, mental status changes were the most frequently reported PT, while feeling abnormal exhibited the strongest signal strength. This suggests that co-administration of multiple vaccines may have a significant impact on the mental system, warranting increased attention to related events in subsequent monitoring. Notably, cerebrovascular accidents were detected across all three co-administration groups. Although the report frequency for this PT was relatively low in each group, its significant signal strength indicates that the potential risk of this event should not be overlooked. Pérez-Rubio et al. reported that influenza vaccination was associated with a reduced risk of cerebrovascular events, suggesting a potential protective effect on cardiovascular outcomes (Pérez-Rubio et al., 2021). However, in this study, cardiovascular-related AEFIs emerged as a prominent type of severe reaction in all three groups. Existing studies have not specifically elucidated the impact of co-administration on the cardiovascular system. Nevertheless, our findings suggest that co-administration may interfere with the protective effects of individual vaccines and may even exacerbate the risk of cardiovascular and cerebrovascular events. Therefore, it is recommended that long-term monitoring and evaluation of these symptoms continue in future vaccine safety assessments.

In the reports of non-serious adverse events, the RSV-COVID-19 co-administration group showed higher frequencies of headache, fatigue, and dizziness, while the RSV-seasonal influenza co-administration group reported increased muscle pain. Although the signal strength for these events was relatively weak, they are well-known common vaccine-related AEFIs with a certain degree of predictability (Centers for Disease Control and Prevention, 2025b; Centers for Disease Control and Prevention, 2025c). A similar phenomenon was observed in data from the U.S. Department of Health and Human Services, which indicated that co-administration of influenza, COVID-19, and RSV vaccines may increase the risk of adverse events such as headache, fatigue, and injection-site pain. However, these reactions are generally mild and, in most cases, resolve spontaneously within a few days (United States Department of Health and Human Services, 2025). These findings are consistent with previous vaccine safety studies, further supporting the predictability and acceptability of such AEFIs, suggesting that excessive concern is unwarranted. Nevertheless, routine monitoring following vaccination remains advisable to ensure recipient safety. Notably, some adverse events demonstrated high signal strength across multiple detection algorithms, indicating a potentially stronger association with vaccination. For example, in the RSV–seasonal influenza co-administration group, contusion exhibited a powerful signal. Cook et al. reported that the occurrence of contusion may be related to improper injection technique, inappropriate needle length, or suboptimal site selection during co-administration (Cook, 2015). Similarly, Barnes et al. noted that in some older adults, the use of excessively long needles for influenza vaccination may result in deep intramuscular penetration, thereby causing shoulder contusion (Barnes et al., 2012). This phenomenon underscores the importance of selecting the proper site, adhering to standardized injection techniques, and individualizing the needle length during co-administration to minimize mechanical injury caused by the injection itself. Notably, sleep disorders were detected as signals across all three co-administration groups, suggesting that they may represent a consistent pattern of non-serious adverse events following RSV vaccine co-administration. Accordingly, continued monitoring and evaluation of this symptom are recommended in future vaccine safety assessments.

This study provides large-scale, real-world evidence on the safety of RSV, COVID-19, and seasonal influenza vaccine co-administration in older adults. However, several important limitations must be considered. As a passive surveillance system, VAERS is subject to under-reporting, over-reporting, and reporting biases. The data lack denominator information and an unvaccinated control group, preventing the calculation of incidence rates. Confounders such as comorbidities, concomitant medications, and prior infection history could not be adjusted for. Most importantly, disproportionality analysis can only identify statistical associations and cannot establish causality. Therefore, the findings of this study are strictly hypothesis-generating. The detected signals indicate areas for further investigation but do not constitute evidence of a causal link between vaccination and adverse events. Future prospective cohort studies or case-control studies are required to validate these signals.

## 5 Conclusion

This study, based on the VAERS database, establishes a multidimensional safety evaluation framework for the combined administration of RSV, COVID-19, and seasonal influenza vaccines in individuals aged 60 years and above. The results indicate that the majority of AEFIs caused by the three combination vaccination regimens are non-serious and self-limiting reactions, with a higher prevalence of reported AEFIs in females. The types of common AEFIs are generally consistent with the safety profiles of previous vaccines, supporting the overall safety of the combination vaccination. However, the study also identified several rare but noteworthy potential high-risk signals, including neurologic events (such as aphasia, Guillain-Barré syndrome), psychiatric events (such as sleep disturbances, altered mental status, drowsiness), systemic and muscular events (such as gait disturbances, muscle weakness), and cardiovascular events reported in severe cases. These findings provide important clues for subsequent mechanistic exploration and real-world monitoring studies. It must be stressed that this analysis is exploratory and cannot establish causality. The identified signals may be affected by confounding and the inherent biases of passive surveillance. Consequently, our results should not be taken as proof of a causal link between vaccine co-administration and these adverse events. We strongly advocate for rigorous investigation of these potential signals in robust observational studies that control for confounding. As co-administration strategies are rolled out, ongoing long-term safety monitoring is vital. Further studies are required to validate these findings and enhance our understanding of the safety profile in older adults.

## Data Availability

The original contributions presented in the study are included in the article/Supplementary Material, further inquiries can be directed to the corresponding author.
