# Supplementary material for: Real-world feasibility of co-administration of RSV, COVID-19, and influenza vaccines in older adults: a VAERS-based analysis
Source: Front Pharmacol. 2025 Oct 20;16:1682119. doi: 10.3389/fphar.2025.1682119 (PMC12580320; doi:10.3389/fphar.2025.1682119)
Supplement: Supplementary file 1 [file Table1.docx]

**Supplementary Information and Data**

Safety of the RSV Vaccine When Co-administered with COVID-19 and Seasonal Influenza Vaccines: An Analysis Based on the Vaccine Adverse Event Reporting System (VAERS)

**Table of content**

**Table S1:** Formulas and signal detection criterias for reporting odds ratio (ROR), proportional reporting ratio (PRR) and bayesian confidence propagation neural network (BCPNN)

**Table S2:** Fourfold table of disproportionality method.

**Table S3.** SOC-level distribution of AEFIs and signal strength across three co-administration vaccination groups.

**Table S4:** PT-level distribution and signal strength of reported adverse events with positive signals across the three co-administration vaccination groups.

**Table S5:** Distribution and signal strength of adverse events in serious reports across the three co-administration vaccination groups.

**Table S6:** Distribution and signal strength of adverse events in non-serious reports across the three co-administration vaccination groups.

**Table S1:** Formulas and signal detection criterias for reporting odds ratio (ROR), proportional reporting ratio (PRR) and bayesian confidence propagation neural network (BCPNN)

| **Algorithms** | **Equation** | **Threshold** |
| --- | --- | --- |
| ROR | ROR=$\frac{a/c}{b/d}$ | $N\geq3$ |
|  | SE(lnROR)=$\sqrt{\frac{1}{a}+\frac{1}{b}+\frac{1}{c}+\frac{1}{d}}$ | ROR$\geq3$ |
|  | 95%CI=$e^{ln(ROR)\pm1.96se}$ | 95%CI(lower limit)＞1 |
| PRR | PRR=$\frac{a/(a+b)}{c/(c+d)}$ | PRR≥2,N$\geq3$ |
|  | X^2^=$\frac{(a+b+c+d){(ad-bc)}^{2}}{(a+b)(c+d)(a+c)(b+a)}$ | X^2^$\geq4$ |
| BCPNN | IC=log_2_$\frac{a(a+b+c+d)}{(a+b)(a+c)}$ | IC025＞0 |
|  | 95%CI=E(IC)$\pm2\sqrt{V(IC)}$ |  |
| MGPS | EBGM=$\frac{a(a+b+c+d)}{(a+c)(a+b)}$ | EBGM05＞2 |
|  | SE(lnEBGM)=$\sqrt{\frac{1}{a}+\frac{1}{b}+\frac{1}{c}+\frac{1}{d}}$ |  |
|  | 95%CI=$e^{ln(EBGM)\pm1.96\mathrm{se}}$ |  |

Notes: Equation: a, number of reports containing both the target vaccine and target adverse vaccine reaction; b, number of reports containing other adverse vaccine reaction of the target vaccine; c, number of reports containing the target adverse vaccine reaction of other vaccines; d, number of reports containing other vaccines and other adverse vaccine reactions.

Abbreviations: ROR, reporting odds ratio; 95%CI, 95% confidence interval; PRR, proportional reporting ratio; N, the number of reports; X^2^, chi-squared; BCPNN, bayesian confidence propagation neural network; IC, information component; IC025, the lower limit of 95%CI, of the IC; E(IC),the IC expectations; V(IC), the variance of IC; EBGM, empirical Bayesian geometric mean; EBGM05, the lower limit of 95% CI of EBGM.

**Table S2:** Fourfold table of disproportionality method.

|  | **Target adverse event reported** | **Other adverse event reported** | **Total** |
| --- | --- | --- | --- |
| Target vaccine | a | b | a+b |
| Other vaccine | c | d | c+d |
| Total | a+c | b+d | a+b+c+d |

**Table S3.** SOC-level distribution of AEFIs and signal strength across three co-administration vaccination groups.

| **RSV＋COVID-19 Vaccine** | | | | | |
| --- | --- | --- | --- | --- | --- |
| SOC | N | ROR(95%Cl) | PRR(X^2^) | EBGM(EBGM05) | IC(IC025) |
| General disorders and administration site conditions | 584 | 1.35 ( 1.22 - 1.5 ) | 1.24 ( 33.3 ) | 1.22 ( 1.12 ) | 0.28 ( 0.14 ) |
| Nervous system disorders | 252 | 1.44 ( 1.25 - 1.66 ) | 1.38 ( 26.45 ) | 1.34 ( 1.19 ) | 0.42 ( 0.22 ) |
| Investigations | 204 | 0.54 ( 0.47 - 0.63 ) | 0.59 ( 66.05 ) | 0.61 ( 0.54 ) | -0.7 ( -0.92 ) |
| Musculoskeletal and connective tissue disorders | 183 | 1.39 ( 1.18 - 1.64 ) | 1.35 ( 16.21 ) | 1.31 ( 1.15 ) | 0.39 ( 0.16 ) |
| Skin and subcutaneous tissue disorders | 138 | 1.25 ( 1.04 - 1.5 ) | 1.23 ( 5.72 ) | 1.21 ( 1.04 ) | 0.27 ( 0.01 ) |
| Injury, poisoning and procedural complications | 99 | 1.28 ( 1.03 - 1.58 ) | 1.26 ( 5.09 ) | 1.24 ( 1.03 ) | 0.31 ( 0 ) |
| Gastrointestinal disorders | 86 | 1.08 ( 0.86 - 1.35 ) | 1.07 ( 0.42 ) | 1.07 ( 0.88 ) | 0.09 ( -0.23 ) |
| Respiratory, thoracic and mediastinal disorders | 72 | 0.8 ( 0.62 - 1.02 ) | 0.8 ( 3.36 ) | 0.82 ( 0.67 ) | -0.29 ( -0.64 ) |
| Psychiatric disorders | 49 | 1.53 ( 1.13 - 2.07 ) | 1.51 ( 7.62 ) | 1.45 ( 1.13 ) | 0.54 ( 0.1 ) |
| Metabolism and nutrition disorders | 22 | 1.2 ( 0.77 - 1.86 ) | 1.2 ( 0.64 ) | 1.18 ( 0.81 ) | 0.23 ( -0.4 ) |
| Ear and labyrinth disorders | 21 | 1.89 ( 1.18 - 3.01 ) | 1.88 ( 7.37 ) | 1.75 ( 1.18 ) | 0.8 ( 0.14 ) |
| Eye disorders | 21 | 1.03 ( 0.66 - 1.61 ) | 1.03 ( 0.01 ) | 1.02 ( 0.7 ) | 0.04 ( -0.61 ) |
| Infections and infestations | 21 | 0.18 ( 0.11 - 0.27 ) | 0.19 ( 77.87 ) | 0.2 ( 0.14 ) | -2.32 ( -2.95 ) |
| Surgical and medical procedures | 20 | 0.5 ( 0.32 - 0.79 ) | 0.51 ( 9.25 ) | 0.53 ( 0.36 ) | -0.91 ( -1.56 ) |
| Vascular disorders | 13 | 0.66 ( 0.38 - 1.16 ) | 0.66 ( 2.12 ) | 0.68 ( 0.43 ) | -0.55 ( -1.35 ) |
| Cardiac disorders | 12 | 0.31 ( 0.17 - 0.55 ) | 0.31 ( 17.82 ) | 0.33 ( 0.21 ) | -1.58 ( -2.4 ) |
| Renal and urinary disorders | 9 | 0.77 ( 0.39 - 1.52 ) | 0.77 ( 0.57 ) | 0.79 ( 0.45 ) | -0.35 ( -1.29 ) |
| Blood and lymphatic system disorders | 7 | 0.77 ( 0.36 - 1.66 ) | 0.77 ( 0.44 ) | 0.79 ( 0.41 ) | -0.35 ( -1.4 ) |
| Hepatobiliary disorders | 3 | 0.9 ( 0.28 - 2.93 ) | 0.9 ( 0.03 ) | 0.91 ( 0.34 ) | -0.14 ( -1.65 ) |
| Immune system disorders | 3 | 0.42 ( 0.13 - 1.33 ) | 0.42 ( 2.31 ) | 0.44 ( 0.17 ) | -1.18 ( -2.65 ) |
| Congenital, familial and genetic disorders | 1 | 10.81 ( 0.68 - 172.98 ) | 10.81 ( 4.45 ) | 5.9 ( 0.58 ) | 2.56 ( -0.07 ) |
| Reproductive system and breast disorders | 1 | 0.4 ( 0.05 - 2.95 ) | 0.4 ( 0.87 ) | 0.42 ( 0.08 ) | -1.25 ( -3.36 ) |
| **RSV + Influenza Vaccines** | | | | | |
| General disorders and administration site conditions | 718 | 1.25 ( 1.14 - 1.37 ) | 1.17 ( 22.04 ) | 1.15 ( 1.07 ) | 0.2 ( 0.08 ) |
| Investigations | 299 | 0.62 ( 0.55 - 0.7 ) | 0.67 ( 55.6 ) | 0.69 ( 0.62 ) | -0.53 ( -0.71 ) |
| Nervous system disorders | 263 | 1.1 ( 0.96 - 1.26 ) | 1.09 ( 1.84 ) | 1.08 ( 0.96 ) | 0.11 ( -0.09 ) |
| Musculoskeletal and connective tissue disorders | 255 | 1.54 ( 1.34 - 1.78 ) | 1.49 ( 37.05 ) | 1.41 ( 1.25 ) | 0.5 ( 0.3 ) |
| Skin and subcutaneous tissue disorders | 207 | 1.51 ( 1.29 - 1.76 ) | 1.47 ( 27.77 ) | 1.4 ( 1.23 ) | 0.48 ( 0.26 ) |
| Injury, poisoning and procedural complications | 128 | 1.28 ( 1.06 - 1.55 ) | 1.27 ( 6.53 ) | 1.23 ( 1.05 ) | 0.3 ( 0.03 ) |
| Gastrointestinal disorders | 125 | 1.24 ( 1.02 - 1.5 ) | 1.23 ( 4.72 ) | 1.2 ( 1.02 ) | 0.26 ( -0.02 ) |
| Respiratory, thoracic and mediastinal disorders | 114 | 1 ( 0.82 - 1.22 ) | 1 ( 0 ) | 1 ( 0.84 ) | 0 ( -0.29 ) |
| Psychiatric disorders | 56 | 1.33 ( 1 - 1.77 ) | 1.32 ( 3.86 ) | 1.28 ( 1.01 ) | 0.35 ( -0.06 ) |
| Surgical and medical procedures | 39 | 0.77 ( 0.56 - 1.08 ) | 0.78 ( 2.3 ) | 0.8 ( 0.6 ) | -0.33 ( -0.81 ) |
| Infections and infestations | 32 | 0.2 ( 0.14 - 0.29 ) | 0.21 ( 95.06 ) | 0.23 ( 0.17 ) | -2.09 ( -2.6 ) |
| Eye disorders | 29 | 1.1 ( 0.75 - 1.63 ) | 1.1 ( 0.25 ) | 1.09 ( 0.79 ) | 0.12 ( -0.44 ) |
| Cardiac disorders | 19 | 0.37 ( 0.24 - 0.59 ) | 0.38 ( 18.83 ) | 0.41 ( 0.28 ) | -1.3 ( -1.96 ) |
| Metabolism and nutrition disorders | 17 | 0.67 ( 0.41 - 1.11 ) | 0.67 ( 2.48 ) | 0.7 ( 0.46 ) | -0.51 ( -1.22 ) |
| Vascular disorders | 13 | 0.49 ( 0.28 - 0.87 ) | 0.5 ( 6.31 ) | 0.53 ( 0.33 ) | -0.93 ( -1.72 ) |
| Ear and labyrinth disorders | 12 | 0.75 ( 0.41 - 1.35 ) | 0.75 ( 0.94 ) | 0.77 ( 0.47 ) | -0.38 ( -1.22 ) |
| Renal and urinary disorders | 7 | 0.44 ( 0.21 - 0.95 ) | 0.44 ( 4.68 ) | 0.47 ( 0.25 ) | -1.08 ( -2.13 ) |
| Hepatobiliary disorders | 5 | 1.19 ( 0.47 - 3.05 ) | 1.19 ( 0.13 ) | 1.17 ( 0.53 ) | 0.22 ( -1.04 ) |
| Immune system disorders | 5 | 0.54 ( 0.22 - 1.33 ) | 0.54 ( 1.84 ) | 0.57 ( 0.27 ) | -0.81 ( -2.04 ) |
| Blood and lymphatic system disorders | 5 | 0.4 ( 0.16 - 0.99 ) | 0.4 ( 4.18 ) | 0.43 ( 0.2 ) | -1.21 ( -2.42 ) |
| Reproductive system and breast disorders | 3 | 0.97 ( 0.29 - 3.22 ) | 0.97 ( 0 ) | 0.97 ( 0.36 ) | -0.04 ( -1.58 ) |
| Endocrine disorders | 3 | 1.74 ( 0.5 - 6.05 ) | 1.74 ( 0.77 ) | 1.61 ( 0.57 ) | 0.68 ( -0.91 ) |
| Congenital, familial and genetic disorders | 1 | 8.1 ( 0.51 - 129.59 ) | 8.1 ( 3.11 ) | 4.55 ( 0.45 ) | 2.19 ( -0.45 ) |
| **RSV + COVID-19 + Influenza Vaccines** | | | | | |
| General disorders and administration site conditions | 222 | 1.24 ( 1.06 - 1.46 ) | 1.17 ( 6.91 ) | 1.16 ( 1.01 ) | 0.21 ( -0.01 ) |
| Nervous system disorders | 88 | 1.21 ( 0.96 - 1.51 ) | 1.18 ( 2.62 ) | 1.17 ( 0.97 ) | 0.23 ( -0.1 ) |
| Investigations | 83 | 0.57 ( 0.45 - 0.72 ) | 0.62 ( 23.29 ) | 0.63 ( 0.52 ) | -0.67 ( -1.01 ) |
| Musculoskeletal and connective tissue disorders | 75 | 1.41 ( 1.1 - 1.8 ) | 1.37 ( 7.67 ) | 1.35 ( 1.1 ) | 0.43 ( 0.08 ) |
| Skin and subcutaneous tissue disorders | 60 | 1.36 ( 1.04 - 1.78 ) | 1.33 ( 5.06 ) | 1.32 ( 1.05 ) | 0.4 ( 0.01 ) |
| Injury, poisoning and procedural complications | 48 | 1.57 ( 1.16 - 2.12 ) | 1.53 ( 8.77 ) | 1.5 ( 1.17 ) | 0.59 ( 0.15 ) |
| Gastrointestinal disorders | 38 | 1.2 ( 0.86 - 1.68 ) | 1.19 ( 1.18 ) | 1.18 ( 0.9 ) | 0.24 ( -0.24 ) |
| Respiratory, thoracic and mediastinal disorders | 34 | 0.97 ( 0.68 - 1.37 ) | 0.97 ( 0.04 ) | 0.97 ( 0.72 ) | -0.05 ( -0.55 ) |
| Psychiatric disorders | 19 | 1.44 ( 0.91 - 2.3 ) | 1.43 ( 2.4 ) | 1.41 ( 0.95 ) | 0.5 ( -0.17 ) |
| Infections and infestations | 10 | 0.22 ( 0.12 - 0.42 ) | 0.23 ( 26.6 ) | 0.24 ( 0.14 ) | -2.06 ( -2.94 ) |
| Surgical and medical procedures | 9 | 0.59 ( 0.3 - 1.14 ) | 0.59 ( 2.55 ) | 0.6 ( 0.34 ) | -0.74 ( -1.67 ) |
| Eye disorders | 8 | 0.98 ( 0.48 - 1.99 ) | 0.98 ( 0 ) | 0.98 ( 0.54 ) | -0.03 ( -1.01 ) |
| Metabolism and nutrition disorders | 6 | 0.8 ( 0.35 - 1.8 ) | 0.8 ( 0.3 ) | 0.8 ( 0.41 ) | -0.31 ( -1.43 ) |
| Vascular disorders | 5 | 0.65 ( 0.27 - 1.58 ) | 0.65 ( 0.93 ) | 0.66 ( 0.31 ) | -0.6 ( -1.8 ) |
| Renal and urinary disorders | 4 | 0.87 ( 0.32 - 2.37 ) | 0.87 ( 0.07 ) | 0.88 ( 0.38 ) | -0.19 ( -1.51 ) |
| Ear and labyrinth disorders | 4 | 0.83 ( 0.31 - 2.25 ) | 0.83 ( 0.14 ) | 0.83 ( 0.36 ) | -0.26 ( -1.58 ) |
| Hepatobiliary disorders | 3 | 2.39 ( 0.73 - 7.79 ) | 2.39 ( 2.23 ) | 2.28 ( 0.85 ) | 1.19 ( -0.33 ) |
| Cardiac disorders | 2 | 0.13 ( 0.03 - 0.53 ) | 0.14 ( 11.21 ) | 0.14 ( 0.04 ) | -2.84 ( -4.52 ) |
| Blood and lymphatic system disorders | 2 | 0.55 ( 0.14 - 2.25 ) | 0.56 ( 0.7 ) | 0.56 ( 0.17 ) | -0.82 ( -2.52 ) |
| Reproductive system and breast disorders | 1 | 1.06 ( 0.14 - 7.82 ) | 1.06 ( 0 ) | 1.06 ( 0.2 ) | 0.08 ( -2.03 ) |
| Immune system disorders | 1 | 0.36 ( 0.05 - 2.6 ) | 0.36 ( 1.11 ) | 0.37 ( 0.07 ) | -1.43 ( -3.5 ) |

**Table S3:** PT-level distribution and signal strength of reported adverse events with positive signals across the three co-administration vaccination groups.

| PT | N | ROR(95%Cl) | PRR(X^2^) | EBGM(EBGM05) | IC(IC025) |
| --- | --- | --- | --- | --- | --- |
| **RSV + COVID-19 Vaccines** | | | | | |
| Headache | 52 | 1.68 ( 1.25 - 2.26 ) | 1.66 ( 12.13 ) | 1.57 ( 1.23 ) | 0.65 ( 0.23 ) |
| Fatigue | 51 | 1.52 ( 1.13 - 2.05 ) | 1.51 ( 7.77 ) | 1.44 ( 1.13 ) | 0.53 ( 0.1 ) |
| Pain in Extremity | 50 | 1.45 ( 1.07 - 1.95 ) | 1.43 ( 5.91 ) | 1.38 ( 1.08 ) | 0.47 ( 0.04 ) |
| Asthenia | 39 | 2 ( 1.42 - 2.82 ) | 1.98 ( 16.16 ) | 1.83 ( 1.37 ) | 0.87 ( 0.38 ) |
| Dizziness | 37 | 1.46 ( 1.03 - 2.07 ) | 1.45 ( 4.7 ) | 1.4 ( 1.05 ) | 0.49 ( -0.01 ) |
| Injection Site Swelling | 29 | 1.71 ( 1.15 - 2.53 ) | 1.69 ( 7.21 ) | 1.6 ( 1.15 ) | 0.68 ( 0.11 ) |
| Vomiting | 23 | 1.75 ( 1.12 - 2.72 ) | 1.74 ( 6.26 ) | 1.64 ( 1.13 ) | 0.71 ( 0.08 ) |
| Mobility Decreased | 22 | 1.94 ( 1.23 - 3.07 ) | 1.93 ( 8.47 ) | 1.79 ( 1.22 ) | 0.84 ( 0.19 ) |
| Influenza Like Illness | 19 | 3.76 ( 2.23 - 6.35 ) | 3.73 ( 28.37 ) | 3.03 ( 1.96 ) | 1.6 ( 0.87 ) |
| Muscular Weakness | 18 | 1.96 ( 1.18 - 3.24 ) | 1.95 ( 7.05 ) | 1.8 ( 1.18 ) | 0.85 ( 0.13 ) |
| Sleep Disorder | 17 | 2.76 ( 1.62 - 4.71 ) | 2.74 ( 15.07 ) | 2.39 ( 1.53 ) | 1.26 ( 0.51 ) |
| Gait Disturbance | 17 | 2.1 ( 1.25 - 3.53 ) | 2.09 ( 8.12 ) | 1.91 ( 1.24 ) | 0.94 ( 0.2 ) |
| Tremor | 16 | 1.89 ( 1.11 - 3.22 ) | 1.88 ( 5.64 ) | 1.75 ( 1.12 ) | 0.81 ( 0.05 ) |
| Hyperhidrosis | 16 | 2.6 ( 1.5 - 4.49 ) | 2.58 ( 12.56 ) | 2.28 ( 1.44 ) | 1.19 ( 0.42 ) |
| Vertigo | 11 | 3.32 ( 1.69 - 6.53 ) | 3.3 ( 13.56 ) | 2.76 ( 1.57 ) | 1.47 ( 0.53 ) |
| Tenderness | 11 | 3.23 ( 1.64 - 6.34 ) | 3.21 ( 12.96 ) | 2.71 ( 1.54 ) | 1.44 ( 0.51 ) |
| Gait Inability | 10 | 3.29 ( 1.62 - 6.68 ) | 3.28 ( 12.16 ) | 2.75 ( 1.52 ) | 1.46 ( 0.48 ) |
| Injection Site Rash | 10 | 2.13 ( 1.08 - 4.19 ) | 2.12 ( 4.96 ) | 1.94 ( 1.1 ) | 0.95 ( 0.01 ) |
| Guillain-Barre Syndrome | 8 | 2.71 ( 1.25 - 5.89 ) | 2.7 ( 6.88 ) | 2.36 ( 1.23 ) | 1.24 ( 0.18 ) |
| Muscle Spasms | 7 | 2.62 ( 1.14 - 5.98 ) | 2.61 ( 5.61 ) | 2.3 ( 1.15 ) | 1.2 ( 0.07 ) |
| Aphasia | 7 | 5.06 ( 2.06 - 12.43 ) | 5.04 ( 15.49 ) | 3.76 ( 1.77 ) | 1.91 ( 0.72 ) |
| Injection Site Mass | 5 | 3.38 ( 1.24 - 9.25 ) | 3.38 ( 6.38 ) | 2.81 ( 1.21 ) | 1.49 ( 0.16 ) |
| Induration | 4 | 6.19 ( 1.81 - 21.16 ) | 6.18 ( 11.05 ) | 4.29 ( 1.54 ) | 2.1 ( 0.57 ) |
| Urinary Incontinence | 4 | 3.33 ( 1.09 - 10.23 ) | 3.33 ( 4.98 ) | 2.78 ( 1.09 ) | 1.47 ( 0.01 ) |
| Feeding Disorder | 4 | 3.33 ( 1.09 - 10.23 ) | 3.33 ( 4.98 ) | 2.78 ( 1.09 ) | 1.47 ( 0.01 ) |
| Csf White Blood Cell Count Negative | 3 | 10.83 ( 2.18 - 53.68 ) | 10.81 ( 13.36 ) | 5.9 ( 1.55 ) | 2.56 ( 0.75 ) |
| Hypotonia | 3 | 6.49 ( 1.55 - 27.2 ) | 6.49 ( 8.7 ) | 4.43 ( 1.34 ) | 2.15 ( 0.41 ) |
| Injection Site Irritation | 3 | 4.64 ( 1.2 - 17.95 ) | 4.63 ( 5.98 ) | 3.54 ( 1.14 ) | 1.82 ( 0.14 ) |
| **RSV + Influenza Vaccines** | | | | | |
| Pain | 68 | 1.57 ( 1.21 - 2.05 ) | 1.56 ( 11.55 ) | 1.47 ( 1.18 ) | 0.55 ( 0.17 ) |
| Pain in Extremity | 67 | 1.52 ( 1.17 - 1.98 ) | 1.51 ( 9.84 ) | 1.43 ( 1.14 ) | 0.51 ( 0.13 ) |
| Erythema | 49 | 1.97 ( 1.43 - 2.69 ) | 1.95 ( 18.37 ) | 1.76 ( 1.35 ) | 0.82 ( 0.37 ) |
| Injection Site Pain | 44 | 1.49 ( 1.08 - 2.07 ) | 1.48 ( 5.97 ) | 1.41 ( 1.07 ) | 0.5 ( 0.03 ) |
| Injection Site Swelling | 38 | 1.76 ( 1.24 - 2.51 ) | 1.75 ( 10.12 ) | 1.62 ( 1.2 ) | 0.69 ( 0.19 ) |
| Peripheral Swelling | 36 | 1.85 ( 1.28 - 2.66 ) | 1.83 ( 11.23 ) | 1.68 ( 1.24 ) | 0.75 ( 0.23 ) |
| Vomiting | 31 | 1.87 ( 1.26 - 2.77 ) | 1.86 ( 10.1 ) | 1.7 ( 1.22 ) | 0.76 ( 0.21 ) |
| Injection Site Warmth | 23 | 1.87 ( 1.19 - 2.95 ) | 1.86 ( 7.52 ) | 1.7 ( 1.16 ) | 0.77 ( 0.12 ) |
| Sleep Disorder | 21 | 2.72 ( 1.65 - 4.46 ) | 2.7 ( 16.92 ) | 2.27 ( 1.5 ) | 1.19 ( 0.49 ) |
| Back Pain | 18 | 1.95 ( 1.16 - 3.27 ) | 1.94 ( 6.68 ) | 1.76 ( 1.14 ) | 0.82 ( 0.09 ) |
| Contusion | 17 | 3.01 ( 1.72 - 5.25 ) | 2.99 ( 16.53 ) | 2.46 ( 1.54 ) | 1.3 ( 0.52 ) |
| Feeling Abnormal | 16 | 1.97 ( 1.14 - 3.41 ) | 1.96 ( 6.11 ) | 1.78 ( 1.12 ) | 0.83 ( 0.06 ) |
| Gait Inability | 12 | 3.15 ( 1.61 - 6.13 ) | 3.14 ( 12.61 ) | 2.54 ( 1.45 ) | 1.34 ( 0.43 ) |
| Injection Site Bruising | 11 | 2.98 ( 1.49 - 5.95 ) | 2.97 ( 10.54 ) | 2.44 ( 1.37 ) | 1.29 ( 0.34 ) |
| Abdominal Pain Upper | 8 | 2.6 ( 1.17 - 5.76 ) | 2.59 ( 5.93 ) | 2.21 ( 1.13 ) | 1.14 ( 0.06 ) |
| Injection Site Mass | 8 | 5 ( 2.07 - 12.07 ) | 4.98 ( 15.79 ) | 3.47 ( 1.66 ) | 1.79 ( 0.65 ) |
| Transient Ischaemic Attack | 6 | 3.48 ( 1.34 - 9.06 ) | 3.47 ( 7.4 ) | 2.73 ( 1.23 ) | 1.45 ( 0.19 ) |
| Musculoskeletal Disorder | 5 | 3.38 ( 1.19 - 9.6 ) | 3.37 ( 5.9 ) | 2.68 ( 1.12 ) | 1.42 ( 0.06 ) |
| Injection Site Vesicles | 4 | 6.49 ( 1.74 - 24.18 ) | 6.48 ( 10.3 ) | 4.04 ( 1.35 ) | 2.02 ( 0.43 ) |
| Fear | 4 | 4.06 ( 1.22 - 13.48 ) | 4.05 ( 6.13 ) | 3.03 ( 1.11 ) | 1.6 ( 0.08 ) |
| Swelling of Eyelid | 3 | 4.86 ( 1.16 - 20.37 ) | 4.86 ( 5.75 ) | 3.41 ( 1.03 ) | 1.77 ( 0.04 ) |
| Scab | 3 | 6.08 ( 1.36 - 27.19 ) | 6.07 ( 7.27 ) | 3.9 ( 1.11 ) | 1.96 ( 0.2 ) |
| Oral Mucosal Blistering | 3 | 8.11 ( 1.64 - 40.2 ) | 8.1 ( 9.34 ) | 4.55 ( 1.19 ) | 2.19 ( 0.38 ) |
| Red Blood Cell Sedimentation Rate Increased | 3 | 8.11 ( 1.64 - 40.2 ) | 8.1 ( 9.34 ) | 4.55 ( 1.19 ) | 2.19 ( 0.38 ) |
| Magnetic Resonance Imaging Abnormal | 3 | 4.05 ( 1.01 - 16.22 ) | 4.05 ( 4.6 ) | 3.03 ( 0.95 ) | 1.6 ( -0.11 ) |
| Muscle Swelling | 3 | 24.33 ( 2.53 - 233.99 ) | 24.3 ( 16.76 ) | 6.82 ( 1.03 ) | 2.77 ( 0.83 ) |
| Csf White Blood Cell Count Increased | 3 | 8.11 ( 1.64 - 40.2 ) | 8.1 ( 9.34 ) | 4.55 ( 1.19 ) | 2.19 ( 0.38 ) |
| **RSV + COVID-19 + Influenza Vaccines** | | | | | |
| Pain | 68 | 1.57 ( 1.21 - 2.05 ) | 1.56 ( 11.55 ) | 1.47 ( 1.18 ) | 0.55 ( 0.17 ) |
| Pain in Extremity | 67 | 1.52 ( 1.17 - 1.98 ) | 1.51 ( 9.84 ) | 1.43 ( 1.14 ) | 0.51 ( 0.13 ) |
| Erythema | 49 | 1.97 ( 1.43 - 2.69 ) | 1.95 ( 18.37 ) | 1.76 ( 1.35 ) | 0.82 ( 0.37 ) |
| Injection Site Pain | 44 | 1.49 ( 1.08 - 2.07 ) | 1.48 ( 5.97 ) | 1.41 ( 1.07 ) | 0.5 ( 0.03 ) |
| Injection Site Swelling | 38 | 1.76 ( 1.24 - 2.51 ) | 1.75 ( 10.12 ) | 1.62 ( 1.2 ) | 0.69 ( 0.19 ) |
| Peripheral Swelling | 36 | 1.85 ( 1.28 - 2.66 ) | 1.83 ( 11.23 ) | 1.68 ( 1.24 ) | 0.75 ( 0.23 ) |
| Vomiting | 31 | 1.87 ( 1.26 - 2.77 ) | 1.86 ( 10.1 ) | 1.7 ( 1.22 ) | 0.76 ( 0.21 ) |
| Injection Site Warmth | 23 | 1.87 ( 1.19 - 2.95 ) | 1.86 ( 7.52 ) | 1.7 ( 1.16 ) | 0.77 ( 0.12 ) |
| Sleep Disorder | 21 | 2.72 ( 1.65 - 4.46 ) | 2.7 ( 16.92 ) | 2.27 ( 1.5 ) | 1.19 ( 0.49 ) |
| Back Pain | 18 | 1.95 ( 1.16 - 3.27 ) | 1.94 ( 6.68 ) | 1.76 ( 1.14 ) | 0.82 ( 0.09 ) |
| Contusion | 17 | 3.01 ( 1.72 - 5.25 ) | 2.99 ( 16.53 ) | 2.46 ( 1.54 ) | 1.3 ( 0.52 ) |
| Feeling Abnormal | 16 | 1.97 ( 1.14 - 3.41 ) | 1.96 ( 6.11 ) | 1.78 ( 1.12 ) | 0.83 ( 0.06 ) |
| Gait Inability | 12 | 3.15 ( 1.61 - 6.13 ) | 3.14 ( 12.61 ) | 2.54 ( 1.45 ) | 1.34 ( 0.43 ) |
| Injection Site Bruising | 11 | 2.98 ( 1.49 - 5.95 ) | 2.97 ( 10.54 ) | 2.44 ( 1.37 ) | 1.29 ( 0.34 ) |
| Injection Site Reaction | 11 | 2.03 ( 1.05 - 3.94 ) | 2.02 ( 4.58 ) | 1.82 ( 1.05 ) | 0.86 ( -0.06 ) |
| Immediate Post-Injection Reaction | 9 | 2.44 ( 1.15 - 5.14 ) | 2.43 ( 5.84 ) | 2.1 ( 1.12 ) | 1.07 ( 0.05 ) |
| Abdominal Pain Upper | 8 | 2.6 ( 1.17 - 5.76 ) | 2.59 ( 5.93 ) | 2.21 ( 1.13 ) | 1.14 ( 0.06 ) |
| Injection Site Mass | 8 | 5 ( 2.07 - 12.07 ) | 4.98 ( 15.79 ) | 3.47 ( 1.66 ) | 1.79 ( 0.65 ) |
| Transient Ischaemic Attack | 6 | 3.48 ( 1.34 - 9.06 ) | 3.47 ( 7.4 ) | 2.73 ( 1.23 ) | 1.45 ( 0.19 ) |
| Musculoskeletal Disorder | 5 | 3.38 ( 1.19 - 9.6 ) | 3.37 ( 5.9 ) | 2.68 ( 1.12 ) | 1.42 ( 0.06 ) |
| Injection Site Vesicles | 4 | 6.49 ( 1.74 - 24.18 ) | 6.48 ( 10.3 ) | 4.04 ( 1.35 ) | 2.02 ( 0.43 ) |
| Fear | 4 | 4.06 ( 1.22 - 13.48 ) | 4.05 ( 6.13 ) | 3.03 ( 1.11 ) | 1.6 ( 0.08 ) |
| Swelling of Eyelid | 3 | 4.86 ( 1.16 - 20.37 ) | 4.86 ( 5.75 ) | 3.41 ( 1.03 ) | 1.77 ( 0.04 ) |
| Scab | 3 | 6.08 ( 1.36 - 27.19 ) | 6.07 ( 7.27 ) | 3.9 ( 1.11 ) | 1.96 ( 0.2 ) |
| Oral Mucosal Blistering | 3 | 8.11 ( 1.64 - 40.2 ) | 8.1 ( 9.34 ) | 4.55 ( 1.19 ) | 2.19 ( 0.38 ) |
| Red Blood Cell Sedimentation Rate Increased | 3 | 8.11 ( 1.64 - 40.2 ) | 8.1 ( 9.34 ) | 4.55 ( 1.19 ) | 2.19 ( 0.38 ) |
| Magnetic Resonance Imaging Abnormal | 3 | 4.05 ( 1.01 - 16.22 ) | 4.05 ( 4.6 ) | 3.03 ( 0.95 ) | 1.6 ( -0.11 ) |
| Muscle Swelling | 3 | 24.33 ( 2.53 - 233.99 ) | 24.3 ( 16.76 ) | 6.82 ( 1.03 ) | 2.77 ( 0.83 ) |
| Csf White Blood Cell Count Increased | 3 | 8.11 ( 1.64 - 40.2 ) | 8.1 ( 9.34 ) | 4.55 ( 1.19 ) | 2.19 ( 0.38 ) |

**Table S4:** Distribution and signal strength of adverse events in serious reports across the three co-administration vaccination groups.

| **RSV＋COVID-19 Vaccine** | | | | | |
| --- | --- | --- | --- | --- | --- |
| **PT** | **N(%)** | **ROR(95%Cl)** | **PRR(X^2^)** | **EBGM(EBGM05)** | **IC(IC025)** |
| Asthenia | 9 (2.7%) | 2.15 ( 1.07 - 4.32 ) | 2.12 ( 4.85 ) | 2.01 ( 1.12 ) | 1 ( 0.03 ) |
| Muscular weakness | 9 (2.7%) | 4.81 ( 2.3 - 10.07 ) | 4.71 ( 21.18 ) | 3.97 ( 2.14 ) | 1.99 ( 0.97 ) |
| Guillain-barre syndrome | 7 (2.1%) | 5.16 ( 2.22 - 11.97 ) | 5.07 ( 18.12 ) | 4.21 ( 2.08 ) | 2.07 ( 0.93 ) |
| Pain in extremity | 6 (1.8%) | 3.58 ( 1.49 - 8.62 ) | 3.53 ( 9.22 ) | 3.13 ( 1.5 ) | 1.65 ( 0.45 ) |
| Mobility decreased | 6 (1.8%) | 4.58 ( 1.87 - 11.25 ) | 4.52 ( 13.33 ) | 3.84 ( 1.81 ) | 1.94 ( 0.73 ) |
| Cerebrovascular accident | 6 (1.8%) | 4.24 ( 1.74 - 10.35 ) | 4.19 ( 11.96 ) | 3.61 ( 1.71 ) | 1.85 ( 0.64 ) |
| Gait disturbance | 5 (1.5%) | 3.07 ( 1.19 - 7.94 ) | 3.04 ( 5.92 ) | 2.76 ( 1.24 ) | 1.46 ( 0.18 ) |
| Dyspnoea | 5 (1.5%) | 0.97 ( 0.39 - 2.4 ) | 0.97 ( 0 ) | 0.97 ( 0.46 ) | -0.04 ( -1.26 ) |
| Feeling abnormal | 4 (1.2%) | 6.92 ( 2.19 - 21.85 ) | 6.85 ( 14.69 ) | 5.29 ( 2.02 ) | 2.4 ( 0.92 ) |
| Influenza like illness | 3 (0.9%) | 8.14 ( 2.09 - 31.61 ) | 8.07 ( 13.03 ) | 5.95 ( 1.91 ) | 2.57 ( 0.88 ) |
| Platelet count decreased | 3 (0.9%) | 11.39 ( 2.71 - 47.88 ) | 11.3 ( 17.63 ) | 7.44 ( 2.24 ) | 2.9 ( 1.16 ) |
| Aphasia | 3 (0.9%) | 5.17 ( 1.44 - 18.64 ) | 5.14 ( 7.87 ) | 4.25 ( 1.45 ) | 2.09 ( 0.46 ) |
| Mental status changes | 3 (0.9%) | 4.74 ( 1.33 - 16.89 ) | 4.71 ( 7.03 ) | 3.97 ( 1.37 ) | 1.99 ( 0.37 ) |
| Csf glucose increased | 2 (0.6%) | 7.57 ( 1.46 - 39.18 ) | 7.53 ( 8.1 ) | 5.67 ( 1.43 ) | 2.5 ( 0.54 ) |
| Csf protein increased | 2 (0.6%) | 4.73 ( 1 - 22.37 ) | 4.71 ( 4.68 ) | 3.97 ( 1.08 ) | 1.99 ( 0.1 ) |
| Deep vein thrombosis | 2 (0.6%) | 5.41 ( 1.12 - 26.13 ) | 5.38 ( 5.56 ) | 4.41 ( 1.18 ) | 2.14 ( 0.24 ) |
| Hypotonia | 2 (0.6%) | 37.89 ( 3.43 - 418.96 ) | 37.67 ( 23.81 ) | 13.22 ( 1.77 ) | 3.73 ( 1.52 ) |
| Ascending flaccid paralysis | 2 (0.6%) | 18.94 ( 2.66 - 134.9 ) | 18.84 ( 16.9 ) | 9.92 ( 1.92 ) | 3.31 ( 1.2 ) |
| Amnesia | 2 (0.6%) | 6.31 ( 1.27 - 31.38 ) | 6.28 ( 6.67 ) | 4.96 ( 1.3 ) | 2.31 ( 0.38 ) |
| Hypoaesthesia | 2 (0.6%) | 1.57 ( 0.37 - 6.68 ) | 1.57 ( 0.38 ) | 1.53 ( 0.45 ) | 0.61 ( -1.15 ) |
| Musculoskeletal disorder | 2 (0.6%) | 4.73 ( 1 - 22.37 ) | 4.71 ( 4.68 ) | 3.97 ( 1.08 ) | 1.99 ( 0.1 ) |
| Hemiplegia | 2 (0.6%) | 37.89 ( 3.43 - 418.96 ) | 37.67 ( 23.81 ) | 13.22 ( 1.77 ) | 3.73 ( 1.52 ) |
| Magnetic resonance imaging head abnormal | 2 (0.6%) | 3.44 ( 0.76 - 15.58 ) | 3.42 ( 2.91 ) | 3.05 ( 0.86 ) | 1.61 ( -0.23 ) |
| Pulmonary embolism | 2 (0.6%) | 2.1 ( 0.49 - 9.09 ) | 2.09 ( 1.03 ) | 1.98 ( 0.58 ) | 0.99 ( -0.8 ) |
| Vertigo | 2 (0.6%) | 7.57 ( 1.46 - 39.18 ) | 7.53 ( 8.1 ) | 5.67 ( 1.43 ) | 2.5 ( 0.54 ) |
| White blood cell count increased | 2 (0.6%) | 3.15 ( 0.7 - 14.14 ) | 3.14 ( 2.5 ) | 2.83 ( 0.81 ) | 1.5 ( -0.33 ) |
| Arthralgia | 2 (0.6%) | 2.1 ( 0.49 - 9.09 ) | 2.09 ( 1.03 ) | 1.98 ( 0.58 ) | 0.99 ( -0.8 ) |
| Neck pain | 2 (0.6%) | 4.73 ( 1 - 22.37 ) | 4.71 ( 4.68 ) | 3.97 ( 1.08 ) | 1.99 ( 0.1 ) |
| Delusion | 2 (0.6%) | 12.63 ( 2.1 - 75.82 ) | 12.56 ( 12.77 ) | 7.93 ( 1.77 ) | 2.99 ( 0.94 ) |
| Sepsis | 2 (0.6%) | 1.51 ( 0.36 - 6.4 ) | 1.51 ( 0.32 ) | 1.47 ( 0.44 ) | 0.56 ( -1.2 ) |
| Dysgraphia | 2 (0.6%) | 7.57 ( 1.46 - 39.18 ) | 7.53 ( 8.1 ) | 5.67 ( 1.43 ) | 2.5 ( 0.54 ) |
| Fine motor skill dysfunction | 2 (0.6%) | 12.63 ( 2.1 - 75.82 ) | 12.56 ( 12.77 ) | 7.93 ( 1.77 ) | 2.99 ( 0.94 ) |
| Lumbar puncture abnormal | 1 (0.3%) | 9.44 ( 0.85 - 104.41 ) | 9.42 ( 5.02 ) | 6.61 ( 0.89 ) | 2.73 ( 0.22 ) |
| Chills | 1 (0.3%) | 0.86 ( 0.12 - 6.37 ) | 0.86 ( 0.02 ) | 0.86 ( 0.16 ) | -0.21 ( -2.34 ) |
| Tremor | 1 (0.3%) | 2.36 ( 0.29 - 18.91 ) | 2.35 ( 0.69 ) | 2.2 ( 0.39 ) | 1.14 ( -1.1 ) |
| Neurological symptom | 1 (0.3%) | 2.7 ( 0.33 - 21.98 ) | 2.69 ( 0.93 ) | 2.48 ( 0.43 ) | 1.31 ( -0.95 ) |
| Cardiac arrest | 1 (0.3%) | 1.57 ( 0.2 - 12.12 ) | 1.57 ( 0.19 ) | 1.53 ( 0.28 ) | 0.61 ( -1.58 ) |
| Posture abnormal | 1 (0.3%) | 9.44 ( 0.85 - 104.41 ) | 9.42 ( 5.02 ) | 6.61 ( 0.89 ) | 2.73 ( 0.22 ) |
| Electrocardiogram abnormal | 1 (0.3%) | 1.57 ( 0.2 - 12.12 ) | 1.57 ( 0.19 ) | 1.53 ( 0.28 ) | 0.61 ( -1.58 ) |
| Coronary artery occlusion | 1 (0.3%) | 18.89 ( 1.18 - 302.67 ) | 18.84 ( 8.45 ) | 9.92 ( 0.97 ) | 3.31 ( 0.67 ) |
| Echocardiogram abnormal | 1 (0.3%) | 1.45 ( 0.19 - 11.12 ) | 1.45 ( 0.13 ) | 1.42 ( 0.26 ) | 0.5 ( -1.67 ) |
| Myocarditis | 1 (0.3%) | 1.26 ( 0.17 - 9.54 ) | 1.26 ( 0.05 ) | 1.24 ( 0.23 ) | 0.31 ( -1.85 ) |
| Hyperhidrosis | 1 (0.3%) | 2.1 ( 0.26 - 16.59 ) | 2.09 ( 0.51 ) | 1.98 ( 0.35 ) | 0.99 ( -1.23 ) |
| Somnolence | 1 (0.3%) | 2.7 ( 0.33 - 21.98 ) | 2.69 ( 0.93 ) | 2.48 ( 0.43 ) | 1.31 ( -0.95 ) |
| Hypotension | 1 (0.3%) | 1.05 ( 0.14 - 7.86 ) | 1.05 ( 0 ) | 1.04 ( 0.19 ) | 0.06 ( -2.08 ) |
| Troponin increased | 1 (0.3%) | 1.45 ( 0.19 - 11.12 ) | 1.45 ( 0.13 ) | 1.42 ( 0.26 ) | 0.5 ( -1.67 ) |
| Feeding disorder | 1 (0.3%) | 4.72 ( 0.53 - 42.35 ) | 4.71 ( 2.34 ) | 3.97 ( 0.63 ) | 1.99 ( -0.37 ) |
| Thrombocytopenia | 1 (0.3%) | 3.78 ( 0.44 - 32.41 ) | 3.77 ( 1.7 ) | 3.31 ( 0.55 ) | 1.73 ( -0.59 ) |
| Balance disorder | 1 (0.3%) | 1.35 ( 0.18 - 10.27 ) | 1.35 ( 0.08 ) | 1.32 ( 0.24 ) | 0.4 ( -1.77 ) |
| Fluid retention | 1 (0.3%) | 18.89 ( 1.18 - 302.67 ) | 18.84 ( 8.45 ) | 9.92 ( 0.97 ) | 3.31 ( 0.67 ) |
| Dysarthria | 1 (0.3%) | 1.71 ( 0.22 - 13.32 ) | 1.71 ( 0.27 ) | 1.65 ( 0.3 ) | 0.73 ( -1.47 ) |
| Transient ischaemic attack | 1 (0.3%) | 1.18 ( 0.16 - 8.91 ) | 1.18 ( 0.03 ) | 1.17 ( 0.21 ) | 0.22 ( -1.93 ) |
| Csf red blood cell count positive | 1 (0.3%) | 4.72 ( 0.53 - 42.35 ) | 4.71 ( 2.34 ) | 3.97 ( 0.63 ) | 1.99 ( -0.37 ) |
| Electromyogram abnormal | 1 (0.3%) | 9.44 ( 0.85 - 104.41 ) | 9.42 ( 5.02 ) | 6.61 ( 0.89 ) | 2.73 ( 0.22 ) |
| Pallor | 1 (0.3%) | 9.44 ( 0.85 - 104.41 ) | 9.42 ( 5.02 ) | 6.61 ( 0.89 ) | 2.73 ( 0.22 ) |
| Mechanical ventilation | 1 (0.3%) | 2.1 ( 0.26 - 16.59 ) | 2.09 ( 0.51 ) | 1.98 ( 0.35 ) | 0.99 ( -1.23 ) |
| Areflexia | 1 (0.3%) | 18.89 ( 1.18 - 302.67 ) | 18.84 ( 8.45 ) | 9.92 ( 0.97 ) | 3.31 ( 0.67 ) |
| Plasmapheresis | 1 (0.3%) | 4.72 ( 0.53 - 42.35 ) | 4.71 ( 2.34 ) | 3.97 ( 0.63 ) | 1.99 ( -0.37 ) |
| Cranial nerve disorder | 1 (0.3%) | 9.44 ( 0.85 - 104.41 ) | 9.42 ( 5.02 ) | 6.61 ( 0.89 ) | 2.73 ( 0.22 ) |
| Chest discomfort | 1 (0.3%) | 1.45 ( 0.19 - 11.12 ) | 1.45 ( 0.13 ) | 1.42 ( 0.26 ) | 0.5 ( -1.67 ) |
| Heart rate decreased | 1 (0.3%) | 3.15 ( 0.38 - 26.21 ) | 3.14 ( 1.25 ) | 2.83 ( 0.48 ) | 1.5 ( -0.78 ) |
| Hepatitis acute | 1 (0.3%) | 6.29 ( 0.65 - 60.68 ) | 6.28 ( 3.33 ) | 4.96 ( 0.74 ) | 2.31 ( -0.11 ) |
| Chest pain | 1 (0.3%) | 0.78 ( 0.11 - 5.81 ) | 0.78 ( 0.06 ) | 0.79 ( 0.15 ) | -0.33 ( -2.46 ) |
| Hypertension | 1 (0.3%) | 0.99 ( 0.13 - 7.43 ) | 0.99 ( 0 ) | 0.99 ( 0.18 ) | -0.01 ( -2.15 ) |
| Cognitive disorder | 1 (0.3%) | 2.7 ( 0.33 - 21.98 ) | 2.69 ( 0.93 ) | 2.48 ( 0.43 ) | 1.31 ( -0.95 ) |
| Incoherent | 1 (0.3%) | 2.36 ( 0.29 - 18.91 ) | 2.35 ( 0.69 ) | 2.2 ( 0.39 ) | 1.14 ( -1.1 ) |
| Computerised tomogram abnormal | 1 (0.3%) | 6.29 ( 0.65 - 60.68 ) | 6.28 ( 3.33 ) | 4.96 ( 0.74 ) | 2.31 ( -0.11 ) |
| Magnetic resonance imaging abnormal | 1 (0.3%) | 6.29 ( 0.65 - 60.68 ) | 6.28 ( 3.33 ) | 4.96 ( 0.74 ) | 2.31 ( -0.11 ) |
| Speech disorder | 1 (0.3%) | 2.1 ( 0.26 - 16.59 ) | 2.09 ( 0.51 ) | 1.98 ( 0.35 ) | 0.99 ( -1.23 ) |
| Dehydration | 1 (0.3%) | 2.36 ( 0.29 - 18.91 ) | 2.35 ( 0.69 ) | 2.2 ( 0.39 ) | 1.14 ( -1.1 ) |
| Hypophagia | 1 (0.3%) | 3.15 ( 0.38 - 26.21 ) | 3.14 ( 1.25 ) | 2.83 ( 0.48 ) | 1.5 ( -0.78 ) |
| Lethargy | 1 (0.3%) | 1.71 ( 0.22 - 13.32 ) | 1.71 ( 0.27 ) | 1.65 ( 0.3 ) | 0.73 ( -1.47 ) |
| Blindness | 1 (0.3%) | 9.44 ( 0.85 - 104.41 ) | 9.42 ( 5.02 ) | 6.61 ( 0.89 ) | 2.73 ( 0.22 ) |
| Retinal artery occlusion | 1 (0.3%) | 18.89 ( 1.18 - 302.67 ) | 18.84 ( 8.45 ) | 9.92 ( 0.97 ) | 3.31 ( 0.67 ) |
| Retinal vein occlusion | 1 (0.3%) | 18.89 ( 1.18 - 302.67 ) | 18.84 ( 8.45 ) | 9.92 ( 0.97 ) | 3.31 ( 0.67 ) |
| Cerebral haemorrhage | 1 (0.3%) | 2.36 ( 0.29 - 18.91 ) | 2.35 ( 0.69 ) | 2.2 ( 0.39 ) | 1.14 ( -1.1 ) |
| Upper respiratory tract infection | 1 (0.3%) | 6.29 ( 0.65 - 60.68 ) | 6.28 ( 3.33 ) | 4.96 ( 0.74 ) | 2.31 ( -0.11 ) |
| X-ray limb abnormal | 1 (0.3%) | 9.44 ( 0.85 - 104.41 ) | 9.42 ( 5.02 ) | 6.61 ( 0.89 ) | 2.73 ( 0.22 ) |
| Red blood cell sedimentation rate increased | 1 (0.3%) | 9.44 ( 0.85 - 104.41 ) | 9.42 ( 5.02 ) | 6.61 ( 0.89 ) | 2.73 ( 0.22 ) |
| Cerebral artery thrombosis | 1 (0.3%) | 18.89 ( 1.18 - 302.67 ) | 18.84 ( 8.45 ) | 9.92 ( 0.97 ) | 3.31 ( 0.67 ) |
| Hemiparesis | 1 (0.3%) | 2.36 ( 0.29 - 18.91 ) | 2.35 ( 0.69 ) | 2.2 ( 0.39 ) | 1.14 ( -1.1 ) |
| Pruritus | 1 (0.3%) | 3.78 ( 0.44 - 32.41 ) | 3.77 ( 1.7 ) | 3.31 ( 0.55 ) | 1.73 ( -0.59 ) |
| Rash erythematous | 1 (0.3%) | 18.89 ( 1.18 - 302.67 ) | 18.84 ( 8.45 ) | 9.92 ( 0.97 ) | 3.31 ( 0.67 ) |
| Sleep disorder | 1 (0.3%) | 1.89 ( 0.24 - 14.78 ) | 1.88 ( 0.38 ) | 1.8 ( 0.32 ) | 0.85 ( -1.36 ) |
| Respiratory failure | 1 (0.3%) | 2.1 ( 0.26 - 16.59 ) | 2.09 ( 0.51 ) | 1.98 ( 0.35 ) | 0.99 ( -1.23 ) |
| Scan with contrast abnormal | 1 (0.3%) | 3.15 ( 0.38 - 26.21 ) | 3.14 ( 1.25 ) | 2.83 ( 0.48 ) | 1.5 ( -0.78 ) |
| Spinal stenosis | 1 (0.3%) | 18.89 ( 1.18 - 302.67 ) | 18.84 ( 8.45 ) | 9.92 ( 0.97 ) | 3.31 ( 0.67 ) |
| Magnetic resonance imaging spinal abnormal | 1 (0.3%) | 3.78 ( 0.44 - 32.41 ) | 3.77 ( 1.7 ) | 3.31 ( 0.55 ) | 1.73 ( -0.59 ) |
| White matter lesion | 1 (0.3%) | 18.89 ( 1.18 - 302.67 ) | 18.84 ( 8.45 ) | 9.92 ( 0.97 ) | 3.31 ( 0.67 ) |
| Computerised tomogram thorax abnormal | 1 (0.3%) | 1.05 ( 0.14 - 7.86 ) | 1.05 ( 0 ) | 1.04 ( 0.19 ) | 0.06 ( -2.08 ) |
| Head injury | 1 (0.3%) | 2.7 ( 0.33 - 21.98 ) | 2.69 ( 0.93 ) | 2.48 ( 0.43 ) | 1.31 ( -0.95 ) |
| Loss of consciousness | 1 (0.3%) | 1.26 ( 0.17 - 9.54 ) | 1.26 ( 0.05 ) | 1.24 ( 0.23 ) | 0.31 ( -1.85 ) |
| Csf white blood cell count increased | 1 (0.3%) | 3.78 ( 0.44 - 32.41 ) | 3.77 ( 1.7 ) | 3.31 ( 0.55 ) | 1.73 ( -0.59 ) |
| Encephalitis | 1 (0.3%) | 6.29 ( 0.65 - 60.68 ) | 6.28 ( 3.33 ) | 4.96 ( 0.74 ) | 2.31 ( -0.11 ) |
| Pneumonia | 1 (0.3%) | 0.61 ( 0.08 - 4.46 ) | 0.61 ( 0.25 ) | 0.62 ( 0.12 ) | -0.69 ( -2.8 ) |
| Ultrasound doppler abnormal | 1 (0.3%) | 3.78 ( 0.44 - 32.41 ) | 3.77 ( 1.7 ) | 3.31 ( 0.55 ) | 1.73 ( -0.59 ) |
| Diabetes mellitus | 1 (0.3%) | 9.44 ( 0.85 - 104.41 ) | 9.42 ( 5.02 ) | 6.61 ( 0.89 ) | 2.73 ( 0.22 ) |
| Lymphadenopathy | 1 (0.3%) | 3.78 ( 0.44 - 32.41 ) | 3.77 ( 1.7 ) | 3.31 ( 0.55 ) | 1.73 ( -0.59 ) |
| Deafness neurosensory | 1 (0.3%) | 18.89 ( 1.18 - 302.67 ) | 18.84 ( 8.45 ) | 9.92 ( 0.97 ) | 3.31 ( 0.67 ) |
| Deafness unilateral | 1 (0.3%) | 6.29 ( 0.65 - 60.68 ) | 6.28 ( 3.33 ) | 4.96 ( 0.74 ) | 2.31 ( -0.11 ) |
| Sudden hearing loss | 1 (0.3%) | 18.89 ( 1.18 - 302.67 ) | 18.84 ( 8.45 ) | 9.92 ( 0.97 ) | 3.31 ( 0.67 ) |
| Tinnitus | 1 (0.3%) | 3.78 ( 0.44 - 32.41 ) | 3.77 ( 1.7 ) | 3.31 ( 0.55 ) | 1.73 ( -0.59 ) |
| Injection site pain | 1 (0.3%) | 1.71 ( 0.22 - 13.32 ) | 1.71 ( 0.27 ) | 1.65 ( 0.3 ) | 0.73 ( -1.47 ) |
| Vision blurred | 1 (0.3%) | 1.89 ( 0.24 - 14.78 ) | 1.88 ( 0.38 ) | 1.8 ( 0.32 ) | 0.85 ( -1.36 ) |
| Periarthritis | 1 (0.3%) | 18.89 ( 1.18 - 302.67 ) | 18.84 ( 8.45 ) | 9.92 ( 0.97 ) | 3.31 ( 0.67 ) |
| Injection site swelling | 1 (0.3%) | 9.44 ( 0.85 - 104.41 ) | 9.42 ( 5.02 ) | 6.61 ( 0.89 ) | 2.73 ( 0.22 ) |
| Peripheral swelling | 1 (0.3%) | 1.26 ( 0.17 - 9.54 ) | 1.26 ( 0.05 ) | 1.24 ( 0.23 ) | 0.31 ( -1.85 ) |
| Pain | 1 (0.3%) | 0.52 ( 0.07 - 3.82 ) | 0.52 ( 0.43 ) | 0.54 ( 0.1 ) | -0.9 ( -3 ) |
| Erythema | 1 (0.3%) | 2.7 ( 0.33 - 21.98 ) | 2.69 ( 0.93 ) | 2.48 ( 0.43 ) | 1.31 ( -0.95 ) |
| Injected limb mobility decreased | 1 (0.3%) | 4.72 ( 0.53 - 42.35 ) | 4.71 ( 2.34 ) | 3.97 ( 0.63 ) | 1.99 ( -0.37 ) |
| Skin warm | 1 (0.3%) | 18.89 ( 1.18 - 302.67 ) | 18.84 ( 8.45 ) | 9.92 ( 0.97 ) | 3.31 ( 0.67 ) |
| Movement disorder | 1 (0.3%) | 18.89 ( 1.18 - 302.67 ) | 18.84 ( 8.45 ) | 9.92 ( 0.97 ) | 3.31 ( 0.67 ) |
| Personality change | 1 (0.3%) | 18.89 ( 1.18 - 302.67 ) | 18.84 ( 8.45 ) | 9.92 ( 0.97 ) | 3.31 ( 0.67 ) |
| Positive airway pressure therapy | 1 (0.3%) | 3.15 ( 0.38 - 26.21 ) | 3.14 ( 1.25 ) | 2.83 ( 0.48 ) | 1.5 ( -0.78 ) |
| Nausea | 1 (0.3%) | 0.46 ( 0.06 - 3.34 ) | 0.46 ( 0.62 ) | 0.47 ( 0.09 ) | -1.08 ( -3.17 ) |
| **RSV + Influenza Vaccines** | | | | | |
| **PT** | **N(%)** | **ROR(95%Cl)** | **PRR(卡方值)** | **EBGM(EBGM05)** | **IC(IC025)** |
| Muscular weakness | 10 (1.8%) | 3.19 ( 1.57 - 6.48 ) | 3.15 ( 11.5 ) | 2.67 ( 1.48 ) | 1.42 ( 0.44 ) |
| Guillain-barre syndrome | 7 (1.3%) | 3 ( 1.29 - 6.93 ) | 2.97 ( 7.24 ) | 2.55 ( 1.26 ) | 1.35 ( 0.21 ) |
| Mobility decreased | 7 (1.3%) | 3.25 ( 1.39 - 7.57 ) | 3.22 ( 8.32 ) | 2.72 ( 1.34 ) | 1.44 ( 0.3 ) |
| Cerebrovascular accident | 6 (1.1%) | 2.47 ( 1.01 - 6 ) | 2.45 ( 4.24 ) | 2.19 ( 1.04 ) | 1.13 ( -0.07 ) |
| Feeling abnormal | 5 (0.9%) | 5.56 ( 1.89 - 16.31 ) | 5.52 ( 12.35 ) | 4.01 ( 1.63 ) | 2 ( 0.62 ) |
| Transient ischaemic attack | 4 (0.7%) | 3.41 ( 1.11 - 10.5 ) | 3.39 ( 5.18 ) | 2.83 ( 1.11 ) | 1.5 ( 0.04 ) |
| Musculoskeletal disorder | 4 (0.7%) | 7.4 ( 2.08 - 26.3 ) | 7.35 ( 13.2 ) | 4.81 ( 1.67 ) | 2.27 ( 0.71 ) |
| Sleep disorder | 4 (0.7%) | 6.34 ( 1.85 - 21.73 ) | 6.3 ( 11.38 ) | 4.37 ( 1.56 ) | 2.13 ( 0.59 ) |
| Mechanical ventilation | 3 (0.5%) | 4.75 ( 1.22 - 18.41 ) | 4.73 ( 6.18 ) | 3.61 ( 1.16 ) | 1.85 ( 0.16 ) |
| Somnolence | 3 (0.5%) | 6.65 ( 1.58 - 27.9 ) | 6.62 ( 8.95 ) | 4.51 ( 1.36 ) | 2.17 ( 0.44 ) |
| Incoherent | 3 (0.5%) | 5.54 ( 1.38 - 22.21 ) | 5.52 ( 7.4 ) | 4.01 ( 1.25 ) | 2 ( 0.29 ) |
| Resuscitation | 3 (0.5%) | 3.69 ( 1 - 13.68 ) | 3.68 ( 4.39 ) | 3.01 ( 1.01 ) | 1.59 ( -0.06 ) |
| Loss of consciousness | 3 (0.5%) | 2.55 ( 0.73 - 8.99 ) | 2.55 ( 2.29 ) | 2.26 ( 0.79 ) | 1.17 ( -0.43 ) |
| Gait inability | 3 (0.5%) | 1.95 ( 0.57 - 6.68 ) | 1.95 ( 1.18 ) | 1.8 ( 0.64 ) | 0.85 ( -0.73 ) |
| Csf white blood cell count increased | 3 (0.5%) | 11.09 ( 2.23 - 55.05 ) | 11.03 ( 13.69 ) | 6.02 ( 1.57 ) | 2.59 ( 0.78 ) |
| Aphasia | 3 (0.5%) | 3.02 ( 0.84 - 10.85 ) | 3.01 ( 3.17 ) | 2.58 ( 0.88 ) | 1.37 ( -0.26 ) |
| Fatigue | 3 (0.5%) | 0.49 ( 0.15 - 1.57 ) | 0.49 ( 1.5 ) | 0.52 ( 0.2 ) | -0.96 ( -2.44 ) |
| Malaise | 3 (0.5%) | 0.79 ( 0.24 - 2.55 ) | 0.79 ( 0.16 ) | 0.8 ( 0.3 ) | -0.32 ( -1.83 ) |
| Myalgia | 3 (0.5%) | 2.37 ( 0.68 - 8.28 ) | 2.36 ( 1.95 ) | 2.12 ( 0.75 ) | 1.09 ( -0.51 ) |
| Mental status changes | 3 (0.5%) | 2.77 ( 0.78 - 9.84 ) | 2.76 ( 2.69 ) | 2.41 ( 0.83 ) | 1.27 ( -0.35 ) |
| Neck pain | 3 (0.5%) | 4.75 ( 1.22 - 18.41 ) | 4.73 ( 6.18 ) | 3.61 ( 1.16 ) | 1.85 ( 0.16 ) |
| Sepsis | 3 (0.5%) | 1.38 ( 0.41 - 4.6 ) | 1.38 ( 0.28 ) | 1.34 ( 0.49 ) | 0.42 ( -1.13 ) |
| Chest x-ray abnormal | 2 (0.4%) | 0.92 ( 0.22 - 3.9 ) | 0.92 ( 0.01 ) | 0.93 ( 0.28 ) | -0.11 ( -1.87 ) |
| Echocardiogram abnormal | 2 (0.4%) | 1.84 ( 0.41 - 8.25 ) | 1.84 ( 0.66 ) | 1.72 ( 0.49 ) | 0.78 ( -1.05 ) |
| Injection site pain | 2 (0.4%) | 2.21 ( 0.48 - 10.11 ) | 2.21 ( 1.1 ) | 2.01 ( 0.56 ) | 1 ( -0.85 ) |
| Chills | 2 (0.4%) | 1.05 ( 0.25 - 4.49 ) | 1.05 ( 0 ) | 1.05 ( 0.31 ) | 0.07 ( -1.71 ) |
| Deafness unilateral | 2 (0.4%) | 11.07 ( 1.56 - 78.72 ) | 11.03 ( 9.13 ) | 6.02 ( 1.16 ) | 2.59 ( 0.48 ) |
| Lumbar puncture | 2 (0.4%) | 5.53 ( 1.01 - 30.27 ) | 5.52 ( 4.93 ) | 4.01 ( 0.97 ) | 2 ( 0.01 ) |
| Back pain | 2 (0.4%) | 0.96 ( 0.23 - 4.08 ) | 0.96 ( 0 ) | 0.96 ( 0.29 ) | -0.06 ( -1.82 ) |
| Unresponsive to stimuli | 2 (0.4%) | 1.7 ( 0.38 - 7.55 ) | 1.7 ( 0.5 ) | 1.6 ( 0.46 ) | 0.68 ( -1.14 ) |
| Chest pain | 2 (0.4%) | 0.96 ( 0.23 - 4.08 ) | 0.96 ( 0 ) | 0.96 ( 0.29 ) | -0.06 ( -1.82 ) |
| Red blood cell sedimentation rate increased | 2 (0.4%) | 22.14 ( 2 - 244.54 ) | 22.06 ( 13.41 ) | 8.02 ( 1.07 ) | 3 ( 0.8 ) |
| Dehydration | 2 (0.4%) | 3.16 ( 0.65 - 15.25 ) | 3.15 ( 2.29 ) | 2.67 ( 0.72 ) | 1.42 ( -0.48 ) |
| Respiratory distress | 2 (0.4%) | 2.46 ( 0.53 - 11.4 ) | 2.45 ( 1.41 ) | 2.19 ( 0.61 ) | 1.13 ( -0.74 ) |
| Headache | 2 (0.4%) | 0.54 ( 0.13 - 2.22 ) | 0.54 ( 0.76 ) | 0.56 ( 0.17 ) | -0.84 ( -2.56 ) |
| Hypoaesthesia | 2 (0.4%) | 0.92 ( 0.22 - 3.9 ) | 0.92 ( 0.01 ) | 0.93 ( 0.28 ) | -0.11 ( -1.87 ) |
| Visual impairment | 2 (0.4%) | 5.53 ( 1.01 - 30.27 ) | 5.52 ( 4.93 ) | 4.01 ( 0.97 ) | 2 ( 0.01 ) |
| Scan with contrast abnormal | 2 (0.4%) | 4.42 ( 0.86 - 22.86 ) | 4.41 ( 3.77 ) | 3.44 ( 0.87 ) | 1.78 ( -0.17 ) |
| Heart rate decreased | 2 (0.4%) | 4.42 ( 0.86 - 22.86 ) | 4.41 ( 3.77 ) | 3.44 ( 0.87 ) | 1.78 ( -0.17 ) |
| Hypertension | 2 (0.4%) | 1.23 ( 0.28 - 5.3 ) | 1.23 ( 0.07 ) | 1.2 ( 0.35 ) | 0.27 ( -1.52 ) |
| Computerised tomogram abnormal | 2 (0.4%) | 11.07 ( 1.56 - 78.72 ) | 11.03 ( 9.13 ) | 6.02 ( 1.16 ) | 2.59 ( 0.48 ) |
| Magnetic resonance imaging abnormal | 2 (0.4%) | 11.07 ( 1.56 - 78.72 ) | 11.03 ( 9.13 ) | 6.02 ( 1.16 ) | 2.59 ( 0.48 ) |
| Confusional state | 2 (0.4%) | 0.82 ( 0.19 - 3.44 ) | 0.82 ( 0.08 ) | 0.83 ( 0.25 ) | -0.27 ( -2.02 ) |
| Csf glucose increased | 2 (0.4%) | 4.42 ( 0.86 - 22.86 ) | 4.41 ( 3.77 ) | 3.44 ( 0.87 ) | 1.78 ( -0.17 ) |
| Diarrhoea | 2 (0.4%) | 0.85 ( 0.2 - 3.58 ) | 0.85 ( 0.05 ) | 0.86 ( 0.26 ) | -0.22 ( -1.97 ) |
| Csf protein increased | 2 (0.4%) | 2.76 ( 0.59 - 13.05 ) | 2.76 ( 1.8 ) | 2.41 ( 0.66 ) | 1.27 ( -0.62 ) |
| Dizziness | 2 (0.4%) | 0.58 ( 0.14 - 2.41 ) | 0.58 ( 0.58 ) | 0.6 ( 0.18 ) | -0.73 ( -2.46 ) |
| Sensory disturbance | 2 (0.4%) | 4.42 ( 0.86 - 22.86 ) | 4.41 ( 3.77 ) | 3.44 ( 0.87 ) | 1.78 ( -0.17 ) |
| Encephalitis | 2 (0.4%) | 11.07 ( 1.56 - 78.72 ) | 11.03 ( 9.13 ) | 6.02 ( 1.16 ) | 2.59 ( 0.48 ) |
| Delusion | 2 (0.4%) | 7.38 ( 1.23 - 44.24 ) | 7.35 ( 6.59 ) | 4.81 ( 1.07 ) | 2.27 ( 0.22 ) |
| Dysgraphia | 2 (0.4%) | 4.42 ( 0.86 - 22.86 ) | 4.41 ( 3.77 ) | 3.44 ( 0.87 ) | 1.78 ( -0.17 ) |
| Fine motor skill dysfunction | 2 (0.4%) | 7.38 ( 1.23 - 44.24 ) | 7.35 ( 6.59 ) | 4.81 ( 1.07 ) | 2.27 ( 0.22 ) |
| Atrial fibrillation | 1 (0.2%) | 0.37 ( 0.05 - 2.69 ) | 0.37 ( 1.06 ) | 0.39 ( 0.07 ) | -1.37 ( -3.47 ) |
| Electrocardiogram abnormal | 1 (0.2%) | 0.92 ( 0.12 - 7.08 ) | 0.92 ( 0.01 ) | 0.93 ( 0.17 ) | -0.11 ( -2.3 ) |
| Brain natriuretic peptide increased | 1 (0.2%) | 2.21 ( 0.26 - 18.94 ) | 2.21 ( 0.55 ) | 2.01 ( 0.33 ) | 1 ( -1.31 ) |
| Troponin normal | 1 (0.2%) | 5.52 ( 0.5 - 61.01 ) | 5.52 ( 2.47 ) | 4.01 ( 0.54 ) | 2 ( -0.5 ) |
| Ejection fraction normal | 1 (0.2%) | 3.68 ( 0.38 - 35.46 ) | 3.68 ( 1.46 ) | 3.01 ( 0.45 ) | 1.59 ( -0.83 ) |
| International normalised ratio increased | 1 (0.2%) | 11.05 ( 0.69 - 176.89 ) | 11.03 ( 4.56 ) | 6.02 ( 0.59 ) | 2.59 ( -0.05 ) |
| Allergy to vaccine | 1 (0.2%) | 5.52 ( 0.5 - 61.01 ) | 5.52 ( 2.47 ) | 4.01 ( 0.54 ) | 2 ( -0.5 ) |
| Dyspnoea exertional | 1 (0.2%) | 0.85 ( 0.11 - 6.5 ) | 0.85 ( 0.03 ) | 0.86 ( 0.16 ) | -0.22 ( -2.39 ) |
| Vertigo | 1 (0.2%) | 1.84 ( 0.22 - 15.31 ) | 1.84 ( 0.33 ) | 1.72 ( 0.29 ) | 0.78 ( -1.5 ) |
| Cough | 1 (0.2%) | 0.21 ( 0.03 - 1.56 ) | 0.22 ( 2.81 ) | 0.23 ( 0.04 ) | -2.11 ( -4.19 ) |
| Balance disorder | 1 (0.2%) | 0.79 ( 0.1 - 6 ) | 0.79 ( 0.05 ) | 0.8 ( 0.15 ) | -0.32 ( -2.48 ) |
| C-reactive protein increased | 1 (0.2%) | 1.58 ( 0.19 - 12.84 ) | 1.58 ( 0.18 ) | 1.5 ( 0.26 ) | 0.59 ( -1.67 ) |
| Pain in jaw | 1 (0.2%) | 11.05 ( 0.69 - 176.89 ) | 11.03 ( 4.56 ) | 6.02 ( 0.59 ) | 2.59 ( -0.05 ) |
| Catheterisation cardiac abnormal | 1 (0.2%) | 5.52 ( 0.5 - 61.01 ) | 5.52 ( 2.47 ) | 4.01 ( 0.54 ) | 2 ( -0.5 ) |
| Pericarditis | 1 (0.2%) | 1.23 ( 0.16 - 9.7 ) | 1.23 ( 0.04 ) | 1.2 ( 0.21 ) | 0.27 ( -1.95 ) |
| Electrocardiogram change | 1 (0.2%) | 5.52 ( 0.5 - 61.01 ) | 5.52 ( 2.47 ) | 4.01 ( 0.54 ) | 2 ( -0.5 ) |
| Cardio-respiratory arrest | 1 (0.2%) | 3.68 ( 0.38 - 35.46 ) | 3.68 ( 1.46 ) | 3.01 ( 0.45 ) | 1.59 ( -0.83 ) |
| Urinary tract infection | 1 (0.2%) | 0.61 ( 0.08 - 4.59 ) | 0.61 ( 0.23 ) | 0.63 ( 0.12 ) | -0.66 ( -2.8 ) |
| Respiratory arrest | 1 (0.2%) | 5.52 ( 0.5 - 61.01 ) | 5.52 ( 2.47 ) | 4.01 ( 0.54 ) | 2 ( -0.5 ) |
| Computerised tomogram spine normal | 1 (0.2%) | 5.52 ( 0.5 - 61.01 ) | 5.52 ( 2.47 ) | 4.01 ( 0.54 ) | 2 ( -0.5 ) |
| Platelet transfusion | 1 (0.2%) | 5.52 ( 0.5 - 61.01 ) | 5.52 ( 2.47 ) | 4.01 ( 0.54 ) | 2 ( -0.5 ) |
| Immune thrombocytopenia | 1 (0.2%) | 5.52 ( 0.5 - 61.01 ) | 5.52 ( 2.47 ) | 4.01 ( 0.54 ) | 2 ( -0.5 ) |
| Thrombocytopenia | 1 (0.2%) | 2.21 ( 0.26 - 18.94 ) | 2.21 ( 0.55 ) | 2.01 ( 0.33 ) | 1 ( -1.31 ) |
| Platelet count decreased | 1 (0.2%) | 1.58 ( 0.19 - 12.84 ) | 1.58 ( 0.18 ) | 1.5 ( 0.26 ) | 0.59 ( -1.67 ) |
| Csf glucose normal | 1 (0.2%) | 11.05 ( 0.69 - 176.89 ) | 11.03 ( 4.56 ) | 6.02 ( 0.59 ) | 2.59 ( -0.05 ) |
| Csf protein normal | 1 (0.2%) | 3.68 ( 0.38 - 35.46 ) | 3.68 ( 1.46 ) | 3.01 ( 0.45 ) | 1.59 ( -0.83 ) |
| Magnetic resonance imaging spinal abnormal | 1 (0.2%) | 2.21 ( 0.26 - 18.94 ) | 2.21 ( 0.55 ) | 2.01 ( 0.33 ) | 1 ( -1.31 ) |
| Csf red blood cell count positive | 1 (0.2%) | 2.76 ( 0.31 - 24.74 ) | 2.76 ( 0.9 ) | 2.41 ( 0.38 ) | 1.27 ( -1.09 ) |
| Vertebral foraminal stenosis | 1 (0.2%) | 11.05 ( 0.69 - 176.89 ) | 11.03 ( 4.56 ) | 6.02 ( 0.59 ) | 2.59 ( -0.05 ) |
| Deep vein thrombosis | 1 (0.2%) | 1.38 ( 0.17 - 11.05 ) | 1.38 ( 0.09 ) | 1.34 ( 0.23 ) | 0.42 ( -1.82 ) |
| Ultrasound scan | 1 (0.2%) | 2.21 ( 0.26 - 18.94 ) | 2.21 ( 0.55 ) | 2.01 ( 0.33 ) | 1 ( -1.31 ) |
| Cerebral infarction | 1 (0.2%) | 5.52 ( 0.5 - 61.01 ) | 5.52 ( 2.47 ) | 4.01 ( 0.54 ) | 2 ( -0.5 ) |
| Migraine | 1 (0.2%) | 2.21 ( 0.26 - 18.94 ) | 2.21 ( 0.55 ) | 2.01 ( 0.33 ) | 1 ( -1.31 ) |
| Chest discomfort | 1 (0.2%) | 0.85 ( 0.11 - 6.5 ) | 0.85 ( 0.03 ) | 0.86 ( 0.16 ) | -0.22 ( -2.39 ) |
| Immunisation reaction | 1 (0.2%) | 0.92 ( 0.12 - 7.08 ) | 0.92 ( 0.01 ) | 0.93 ( 0.17 ) | -0.11 ( -2.3 ) |
| Peripheral swelling | 1 (0.2%) | 0.73 ( 0.1 - 5.57 ) | 0.74 ( 0.09 ) | 0.75 ( 0.14 ) | -0.41 ( -2.57 ) |
| Hepatitis acute | 1 (0.2%) | 3.68 ( 0.38 - 35.46 ) | 3.68 ( 1.46 ) | 3.01 ( 0.45 ) | 1.59 ( -0.83 ) |
| Amnesia | 1 (0.2%) | 1.58 ( 0.19 - 12.84 ) | 1.58 ( 0.18 ) | 1.5 ( 0.26 ) | 0.59 ( -1.67 ) |
| Cognitive disorder | 1 (0.2%) | 1.58 ( 0.19 - 12.84 ) | 1.58 ( 0.18 ) | 1.5 ( 0.26 ) | 0.59 ( -1.67 ) |
| Speech disorder | 1 (0.2%) | 1.23 ( 0.16 - 9.7 ) | 1.23 ( 0.04 ) | 1.2 ( 0.21 ) | 0.27 ( -1.95 ) |
| Disorientation | 1 (0.2%) | 1.84 ( 0.22 - 15.31 ) | 1.84 ( 0.33 ) | 1.72 ( 0.29 ) | 0.78 ( -1.5 ) |
| Nervous system disorder | 1 (0.2%) | 5.52 ( 0.5 - 61.01 ) | 5.52 ( 2.47 ) | 4.01 ( 0.54 ) | 2 ( -0.5 ) |
| Atrial flutter | 1 (0.2%) | 3.68 ( 0.38 - 35.46 ) | 3.68 ( 1.46 ) | 3.01 ( 0.45 ) | 1.59 ( -0.83 ) |
| Cardiac failure congestive | 1 (0.2%) | 1.38 ( 0.17 - 11.05 ) | 1.38 ( 0.09 ) | 1.34 ( 0.23 ) | 0.42 ( -1.82 ) |
| Chronic obstructive pulmonary disease | 1 (0.2%) | 1.1 ( 0.14 - 8.63 ) | 1.1 ( 0.01 ) | 1.09 ( 0.2 ) | 0.13 ( -2.08 ) |
| Condition aggravated | 1 (0.2%) | 0.27 ( 0.04 - 1.95 ) | 0.27 ( 1.95 ) | 0.29 ( 0.05 ) | -1.8 ( -3.89 ) |
| Pneumonia | 1 (0.2%) | 0.35 ( 0.05 - 2.6 ) | 0.36 ( 1.14 ) | 0.38 ( 0.07 ) | -1.41 ( -3.52 ) |
| Hypophagia | 1 (0.2%) | 1.84 ( 0.22 - 15.31 ) | 1.84 ( 0.33 ) | 1.72 ( 0.29 ) | 0.78 ( -1.5 ) |
| Lethargy | 1 (0.2%) | 1 ( 0.13 - 7.78 ) | 1 ( 0 ) | 1 ( 0.18 ) | 0 ( -2.19 ) |
| Body temperature increased | 1 (0.2%) | 2.76 ( 0.31 - 24.74 ) | 2.76 ( 0.9 ) | 2.41 ( 0.38 ) | 1.27 ( -1.09 ) |
| Staphylococcal infection | 1 (0.2%) | 5.52 ( 0.5 - 61.01 ) | 5.52 ( 2.47 ) | 4.01 ( 0.54 ) | 2 ( -0.5 ) |
| Renal failure | 1 (0.2%) | 2.21 ( 0.26 - 18.94 ) | 2.21 ( 0.55 ) | 2.01 ( 0.33 ) | 1 ( -1.31 ) |
| Gastrointestinal wall thickening | 1 (0.2%) | 5.52 ( 0.5 - 61.01 ) | 5.52 ( 2.47 ) | 4.01 ( 0.54 ) | 2 ( -0.5 ) |
| Encephalopathy | 1 (0.2%) | 1.58 ( 0.19 - 12.84 ) | 1.58 ( 0.18 ) | 1.5 ( 0.26 ) | 0.59 ( -1.67 ) |
| Meningitis aseptic | 1 (0.2%) | 11.05 ( 0.69 - 176.89 ) | 11.03 ( 4.56 ) | 6.02 ( 0.59 ) | 2.59 ( -0.05 ) |
| Pleocytosis | 1 (0.2%) | 3.68 ( 0.38 - 35.46 ) | 3.68 ( 1.46 ) | 3.01 ( 0.45 ) | 1.59 ( -0.83 ) |
| Renal cyst | 1 (0.2%) | 2.76 ( 0.31 - 24.74 ) | 2.76 ( 0.9 ) | 2.41 ( 0.38 ) | 1.27 ( -1.09 ) |
| Acute respiratory failure | 1 (0.2%) | 0.85 ( 0.11 - 6.5 ) | 0.85 ( 0.03 ) | 0.86 ( 0.16 ) | -0.22 ( -2.39 ) |
| Hepatic steatosis | 1 (0.2%) | 3.68 ( 0.38 - 35.46 ) | 3.68 ( 1.46 ) | 3.01 ( 0.45 ) | 1.59 ( -0.83 ) |
| Pleural effusion | 1 (0.2%) | 1 ( 0.13 - 7.78 ) | 1 ( 0 ) | 1 ( 0.18 ) | 0 ( -2.19 ) |
| Osteoarthritis | 1 (0.2%) | 11.05 ( 0.69 - 176.89 ) | 11.03 ( 4.56 ) | 6.02 ( 0.59 ) | 2.59 ( -0.05 ) |
| Seizure | 1 (0.2%) | 1.23 ( 0.16 - 9.7 ) | 1.23 ( 0.04 ) | 1.2 ( 0.21 ) | 0.27 ( -1.95 ) |
| Agitation | 1 (0.2%) | 2.76 ( 0.31 - 24.74 ) | 2.76 ( 0.9 ) | 2.41 ( 0.38 ) | 1.27 ( -1.09 ) |
| Atelectasis | 1 (0.2%) | 2.21 ( 0.26 - 18.94 ) | 2.21 ( 0.55 ) | 2.01 ( 0.33 ) | 1 ( -1.31 ) |
| Computerised tomogram abdomen abnormal | 1 (0.2%) | 1.23 ( 0.16 - 9.7 ) | 1.23 ( 0.04 ) | 1.2 ( 0.21 ) | 0.27 ( -1.95 ) |
| Dysarthria | 1 (0.2%) | 1 ( 0.13 - 7.78 ) | 1 ( 0 ) | 1 ( 0.18 ) | 0 ( -2.19 ) |
| Illness | 1 (0.2%) | 1.23 ( 0.16 - 9.7 ) | 1.23 ( 0.04 ) | 1.2 ( 0.21 ) | 0.27 ( -1.95 ) |
| Sinus disorder | 1 (0.2%) | 11.05 ( 0.69 - 176.89 ) | 11.03 ( 4.56 ) | 6.02 ( 0.59 ) | 2.59 ( -0.05 ) |
| Blindness | 1 (0.2%) | 5.52 ( 0.5 - 61.01 ) | 5.52 ( 2.47 ) | 4.01 ( 0.54 ) | 2 ( -0.5 ) |
| Retinal artery occlusion | 1 (0.2%) | 11.05 ( 0.69 - 176.89 ) | 11.03 ( 4.56 ) | 6.02 ( 0.59 ) | 2.59 ( -0.05 ) |
| Retinal vein occlusion | 1 (0.2%) | 11.05 ( 0.69 - 176.89 ) | 11.03 ( 4.56 ) | 6.02 ( 0.59 ) | 2.59 ( -0.05 ) |
| Cerebral haemorrhage | 1 (0.2%) | 1.38 ( 0.17 - 11.05 ) | 1.38 ( 0.09 ) | 1.34 ( 0.23 ) | 0.42 ( -1.82 ) |
| Blood glucose decreased | 1 (0.2%) | 3.68 ( 0.38 - 35.46 ) | 3.68 ( 1.46 ) | 3.01 ( 0.45 ) | 1.59 ( -0.83 ) |
| Thinking abnormal | 1 (0.2%) | 11.05 ( 0.69 - 176.89 ) | 11.03 ( 4.56 ) | 6.02 ( 0.59 ) | 2.59 ( -0.05 ) |
| Cardioversion | 1 (0.2%) | 2.21 ( 0.26 - 18.94 ) | 2.21 ( 0.55 ) | 2.01 ( 0.33 ) | 1 ( -1.31 ) |
| Cardiac monitoring | 1 (0.2%) | 5.52 ( 0.5 - 61.01 ) | 5.52 ( 2.47 ) | 4.01 ( 0.54 ) | 2 ( -0.5 ) |
| Presyncope | 1 (0.2%) | 1.84 ( 0.22 - 15.31 ) | 1.84 ( 0.33 ) | 1.72 ( 0.29 ) | 0.78 ( -1.5 ) |
| Upper respiratory tract infection | 1 (0.2%) | 3.68 ( 0.38 - 35.46 ) | 3.68 ( 1.46 ) | 3.01 ( 0.45 ) | 1.59 ( -0.83 ) |
| X-ray limb abnormal | 1 (0.2%) | 5.52 ( 0.5 - 61.01 ) | 5.52 ( 2.47 ) | 4.01 ( 0.54 ) | 2 ( -0.5 ) |
| Hypotonia | 1 (0.2%) | 5.52 ( 0.5 - 61.01 ) | 5.52 ( 2.47 ) | 4.01 ( 0.54 ) | 2 ( -0.5 ) |
| Covid-19 | 1 (0.2%) | 0.08 ( 0.01 - 0.61 ) | 0.09 ( 9.8 ) | 0.09 ( 0.02 ) | -3.42 ( -5.48 ) |
| Sars-cov-2 test positive | 1 (0.2%) | 0.12 ( 0.02 - 0.86 ) | 0.12 ( 6.39 ) | 0.13 ( 0.03 ) | -2.93 ( -5 ) |
| Acoustic stimulation tests | 1 (0.2%) | 11.05 ( 0.69 - 176.89 ) | 11.03 ( 4.56 ) | 6.02 ( 0.59 ) | 2.59 ( -0.05 ) |
| Deafness | 1 (0.2%) | 11.05 ( 0.69 - 176.89 ) | 11.03 ( 4.56 ) | 6.02 ( 0.59 ) | 2.59 ( -0.05 ) |
| Hemiparesis | 1 (0.2%) | 1.38 ( 0.17 - 11.05 ) | 1.38 ( 0.09 ) | 1.34 ( 0.23 ) | 0.42 ( -1.82 ) |
| Pruritus | 1 (0.2%) | 2.21 ( 0.26 - 18.94 ) | 2.21 ( 0.55 ) | 2.01 ( 0.33 ) | 1 ( -1.31 ) |
| Rash erythematous | 1 (0.2%) | 11.05 ( 0.69 - 176.89 ) | 11.03 ( 4.56 ) | 6.02 ( 0.59 ) | 2.59 ( -0.05 ) |
| Feeding disorder | 1 (0.2%) | 2.76 ( 0.31 - 24.74 ) | 2.76 ( 0.9 ) | 2.41 ( 0.38 ) | 1.27 ( -1.09 ) |
| Hypotension | 1 (0.2%) | 0.61 ( 0.08 - 4.59 ) | 0.61 ( 0.23 ) | 0.63 ( 0.12 ) | -0.66 ( -2.8 ) |
| Cystitis | 1 (0.2%) | 5.52 ( 0.5 - 61.01 ) | 5.52 ( 2.47 ) | 4.01 ( 0.54 ) | 2 ( -0.5 ) |
| Hypothyroidism | 1 (0.2%) | 3.68 ( 0.38 - 35.46 ) | 3.68 ( 1.46 ) | 3.01 ( 0.45 ) | 1.59 ( -0.83 ) |
| Photopsia | 1 (0.2%) | 11.05 ( 0.69 - 176.89 ) | 11.03 ( 4.56 ) | 6.02 ( 0.59 ) | 2.59 ( -0.05 ) |
| Swollen tongue | 1 (0.2%) | 5.52 ( 0.5 - 61.01 ) | 5.52 ( 2.47 ) | 4.01 ( 0.54 ) | 2 ( -0.5 ) |
| Polymyalgia rheumatica | 1 (0.2%) | 11.05 ( 0.69 - 176.89 ) | 11.03 ( 4.56 ) | 6.02 ( 0.59 ) | 2.59 ( -0.05 ) |
| Herpes zoster | 1 (0.2%) | 2.21 ( 0.26 - 18.94 ) | 2.21 ( 0.55 ) | 2.01 ( 0.33 ) | 1 ( -1.31 ) |
| Meningitis | 1 (0.2%) | 3.68 ( 0.38 - 35.46 ) | 3.68 ( 1.46 ) | 3.01 ( 0.45 ) | 1.59 ( -0.83 ) |
| Scab | 1 (0.2%) | 11.05 ( 0.69 - 176.89 ) | 11.03 ( 4.56 ) | 6.02 ( 0.59 ) | 2.59 ( -0.05 ) |
| Wound | 1 (0.2%) | 11.05 ( 0.69 - 176.89 ) | 11.03 ( 4.56 ) | 6.02 ( 0.59 ) | 2.59 ( -0.05 ) |
| Burning sensation | 1 (0.2%) | 5.52 ( 0.5 - 61.01 ) | 5.52 ( 2.47 ) | 4.01 ( 0.54 ) | 2 ( -0.5 ) |
| Scar | 1 (0.2%) | 11.05 ( 0.69 - 176.89 ) | 11.03 ( 4.56 ) | 6.02 ( 0.59 ) | 2.59 ( -0.05 ) |
| Contusion | 1 (0.2%) | 1.38 ( 0.17 - 11.05 ) | 1.38 ( 0.09 ) | 1.34 ( 0.23 ) | 0.42 ( -1.82 ) |
| Nerve injury | 1 (0.2%) | 3.68 ( 0.38 - 35.46 ) | 3.68 ( 1.46 ) | 3.01 ( 0.45 ) | 1.59 ( -0.83 ) |
| Sensory loss | 1 (0.2%) | 11.05 ( 0.69 - 176.89 ) | 11.03 ( 4.56 ) | 6.02 ( 0.59 ) | 2.59 ( -0.05 ) |
| Urinary retention | 1 (0.2%) | 11.05 ( 0.69 - 176.89 ) | 11.03 ( 4.56 ) | 6.02 ( 0.59 ) | 2.59 ( -0.05 ) |
| Syncope | 1 (0.2%) | 0.52 ( 0.07 - 3.91 ) | 0.53 ( 0.41 ) | 0.55 ( 0.1 ) | -0.87 ( -3 ) |
| White blood cell count increased | 1 (0.2%) | 0.85 ( 0.11 - 6.5 ) | 0.85 ( 0.03 ) | 0.86 ( 0.16 ) | -0.22 ( -2.39 ) |
| Diabetes mellitus | 1 (0.2%) | 5.52 ( 0.5 - 61.01 ) | 5.52 ( 2.47 ) | 4.01 ( 0.54 ) | 2 ( -0.5 ) |
| Lymphadenopathy | 1 (0.2%) | 2.21 ( 0.26 - 18.94 ) | 2.21 ( 0.55 ) | 2.01 ( 0.33 ) | 1 ( -1.31 ) |
| Pulmonary embolism | 1 (0.2%) | 0.58 ( 0.08 - 4.34 ) | 0.58 ( 0.29 ) | 0.6 ( 0.11 ) | -0.73 ( -2.87 ) |
| Inflammation | 1 (0.2%) | 3.68 ( 0.38 - 35.46 ) | 3.68 ( 1.46 ) | 3.01 ( 0.45 ) | 1.59 ( -0.83 ) |
| Muscle strain | 1 (0.2%) | 11.05 ( 0.69 - 176.89 ) | 11.03 ( 4.56 ) | 6.02 ( 0.59 ) | 2.59 ( -0.05 ) |
| Rotator cuff syndrome | 1 (0.2%) | 11.05 ( 0.69 - 176.89 ) | 11.03 ( 4.56 ) | 6.02 ( 0.59 ) | 2.59 ( -0.05 ) |
| Injury | 1 (0.2%) | 2.76 ( 0.31 - 24.74 ) | 2.76 ( 0.9 ) | 2.41 ( 0.38 ) | 1.27 ( -1.09 ) |
| Joint range of motion decreased | 1 (0.2%) | 3.68 ( 0.38 - 35.46 ) | 3.68 ( 1.46 ) | 3.01 ( 0.45 ) | 1.59 ( -0.83 ) |
| Vaccination site pain | 1 (0.2%) | 1.84 ( 0.22 - 15.31 ) | 1.84 ( 0.33 ) | 1.72 ( 0.29 ) | 0.78 ( -1.5 ) |
| Dyskinesia | 1 (0.2%) | 5.52 ( 0.5 - 61.01 ) | 5.52 ( 2.47 ) | 4.01 ( 0.54 ) | 2 ( -0.5 ) |
| Hemiplegia | 1 (0.2%) | 5.52 ( 0.5 - 61.01 ) | 5.52 ( 2.47 ) | 4.01 ( 0.54 ) | 2 ( -0.5 ) |
| Periarthritis | 1 (0.2%) | 11.05 ( 0.69 - 176.89 ) | 11.03 ( 4.56 ) | 6.02 ( 0.59 ) | 2.59 ( -0.05 ) |
| Tremor | 1 (0.2%) | 1.38 ( 0.17 - 11.05 ) | 1.38 ( 0.09 ) | 1.34 ( 0.23 ) | 0.42 ( -1.82 ) |
| Laboratory test abnormal | 1 (0.2%) | 1.38 ( 0.17 - 11.05 ) | 1.38 ( 0.09 ) | 1.34 ( 0.23 ) | 0.42 ( -1.82 ) |
| Muscle spasms | 1 (0.2%) | 5.52 ( 0.5 - 61.01 ) | 5.52 ( 2.47 ) | 4.01 ( 0.54 ) | 2 ( -0.5 ) |
| Head injury | 1 (0.2%) | 1.58 ( 0.19 - 12.84 ) | 1.58 ( 0.18 ) | 1.5 ( 0.26 ) | 0.59 ( -1.67 ) |
| Ascending flaccid paralysis | 1 (0.2%) | 3.68 ( 0.38 - 35.46 ) | 3.68 ( 1.46 ) | 3.01 ( 0.45 ) | 1.59 ( -0.83 ) |
| Personality change | 1 (0.2%) | 11.05 ( 0.69 - 176.89 ) | 11.03 ( 4.56 ) | 6.02 ( 0.59 ) | 2.59 ( -0.05 ) |
| Positive airway pressure therapy | 1 (0.2%) | 1.84 ( 0.22 - 15.31 ) | 1.84 ( 0.33 ) | 1.72 ( 0.29 ) | 0.78 ( -1.5 ) |
| **RSV + COVID-19 + Influenza Vaccines** | | | | | |
| **PT** | **N(%)** | **ROR(95%Cl)** | **PRR(卡方值)** | **EBGM(EBGM05)** | **IC(IC025)** |
| Mobility decreased | 5 (3.1%) | 7.99 ( 3.03 - 21.07 ) | 7.77 ( 24.85 ) | 6.68 ( 2.96 ) | 2.74 ( 1.44 ) |
| Guillain-barre syndrome | 4 (2.5%) | 5.69 ( 1.98 - 16.38 ) | 5.57 ( 13.25 ) | 5.02 ( 2.07 ) | 2.33 ( 0.93 ) |
| Muscular weakness | 4 (2.5%) | 4.02 ( 1.42 - 11.35 ) | 3.94 ( 8.05 ) | 3.68 ( 1.54 ) | 1.88 ( 0.5 ) |
| Gait disturbance | 3 (1.9%) | 3.72 ( 1.13 - 12.27 ) | 3.67 ( 5.38 ) | 3.45 ( 1.27 ) | 1.79 ( 0.25 ) |
| Feeling abnormal | 3 (1.9%) | 10.27 ( 2.87 - 36.76 ) | 10.1 ( 19.72 ) | 8.28 ( 2.85 ) | 3.05 ( 1.42 ) |
| Cerebrovascular accident | 3 (1.9%) | 4.1 ( 1.24 - 13.57 ) | 4.04 ( 6.27 ) | 3.76 ( 1.38 ) | 1.91 ( 0.37 ) |
| Mental status changes | 3 (1.9%) | 10.27 ( 2.87 - 36.76 ) | 10.1 ( 19.72 ) | 8.28 ( 2.85 ) | 3.05 ( 1.42 ) |
| Aphasia | 2 (1.2%) | 6.81 ( 1.51 - 30.66 ) | 6.73 ( 8.39 ) | 5.91 ( 1.68 ) | 2.56 ( 0.73 ) |
| Neck pain | 2 (1.2%) | 10.21 ( 2.15 - 48.48 ) | 10.1 ( 13.14 ) | 8.28 ( 2.25 ) | 3.05 ( 1.16 ) |
| Delusion | 2 (1.2%) | 27.26 ( 4.52 - 164.26 ) | 26.93 ( 29.99 ) | 16.56 ( 3.68 ) | 4.05 ( 2 ) |
| Sepsis | 2 (1.2%) | 3.26 ( 0.77 - 13.88 ) | 3.23 ( 2.87 ) | 3.07 ( 0.91 ) | 1.62 ( -0.15 ) |
| Dysgraphia | 2 (1.2%) | 16.35 ( 3.15 - 84.91 ) | 16.16 ( 20.34 ) | 11.83 ( 2.98 ) | 3.56 ( 1.6 ) |
| Fine motor skill dysfunction | 2 (1.2%) | 27.26 ( 4.52 - 164.26 ) | 26.93 ( 29.99 ) | 16.56 ( 3.68 ) | 4.05 ( 2 ) |
| Deep vein thrombosis | 1 (0.6%) | 5.08 ( 0.63 - 40.82 ) | 5.05 ( 2.89 ) | 4.6 ( 0.8 ) | 2.2 ( -0.04 ) |
| Chest discomfort | 1 (0.6%) | 3.12 ( 0.41 - 24 ) | 3.11 ( 1.33 ) | 2.96 ( 0.54 ) | 1.56 ( -0.62 ) |
| Hepatitis acute | 1 (0.6%) | 13.54 ( 1.4 - 130.92 ) | 13.47 ( 8.66 ) | 10.35 ( 1.55 ) | 3.37 ( 0.95 ) |
| Chest pain | 1 (0.6%) | 1.69 ( 0.23 - 12.55 ) | 1.68 ( 0.27 ) | 1.66 ( 0.31 ) | 0.73 ( -1.4 ) |
| Hypertension | 1 (0.6%) | 2.13 ( 0.28 - 16.03 ) | 2.13 ( 0.57 ) | 2.07 ( 0.38 ) | 1.05 ( -1.1 ) |
| Amnesia | 1 (0.6%) | 5.8 ( 0.71 - 47.43 ) | 5.77 ( 3.46 ) | 5.17 ( 0.89 ) | 2.37 ( 0.11 ) |
| Cognitive disorder | 1 (0.6%) | 5.8 ( 0.71 - 47.43 ) | 5.77 ( 3.46 ) | 5.17 ( 0.89 ) | 2.37 ( 0.11 ) |
| Incoherent | 1 (0.6%) | 5.08 ( 0.63 - 40.82 ) | 5.05 ( 2.89 ) | 4.6 ( 0.8 ) | 2.2 ( -0.04 ) |
| Computerised tomogram abnormal | 1 (0.6%) | 13.54 ( 1.4 - 130.92 ) | 13.47 ( 8.66 ) | 10.35 ( 1.55 ) | 3.37 ( 0.95 ) |
| Dysstasia | 1 (0.6%) | 2.13 ( 0.28 - 16.03 ) | 2.13 ( 0.57 ) | 2.07 ( 0.38 ) | 1.05 ( -1.1 ) |
| Magnetic resonance imaging abnormal | 1 (0.6%) | 13.54 ( 1.4 - 130.92 ) | 13.47 ( 8.66 ) | 10.35 ( 1.55 ) | 3.37 ( 0.95 ) |
| Speech disorder | 1 (0.6%) | 4.51 ( 0.57 - 35.81 ) | 4.49 ( 2.44 ) | 4.14 ( 0.73 ) | 2.05 ( -0.18 ) |
| Confusional state | 1 (0.6%) | 1.45 ( 0.2 - 10.69 ) | 1.44 ( 0.13 ) | 1.43 ( 0.27 ) | 0.51 ( -1.6 ) |
| Hypophagia | 1 (0.6%) | 6.77 ( 0.81 - 56.55 ) | 6.73 ( 4.19 ) | 5.91 ( 1 ) | 2.56 ( 0.27 ) |
| Lethargy | 1 (0.6%) | 3.69 ( 0.47 - 28.75 ) | 3.67 ( 1.79 ) | 3.45 ( 0.62 ) | 1.79 ( -0.42 ) |
| Blindness | 1 (0.6%) | 20.32 ( 1.83 - 225.24 ) | 20.2 ( 12.17 ) | 13.8 ( 1.84 ) | 3.79 ( 1.28 ) |
| Retinal artery occlusion | 1 (0.6%) | 40.64 ( 2.53 - 652.71 ) | 40.4 ( 19.22 ) | 20.7 ( 2.03 ) | 4.37 ( 1.73 ) |
| Retinal vein occlusion | 1 (0.6%) | 40.64 ( 2.53 - 652.71 ) | 40.4 ( 19.22 ) | 20.7 ( 2.03 ) | 4.37 ( 1.73 ) |
| Cerebral haemorrhage | 1 (0.6%) | 5.08 ( 0.63 - 40.82 ) | 5.05 ( 2.89 ) | 4.6 ( 0.8 ) | 2.2 ( -0.04 ) |
| Upper respiratory tract infection | 1 (0.6%) | 13.54 ( 1.4 - 130.92 ) | 13.47 ( 8.66 ) | 10.35 ( 1.55 ) | 3.37 ( 0.95 ) |
| Musculoskeletal disorder | 1 (0.6%) | 4.51 ( 0.57 - 35.81 ) | 4.49 ( 2.44 ) | 4.14 ( 0.73 ) | 2.05 ( -0.18 ) |
| Red blood cell sedimentation rate increased | 1 (0.6%) | 20.32 ( 1.83 - 225.24 ) | 20.2 ( 12.17 ) | 13.8 ( 1.84 ) | 3.79 ( 1.28 ) |
| Hypotonia | 1 (0.6%) | 20.32 ( 1.83 - 225.24 ) | 20.2 ( 12.17 ) | 13.8 ( 1.84 ) | 3.79 ( 1.28 ) |
| Unresponsive to stimuli | 1 (0.6%) | 2.9 ( 0.38 - 22.17 ) | 2.89 ( 1.15 ) | 2.76 ( 0.5 ) | 1.46 ( -0.71 ) |
| Magnetic resonance imaging head abnormal | 1 (0.6%) | 3.38 ( 0.44 - 26.16 ) | 3.37 ( 1.54 ) | 3.18 ( 0.57 ) | 1.67 ( -0.52 ) |
| Hemiparesis | 1 (0.6%) | 5.08 ( 0.63 - 40.82 ) | 5.05 ( 2.89 ) | 4.6 ( 0.8 ) | 2.2 ( -0.04 ) |
| Resuscitation | 1 (0.6%) | 3.69 ( 0.47 - 28.75 ) | 3.67 ( 1.79 ) | 3.45 ( 0.62 ) | 1.79 ( -0.42 ) |
| Pruritus | 1 (0.6%) | 8.12 ( 0.94 - 69.94 ) | 8.08 ( 5.17 ) | 6.9 ( 1.14 ) | 2.79 ( 0.46 ) |
| Rash erythematous | 1 (0.6%) | 40.64 ( 2.53 - 652.71 ) | 40.4 ( 19.22 ) | 20.7 ( 2.03 ) | 4.37 ( 1.73 ) |
| Sleep disorder | 1 (0.6%) | 4.06 ( 0.52 - 31.9 ) | 4.04 ( 2.08 ) | 3.76 ( 0.67 ) | 1.91 ( -0.3 ) |
| Csf white blood cell count increased | 1 (0.6%) | 8.12 ( 0.94 - 69.94 ) | 8.08 ( 5.17 ) | 6.9 ( 1.14 ) | 2.79 ( 0.46 ) |
| Encephalitis | 1 (0.6%) | 13.54 ( 1.4 - 130.92 ) | 13.47 ( 8.66 ) | 10.35 ( 1.55 ) | 3.37 ( 0.95 ) |
| Gait inability | 1 (0.6%) | 2.13 ( 0.28 - 16.03 ) | 2.13 ( 0.57 ) | 2.07 ( 0.38 ) | 1.05 ( -1.1 ) |
| Hypoaesthesia | 1 (0.6%) | 1.62 ( 0.22 - 12.03 ) | 1.62 ( 0.23 ) | 1.59 ( 0.3 ) | 0.67 ( -1.45 ) |
| Syncope | 1 (0.6%) | 1.93 ( 0.26 - 14.43 ) | 1.92 ( 0.42 ) | 1.88 ( 0.35 ) | 0.91 ( -1.23 ) |
| White blood cell count increased | 1 (0.6%) | 3.12 ( 0.41 - 24 ) | 3.11 ( 1.33 ) | 2.96 ( 0.54 ) | 1.56 ( -0.62 ) |
| Diabetes mellitus | 1 (0.6%) | 20.32 ( 1.83 - 225.24 ) | 20.2 ( 12.17 ) | 13.8 ( 1.84 ) | 3.79 ( 1.28 ) |
| Lymphadenopathy | 1 (0.6%) | 8.12 ( 0.94 - 69.94 ) | 8.08 ( 5.17 ) | 6.9 ( 1.14 ) | 2.79 ( 0.46 ) |
| Pulmonary embolism | 1 (0.6%) | 2.13 ( 0.28 - 16.03 ) | 2.13 ( 0.57 ) | 2.07 ( 0.38 ) | 1.05 ( -1.1 ) |
| Hemiplegia | 1 (0.6%) | 20.32 ( 1.83 - 225.24 ) | 20.2 ( 12.17 ) | 13.8 ( 1.84 ) | 3.79 ( 1.28 ) |
| Arthralgia | 1 (0.6%) | 2.13 ( 0.28 - 16.03 ) | 2.13 ( 0.57 ) | 2.07 ( 0.38 ) | 1.05 ( -1.1 ) |
| Periarthritis | 1 (0.6%) | 40.64 ( 2.53 - 652.71 ) | 40.4 ( 19.22 ) | 20.7 ( 2.03 ) | 4.37 ( 1.73 ) |
| Pain in extremity | 1 (0.6%) | 1.09 ( 0.15 - 8.01 ) | 1.09 ( 0.01 ) | 1.09 ( 0.21 ) | 0.12 ( -1.98 ) |
| Personality change | 1 (0.6%) | 40.64 ( 2.53 - 652.71 ) | 40.4 ( 19.22 ) | 20.7 ( 2.03 ) | 4.37 ( 1.73 ) |
| Nausea | 1 (0.6%) | 0.99 ( 0.13 - 7.21 ) | 0.99 ( 0 ) | 0.99 ( 0.19 ) | -0.02 ( -2.12 ) |

**Table S5:** Distribution and signal strength of adverse events in non-serious reports across the three co-administration vaccination groups.

| **RSV + COVID-19 Vaccines** | | | | | | | | | | | |
| --- | --- | --- | --- | --- | --- | --- | --- | --- | --- | --- | --- |
| **PT** | **N(%)** | | **ROR(95%Cl)** | | | **PRR(X^2^)** | | | **EBGM(EBGM05)** | | **IC(IC025)** |
| Headache | 52 (3.5%) | | 1.61 ( 1.19 - 2.17 ) | | | 1.59 ( 9.87 ) | | | 1.5 ( 1.17 ) | | 0.59 ( 0.16 ) |
| Fatigue | 47 (3.1%) | | 1.43 ( 1.04 - 1.95 ) | | | 1.41 ( 5 ) | | | 1.36 ( 1.04 ) | | 0.44 ( -0.01 ) |
| Dizziness | 37 (2.5%) | | 1.43 ( 1.01 - 2.03 ) | | | 1.42 ( 4.03 ) | | | 1.36 ( 1.02 ) | | 0.45 ( -0.06 ) |
| Asthenia | 30 (2.0%) | | 2.05 ( 1.38 - 3.06 ) | | | 2.03 ( 12.99 ) | | | 1.84 ( 1.32 ) | | 0.88 ( 0.31 ) |
| Influenza like illness | 16 (1.1%) | | 3.03 ( 1.71 - 5.34 ) | | | 3 ( 16.11 ) | | | 2.5 ( 1.56 ) | | 1.32 ( 0.54 ) |
| Sleep disorder | 16 (1.1%) | | 2.55 ( 1.46 - 4.44 ) | | | 2.53 ( 11.61 ) | | | 2.19 ( 1.38 ) | | 1.13 ( 0.36 ) |
| Hyperhidrosis | 15 (1.0%) | | 2.34 ( 1.33 - 4.15 ) | | | 2.33 ( 9.1 ) | | | 2.06 ( 1.28 ) | | 1.04 ( 0.24 ) |
| Decreased appetite | 12 (0.8%) | | 1.94 ( 1.04 - 3.62 ) | | | 1.93 ( 4.46 ) | | | 1.77 ( 1.05 ) | | 0.82 ( -0.05 ) |
| Gait disturbance | 12 (0.8%) | | 1.9 ( 1.02 - 3.56 ) | | | 1.9 ( 4.23 ) | | | 1.74 ( 1.03 ) | | 0.8 ( -0.07 ) |
| Tenderness | 11 (0.7%) | | 2.93 ( 1.48 - 5.79 ) | | | 2.92 ( 10.49 ) | | | 2.45 ( 1.38 ) | | 1.29 ( 0.35 ) |
| Vertigo | 9 (0.6%) | | 2.63 ( 1.25 - 5.53 ) | | | 2.62 ( 6.98 ) | | | 2.25 ( 1.21 ) | | 1.17 ( 0.15 ) |
| Muscular weakness | 9 (0.6%) | | 1.27 ( 0.63 - 2.55 ) | | | 1.27 ( 0.45 ) | | | 1.23 ( 0.69 ) | | 0.3 ( -0.67 ) |
| Gait inability | 8 (0.5%) | | 4.83 ( 2.04 - 11.4 ) | | | 4.81 ( 15.75 ) | | | 3.48 ( 1.7 ) | | 1.8 ( 0.67 ) |
| Back pain | 8 (0.5%) | | 1.2 ( 0.57 - 2.52 ) | | | 1.2 ( 0.24 ) | | | 1.18 ( 0.63 ) | | 0.24 ( -0.79 ) |
| Condition aggravated | 8 (0.5%) | | 1.08 ( 0.52 - 2.25 ) | | | 1.08 ( 0.04 ) | | | 1.07 ( 0.58 ) | | 0.09 ( -0.92 ) |
| Rash erythematous | 8 (0.5%) | | 1.2 ( 0.57 - 2.52 ) | | | 1.2 ( 0.24 ) | | | 1.18 ( 0.63 ) | | 0.24 ( -0.79 ) |
| Skin warm | 8 (0.5%) | | 0.92 ( 0.45 - 1.92 ) | | | 0.92 ( 0.05 ) | | | 0.93 ( 0.51 ) | | -0.1 ( -1.11 ) |
| Syncope | 7 (0.5%) | | 1.05 ( 0.48 - 2.3 ) | | | 1.05 ( 0.02 ) | | | 1.05 ( 0.54 ) | | 0.07 ( -1.02 ) |
| Muscle spasms | 7 (0.5%) | | 2.43 ( 1.05 - 5.62 ) | | | 2.43 ( 4.64 ) | | | 2.12 ( 1.05 ) | | 1.09 ( -0.05 ) |
| Feeling abnormal | 7 (0.5%) | | 1.05 ( 0.48 - 2.3 ) | | | 1.05 ( 0.02 ) | | | 1.05 ( 0.54 ) | | 0.07 ( -1.02 ) |
| Oropharyngeal pain | 6 (0.4%) | | 0.89 ( 0.38 - 2.05 ) | | | 0.89 ( 0.08 ) | | | 0.9 ( 0.44 ) | | -0.16 ( -1.3 ) |
| Brain fog | 6 (0.4%) | | 2.36 ( 0.96 - 5.8 ) | | | 2.35 ( 3.7 ) | | | 2.07 ( 0.98 ) | | 1.05 ( -0.16 ) |
| Injected limb mobility decreased | 6 (0.4%) | | 0.87 ( 0.38 - 2.02 ) | | | 0.87 ( 0.1 ) | | | 0.88 ( 0.44 ) | | -0.18 ( -1.32 ) |
| Feeling cold | 6 (0.4%) | | 2.08 ( 0.86 - 5.07 ) | | | 2.08 ( 2.74 ) | | | 1.88 ( 0.89 ) | | 0.91 ( -0.29 ) |
| Musculoskeletal stiffness | 6 (0.4%) | | 1.32 ( 0.56 - 3.11 ) | | | 1.32 ( 0.4 ) | | | 1.28 ( 0.62 ) | | 0.35 ( -0.81 ) |
| Neck pain | 6 (0.4%) | | 1.39 ( 0.59 - 3.28 ) | | | 1.39 ( 0.56 ) | | | 1.34 ( 0.65 ) | | 0.42 ( -0.76 ) |
| Confusional state | 6 (0.4%) | | 1.87 ( 0.77 - 4.51 ) | | | 1.86 ( 2 ) | | | 1.72 ( 0.82 ) | | 0.78 ( -0.41 ) |
| Hypoaesthesia | 6 (0.4%) | | 1.26 ( 0.53 - 2.96 ) | | | 1.26 ( 0.28 ) | | | 1.23 ( 0.6 ) | | 0.29 ( -0.87 ) |
| Rhinorrhoea | 6 (0.4%) | | 1.59 ( 0.67 - 3.8 ) | | | 1.59 ( 1.12 ) | | | 1.5 ( 0.73 ) | | 0.59 ( -0.59 ) |
| Injection site mass | 5 (0.3%) | | 2.82 ( 1.03 - 7.72 ) | | | 2.82 ( 4.47 ) | | | 2.38 ( 1.03 ) | | 1.25 ( -0.08 ) |
| Chest pain | 5 (0.3%) | | 0.85 ( 0.34 - 2.13 ) | | | 0.85 ( 0.12 ) | | | 0.86 ( 0.4 ) | | -0.21 ( -1.45 ) |
| Induration | 4 (0.3%) | | 5.16 ( 1.51 - 17.65 ) | | | 5.15 ( 8.52 ) | | | 3.64 ( 1.3 ) | | 1.86 ( 0.33 ) |
| Injection site induration | 4 (0.3%) | | 2.12 ( 0.71 - 6.32 ) | | | 2.12 ( 1.92 ) | | | 1.91 ( 0.77 ) | | 0.93 ( -0.5 ) |
| Urinary incontinence | 4 (0.3%) | | 5.16 ( 1.51 - 17.65 ) | | | 5.15 ( 8.52 ) | | | 3.64 ( 1.3 ) | | 1.86 ( 0.33 ) |
| Palpitations | 4 (0.3%) | | 0.84 ( 0.3 - 2.34 ) | | | 0.84 ( 0.11 ) | | | 0.85 ( 0.36 ) | | -0.23 ( -1.59 ) |
| Discomfort | 4 (0.3%) | | 1.8 ( 0.62 - 5.29 ) | | | 1.8 ( 1.19 ) | | | 1.67 ( 0.68 ) | | 0.74 ( -0.68 ) |
| Cellulitis | 4 (0.3%) | | 1.72 ( 0.59 - 5.01 ) | | | 1.72 ( 1.01 ) | | | 1.6 ( 0.65 ) | | 0.68 ( -0.73 ) |
| Aphasia | 4 (0.3%) | | 9.03 ( 2.26 - 36.16 ) | | | 9.01 ( 14.25 ) | | | 5.01 ( 1.57 ) | | 2.32 ( 0.71 ) |
| Loss of consciousness | 4 (0.3%) | | 0.78 ( 0.28 - 2.18 ) | | | 0.78 ( 0.22 ) | | | 0.8 ( 0.34 ) | | -0.32 ( -1.67 ) |
| Hypersomnia | 4 (0.3%) | | 2.26 ( 0.75 - 6.76 ) | | | 2.25 ( 2.23 ) | | | 2 ( 0.8 ) | | 1 ( -0.44 ) |
| Paraesthesia | 4 (0.3%) | | 0.69 ( 0.25 - 1.92 ) | | | 0.69 ( 0.51 ) | | | 0.72 ( 0.31 ) | | -0.48 ( -1.83 ) |
| Tinnitus | 4 (0.3%) | | 1.5 ( 0.52 - 4.34 ) | | | 1.5 ( 0.58 ) | | | 1.43 ( 0.59 ) | | 0.52 ( -0.88 ) |
| Vision blurred | 3 (0.2%) | | 0.97 ( 0.29 - 3.18 ) | | | 0.97 ( 0 ) | | | 0.97 ( 0.36 ) | | -0.05 ( -1.58 ) |
| Somnolence | 3 (0.2%) | | 1.29 ( 0.38 - 4.32 ) | | | 1.29 ( 0.17 ) | | | 1.25 ( 0.45 ) | | 0.32 ( -1.23 ) |
| Heart rate increased | 3 (0.2%) | | 0.84 ( 0.26 - 2.76 ) | | | 0.84 ( 0.08 ) | | | 0.86 ( 0.32 ) | | -0.22 ( -1.74 ) |
| Blister | 3 (0.2%) | | 1.5 ( 0.44 - 5.11 ) | | | 1.5 ( 0.43 ) | | | 1.43 ( 0.51 ) | | 0.52 ( -1.05 ) |
| Swelling face | 3 (0.2%) | | 1.23 ( 0.37 - 4.11 ) | | | 1.23 ( 0.11 ) | | | 1.2 ( 0.44 ) | | 0.26 ( -1.29 ) |
| Dysphonia | 3 (0.2%) | | 2.46 ( 0.69 - 8.83 ) | | | 2.46 ( 2.04 ) | | | 2.15 ( 0.74 ) | | 1.1 ( -0.52 ) |
| Feeling hot | 3 (0.2%) | | 0.68 ( 0.21 - 2.19 ) | | | 0.68 ( 0.43 ) | | | 0.7 ( 0.26 ) | | -0.52 ( -2.03 ) |
| Respiratory tract congestion | 3 (0.2%) | | 0.84 ( 0.26 - 2.76 ) | | | 0.84 ( 0.08 ) | | | 0.86 ( 0.32 ) | | -0.22 ( -1.74 ) |
| Chest discomfort | 3 (0.2%) | | 0.66 ( 0.2 - 2.13 ) | | | 0.66 ( 0.49 ) | | | 0.68 ( 0.26 ) | | -0.55 ( -2.06 ) |
| Nasopharyngitis | 3 (0.2%) | | 0.93 ( 0.28 - 3.06 ) | | | 0.93 ( 0.01 ) | | | 0.94 ( 0.35 ) | | -0.09 ( -1.62 ) |
| Lymphadenopathy | 3 (0.2%) | | 1.5 ( 0.44 - 5.11 ) | | | 1.5 ( 0.43 ) | | | 1.43 ( 0.51 ) | | 0.52 ( -1.05 ) |
| Lethargy | 3 (0.2%) | | 0.69 ( 0.21 - 2.24 ) | | | 0.69 ( 0.38 ) | | | 0.72 ( 0.27 ) | | -0.48 ( -1.99 ) |
| Contusion | 3 (0.2%) | | 0.53 ( 0.16 - 1.7 ) | | | 0.53 ( 1.18 ) | | | 0.56 ( 0.21 ) | | -0.85 ( -2.34 ) |
| Productive cough | 3 (0.2%) | | 2.08 ( 0.59 - 7.31 ) | | | 2.08 ( 1.37 ) | | | 1.88 ( 0.66 ) | | 0.91 ( -0.7 ) |
| Blood glucose increased | 3 (0.2%) | | 4.51 ( 1.13 - 18.07 ) | | | 4.51 ( 5.46 ) | | | 3.34 ( 1.05 ) | | 1.74 ( 0.03 ) |
| Rash pruritic | 3 (0.2%) | | 0.52 ( 0.16 - 1.66 ) | | | 0.52 ( 1.26 ) | | | 0.55 ( 0.21 ) | | -0.87 ( -2.37 ) |
| Neuralgia | 3 (0.2%) | | 3.01 ( 0.81 - 11.12 ) | | | 3 ( 3.01 ) | | | 2.5 ( 0.84 ) | | 1.32 ( -0.33 ) |
| Herpes zoster | 3 (0.2%) | | 1.18 ( 0.35 - 3.92 ) | | | 1.18 ( 0.07 ) | | | 1.16 ( 0.42 ) | | 0.21 ( -1.34 ) |
| Abdominal pain | 3 (0.2%) | | 1.93 ( 0.55 - 6.73 ) | | | 1.93 ( 1.11 ) | | | 1.77 ( 0.62 ) | | 0.82 ( -0.77 ) |
| Injection site irritation | 3 (0.2%) | | 3.87 ( 1 - 14.98 ) | | | 3.86 ( 4.46 ) | | | 3 ( 0.97 ) | | 1.59 ( -0.1 ) |
| Feeding disorder | 3 (0.2%) | | 3.01 ( 0.81 - 11.12 ) | | | 3 ( 3.01 ) | | | 2.5 ( 0.84 ) | | 1.32 ( -0.33 ) |
| Nervousness | 2 (0.1%) | | 2.58 ( 0.53 - 12.42 ) | | | 2.58 ( 1.5 ) | | | 2.23 ( 0.6 ) | | 1.15 ( -0.75 ) |
| Hypoaesthesia oral | 2 (0.1%) | | 4.51 ( 0.83 - 24.65 ) | | | 4.51 ( 3.64 ) | | | 3.34 ( 0.81 ) | | 1.74 ( -0.25 ) |
| Fear | 2 (0.1%) | | 2.58 ( 0.53 - 12.42 ) | | | 2.58 ( 1.5 ) | | | 2.23 ( 0.6 ) | | 1.15 ( -0.75 ) |
| Spinal pain | 2 (0.1%) | | 9.02 ( 1.27 - 64.11 ) | | | 9.01 ( 7.13 ) | | | 5.01 ( 0.97 ) | | 2.32 ( 0.22 ) |
| Presyncope | 2 (0.1%) | | 1.8 ( 0.39 - 8.24 ) | | | 1.8 ( 0.6 ) | | | 1.67 ( 0.47 ) | | 0.74 ( -1.11 ) |
| Pharyngeal swelling | 2 (0.1%) | | 2 ( 0.43 - 9.28 ) | | | 2 ( 0.82 ) | | | 1.82 ( 0.5 ) | | 0.86 ( -1 ) |
| Ocular hyperaemia | 2 (0.1%) | | 4.51 ( 0.83 - 24.65 ) | | | 4.51 ( 3.64 ) | | | 3.34 ( 0.81 ) | | 1.74 ( -0.25 ) |
| Atrial fibrillation | 2 (0.1%) | | 0.64 ( 0.15 - 2.7 ) | | | 0.64 ( 0.37 ) | | | 0.67 ( 0.2 ) | | -0.58 ( -2.33 ) |
| Feeling of body temperature change | 2 (0.1%) | | 3.01 ( 0.61 - 14.91 ) | | | 3 ( 2.01 ) | | | 2.5 ( 0.66 ) | | 1.32 ( -0.6 ) |
| Abdominal discomfort | 2 (0.1%) | | 0.78 ( 0.18 - 3.33 ) | | | 0.78 ( 0.11 ) | | | 0.8 ( 0.24 ) | | -0.32 ( -2.08 ) |
| Blood pressure increased | 2 (0.1%) | | 0.62 ( 0.15 - 2.61 ) | | | 0.62 ( 0.43 ) | | | 0.65 ( 0.19 ) | | -0.63 ( -2.37 ) |
| Eye pain | 2 (0.1%) | | 2.25 ( 0.48 - 10.63 ) | | | 2.25 ( 1.12 ) | | | 2 ( 0.55 ) | | 1 ( -0.88 ) |
| Swelling of eyelid | 2 (0.1%) | | 3.01 ( 0.61 - 14.91 ) | | | 3 ( 2.01 ) | | | 2.5 ( 0.66 ) | | 1.32 ( -0.6 ) |
| Pollakiuria | 2 (0.1%) | | 2.58 ( 0.53 - 12.42 ) | | | 2.58 ( 1.5 ) | | | 2.23 ( 0.6 ) | | 1.15 ( -0.75 ) |
| Insomnia | 2 (0.1%) | | 0.86 ( 0.2 - 3.66 ) | | | 0.86 ( 0.04 ) | | | 0.87 ( 0.26 ) | | -0.2 ( -1.97 ) |
| Injection site bruising | 2 (0.1%) | | 0.47 ( 0.11 - 1.97 ) | | | 0.47 ( 1.11 ) | | | 0.5 ( 0.15 ) | | -1 ( -2.73 ) |
| Injection site haemorrhage | 2 (0.1%) | | 1.8 ( 0.39 - 8.24 ) | | | 1.8 ( 0.6 ) | | | 1.67 ( 0.47 ) | | 0.74 ( -1.11 ) |
| Rash papular | 2 (0.1%) | | 1.5 ( 0.34 - 6.72 ) | | | 1.5 ( 0.29 ) | | | 1.43 ( 0.41 ) | | 0.52 ( -1.31 ) |
| Covid-19 | 2 (0.1%) | | 0.03 ( 0.01 - 0.11 ) | | | 0.03 ( 66.35 ) | | | 0.03 ( 0.01 ) | | -4.94 ( -6.61 ) |
| Sars-cov-2 test positive | 2 (0.1%) | | 0.22 ( 0.05 - 0.9 ) | | | 0.22 ( 5.33 ) | | | 0.24 ( 0.07 ) | | -2.05 ( -3.75 ) |
| Eye swelling | 2 (0.1%) | | 1.8 ( 0.39 - 8.24 ) | | | 1.8 ( 0.6 ) | | | 1.67 ( 0.47 ) | | 0.74 ( -1.11 ) |
| Head discomfort | 2 (0.1%) | | 1.64 ( 0.36 - 7.4 ) | | | 1.64 ( 0.42 ) | | | 1.54 ( 0.44 ) | | 0.62 ( -1.21 ) |
| Asthma | 2 (0.1%) | | 3.61 ( 0.7 - 18.62 ) | | | 3.61 ( 2.69 ) | | | 2.86 ( 0.72 ) | | 1.52 ( -0.44 ) |
| Retching | 2 (0.1%) | | 4.51 ( 0.83 - 24.65 ) | | | 4.51 ( 3.64 ) | | | 3.34 ( 0.81 ) | | 1.74 ( -0.25 ) |
| Hypertension | 2 (0.1%) | | 0.82 ( 0.19 - 3.49 ) | | | 0.82 ( 0.07 ) | | | 0.83 ( 0.25 ) | | -0.26 ( -2.03 ) |
| Hypersensitivity | 2 (0.1%) | | 0.6 ( 0.14 - 2.51 ) | | | 0.6 ( 0.5 ) | | | 0.63 ( 0.19 ) | | -0.68 ( -2.42 ) |
| Lip swelling | 2 (0.1%) | | 0.9 ( 0.21 - 3.86 ) | | | 0.9 ( 0.02 ) | | | 0.91 ( 0.27 ) | | -0.14 ( -1.91 ) |
| Nasal congestion | 2 (0.1%) | | 0.86 ( 0.2 - 3.66 ) | | | 0.86 ( 0.04 ) | | | 0.87 ( 0.26 ) | | -0.2 ( -1.97 ) |
| Balance disorder | 2 (0.1%) | | 0.62 ( 0.15 - 2.61 ) | | | 0.62 ( 0.43 ) | | | 0.65 ( 0.19 ) | | -0.63 ( -2.37 ) |
| Swollen tongue | 2 (0.1%) | | 3.61 ( 0.7 - 18.62 ) | | | 3.61 ( 2.69 ) | | | 2.86 ( 0.72 ) | | 1.52 ( -0.44 ) |
| Post herpetic neuralgia | 2 (0.1%) | | 18.05 ( 1.64 - 199.16 ) | | | 18.03 ( 10.72 ) | | | 6.68 ( 0.9 ) | | 2.74 ( 0.53 ) |
| Dysgeusia | 2 (0.1%) | | 2 ( 0.43 - 9.28 ) | | | 2 ( 0.82 ) | | | 1.82 ( 0.5 ) | | 0.86 ( -1 ) |
| Inflammation | 2 (0.1%) | | 0.75 ( 0.18 - 3.18 ) | | | 0.75 ( 0.15 ) | | | 0.77 ( 0.23 ) | | -0.38 ( -2.13 ) |
| Visual impairment | 2 (0.1%) | | 1.2 ( 0.27 - 5.26 ) | | | 1.2 ( 0.06 ) | | | 1.18 ( 0.34 ) | | 0.24 ( -1.56 ) |
| Paralysis | 2 (0.1%) | | 4.51 ( 0.83 - 24.65 ) | | | 4.51 ( 3.64 ) | | | 3.34 ( 0.81 ) | | 1.74 ( -0.25 ) |
| Transient ischaemic attack | 2 (0.1%) | | 18.05 ( 1.64 - 199.16 ) | | | 18.03 ( 10.72 ) | | | 6.68 ( 0.9 ) | | 2.74 ( 0.53 ) |
| Burning sensation | 2 (0.1%) | | 0.9 ( 0.21 - 3.86 ) | | | 0.9 ( 0.02 ) | | | 0.91 ( 0.27 ) | | -0.14 ( -1.91 ) |
| Memory impairment | 2 (0.1%) | | 2.25 ( 0.48 - 10.63 ) | | | 2.25 ( 1.12 ) | | | 2 ( 0.55 ) | | 1 ( -0.88 ) |
| Hyperventilation | 1 (0.1%) | | 9.02 ( 0.56 - 144.26 ) | | | 9.01 ( 3.56 ) | | | 5.01 ( 0.49 ) | | 2.32 ( -0.31 ) |
| Hypoacusis | 1 (0.1%) | | 1.29 ( 0.16 - 10.47 ) | | | 1.29 ( 0.06 ) | | | 1.25 ( 0.22 ) | | 0.32 ( -1.93 ) |
| Electric shock sensation | 1 (0.1%) | | 3.01 ( 0.31 - 28.91 ) | | | 3 ( 1 ) | | | 2.5 ( 0.38 ) | | 1.32 ( -1.09 ) |
| Joint range of motion decreased | 1 (0.1%) | | 1.5 ( 0.18 - 12.49 ) | | | 1.5 ( 0.14 ) | | | 1.43 ( 0.24 ) | | 0.52 ( -1.77 ) |
| Renal pain | 1 (0.1%) | | 1.8 ( 0.21 - 15.44 ) | | | 1.8 ( 0.3 ) | | | 1.67 ( 0.28 ) | | 0.74 ( -1.58 ) |
| Palatal swelling | 1 (0.1%) | | 4.51 ( 0.41 - 49.75 ) | | | 4.51 ( 1.82 ) | | | 3.34 ( 0.45 ) | | 1.74 ( -0.76 ) |
| Abdominal pain upper | 1 (0.1%) | | 0.36 ( 0.05 - 2.66 ) | | | 0.36 ( 1.09 ) | | | 0.39 ( 0.07 ) | | -1.38 ( -3.49 ) |
| Neuropathy peripheral | 1 (0.1%) | | 4.51 ( 0.41 - 49.75 ) | | | 4.51 ( 1.82 ) | | | 3.34 ( 0.45 ) | | 1.74 ( -0.76 ) |
| Musculoskeletal disorder | 1 (0.1%) | | 1.5 ( 0.18 - 12.49 ) | | | 1.5 ( 0.14 ) | | | 1.43 ( 0.24 ) | | 0.52 ( -1.77 ) |
| Initial insomnia | 1 (0.1%) | | 4.51 ( 0.41 - 49.75 ) | | | 4.51 ( 1.82 ) | | | 3.34 ( 0.45 ) | | 1.74 ( -0.76 ) |
| Thirst | 1 (0.1%) | | 4.51 ( 0.41 - 49.75 ) | | | 4.51 ( 1.82 ) | | | 3.34 ( 0.45 ) | | 1.74 ( -0.76 ) |
| Flushing | 1 (0.1%) | | 0.6 ( 0.08 - 4.55 ) | | | 0.6 ( 0.25 ) | | | 0.63 ( 0.11 ) | | -0.68 ( -2.83 ) |
| Bone pain | 1 (0.1%) | | 0.9 ( 0.12 - 7.05 ) | | | 0.9 ( 0.01 ) | | | 0.91 ( 0.16 ) | | -0.14 ( -2.34 ) |
| Throat irritation | 1 (0.1%) | | 1 ( 0.13 - 7.91 ) | | | 1 ( 0 ) | | | 1 ( 0.18 ) | | 0 ( -2.22 ) |
| Breast swelling | 1 (0.1%) | | 9.02 ( 0.56 - 144.26 ) | | | 9.01 ( 3.56 ) | | | 5.01 ( 0.49 ) | | 2.32 ( -0.31 ) |
| Hypotension | 1 (0.1%) | | 0.69 ( 0.09 - 5.3 ) | | | 0.69 ( 0.13 ) | | | 0.72 ( 0.13 ) | | -0.48 ( -2.66 ) |
| Amnesia | 1 (0.1%) | | 1.29 ( 0.16 - 10.47 ) | | | 1.29 ( 0.06 ) | | | 1.25 ( 0.22 ) | | 0.32 ( -1.93 ) |
| Cognitive disorder | 1 (0.1%) | | 4.51 ( 0.41 - 49.75 ) | | | 4.51 ( 1.82 ) | | | 3.34 ( 0.45 ) | | 1.74 ( -0.76 ) |
| Staring | 1 (0.1%) | | 4.51 ( 0.41 - 49.75 ) | | | 4.51 ( 1.82 ) | | | 3.34 ( 0.45 ) | | 1.74 ( -0.76 ) |
| Incoherent | 1 (0.1%) | | 4.51 ( 0.41 - 49.75 ) | | | 4.51 ( 1.82 ) | | | 3.34 ( 0.45 ) | | 1.74 ( -0.76 ) |
| Disorientation | 1 (0.1%) | | 1.29 ( 0.16 - 10.47 ) | | | 1.29 ( 0.06 ) | | | 1.25 ( 0.22 ) | | 0.32 ( -1.93 ) |
| Poor quality sleep | 1 (0.1%) | | 2.25 ( 0.25 - 20.18 ) | | | 2.25 ( 0.56 ) | | | 2 ( 0.32 ) | | 1 ( -1.35 ) |
| Vaccination site pain | 1 (0.1%) | | 0.32 ( 0.04 - 2.36 ) | | | 0.32 ( 1.38 ) | | | 0.35 ( 0.07 ) | | -1.53 ( -3.64 ) |
| Heart rate abnormal | 1 (0.1%) | | 9.02 ( 0.56 - 144.26 ) | | | 9.01 ( 3.56 ) | | | 5.01 ( 0.49 ) | | 2.32 ( -0.31 ) |
| Sensory disturbance | 1 (0.1%) | | 1.8 ( 0.21 - 15.44 ) | | | 1.8 ( 0.3 ) | | | 1.67 ( 0.28 ) | | 0.74 ( -1.58 ) |
| Body temperature increased | 1 (0.1%) | | 0.9 ( 0.12 - 7.05 ) | | | 0.9 ( 0.01 ) | | | 0.91 ( 0.16 ) | | -0.14 ( -2.34 ) |
| Oedema peripheral | 1 (0.1%) | | 0.75 ( 0.1 - 5.78 ) | | | 0.75 ( 0.08 ) | | | 0.77 ( 0.14 ) | | -0.38 ( -2.56 ) |
| Depression | 1 (0.1%) | | 4.51 ( 0.41 - 49.75 ) | | | 4.51 ( 1.82 ) | | | 3.34 ( 0.45 ) | | 1.74 ( -0.76 ) |
| Irritability | 1 (0.1%) | | 9.02 ( 0.56 - 144.26 ) | | | 9.01 ( 3.56 ) | | | 5.01 ( 0.49 ) | | 2.32 ( -0.31 ) |
| Muscle swelling | 1 (0.1%) | | 3.01 ( 0.31 - 28.91 ) | | | 3 ( 1 ) | | | 2.5 ( 0.38 ) | | 1.32 ( -1.09 ) |
| Local reaction | 1 (0.1%) | | 9.02 ( 0.56 - 144.26 ) | | | 9.01 ( 3.56 ) | | | 5.01 ( 0.49 ) | | 2.32 ( -0.31 ) |
| Cerebrovascular accident | 1 (0.1%) | | 1.13 ( 0.14 - 9.01 ) | | | 1.13 ( 0.01 ) | | | 1.11 ( 0.2 ) | | 0.15 ( -2.08 ) |
| Seizure | 1 (0.1%) | | 0.53 ( 0.07 - 3.98 ) | | | 0.53 ( 0.39 ) | | | 0.56 ( 0.1 ) | | -0.85 ( -2.99 ) |
| Eye pruritus | 1 (0.1%) | | 3.01 ( 0.31 - 28.91 ) | | | 3 ( 1 ) | | | 2.5 ( 0.38 ) | | 1.32 ( -1.09 ) |
| Chronic obstructive pulmonary disease | 1 (0.1%) | | 4.51 ( 0.41 - 49.75 ) | | | 4.51 ( 1.82 ) | | | 3.34 ( 0.45 ) | | 1.74 ( -0.76 ) |
| Muscle tightness | 1 (0.1%) | | 2.25 ( 0.25 - 20.18 ) | | | 2.25 ( 0.56 ) | | | 2 ( 0.32 ) | | 1 ( -1.35 ) |
| Pain in jaw | 1 (0.1%) | | 0.75 ( 0.1 - 5.78 ) | | | 0.75 ( 0.08 ) | | | 0.77 ( 0.14 ) | | -0.38 ( -2.56 ) |
| Ultrasound doppler abnormal | 1 (0.1%) | | 4.51 ( 0.41 - 49.75 ) | | | 4.51 ( 1.82 ) | | | 3.34 ( 0.45 ) | | 1.74 ( -0.76 ) |
| Pneumonia | 1 (0.1%) | | 0.75 ( 0.1 - 5.78 ) | | | 0.75 ( 0.08 ) | | | 0.77 ( 0.14 ) | | -0.38 ( -2.56 ) |
| Weight decreased | 1 (0.1%) | | 0.82 ( 0.11 - 6.35 ) | | | 0.82 ( 0.04 ) | | | 0.83 ( 0.15 ) | | -0.26 ( -2.45 ) |
| Erythema of eyelid | 1 (0.1%) | | 9.02 ( 0.56 - 144.26 ) | | | 9.01 ( 3.56 ) | | | 5.01 ( 0.49 ) | | 2.32 ( -0.31 ) |
| Hypotonia | 1 (0.1%) | | 2.25 ( 0.25 - 20.18 ) | | | 2.25 ( 0.56 ) | | | 2 ( 0.32 ) | | 1 ( -1.35 ) |
| Heart rate decreased | 1 (0.1%) | | 3.01 ( 0.31 - 28.91 ) | | | 3 ( 1 ) | | | 2.5 ( 0.38 ) | | 1.32 ( -1.09 ) |
| Seizure like phenomena | 1 (0.1%) | | 9.02 ( 0.56 - 144.26 ) | | | 9.01 ( 3.56 ) | | | 5.01 ( 0.49 ) | | 2.32 ( -0.31 ) |
| Exercise tolerance decreased | 1 (0.1%) | | 4.51 ( 0.41 - 49.75 ) | | | 4.51 ( 1.82 ) | | | 3.34 ( 0.45 ) | | 1.74 ( -0.76 ) |
| Dizziness postural | 1 (0.1%) | | 1.29 ( 0.16 - 10.47 ) | | | 1.29 ( 0.06 ) | | | 1.25 ( 0.22 ) | | 0.32 ( -1.93 ) |
| Blood pressure decreased | 1 (0.1%) | | 1.5 ( 0.18 - 12.49 ) | | | 1.5 ( 0.14 ) | | | 1.43 ( 0.24 ) | | 0.52 ( -1.77 ) |
| Chromaturia | 1 (0.1%) | | 9.02 ( 0.56 - 144.26 ) | | | 9.01 ( 3.56 ) | | | 5.01 ( 0.49 ) | | 2.32 ( -0.31 ) |
| Viral infection | 1 (0.1%) | | 9.02 ( 0.56 - 144.26 ) | | | 9.01 ( 3.56 ) | | | 5.01 ( 0.49 ) | | 2.32 ( -0.31 ) |
| Skin texture abnormal | 1 (0.1%) | | 9.02 ( 0.56 - 144.26 ) | | | 9.01 ( 3.56 ) | | | 5.01 ( 0.49 ) | | 2.32 ( -0.31 ) |
| Impaired work ability | 1 (0.1%) | | 0.45 ( 0.06 - 3.36 ) | | | 0.45 ( 0.64 ) | | | 0.48 ( 0.09 ) | | -1.07 ( -3.2 ) |
| Pulmonary pain | 1 (0.1%) | | 9.02 ( 0.56 - 144.26 ) | | | 9.01 ( 3.56 ) | | | 5.01 ( 0.49 ) | | 2.32 ( -0.31 ) |
| Hallucinations, mixed | 1 (0.1%) | | 9.02 ( 0.56 - 144.26 ) | | | 9.01 ( 3.56 ) | | | 5.01 ( 0.49 ) | | 2.32 ( -0.31 ) |
| Rotator cuff syndrome | 1 (0.1%) | | 2.25 ( 0.25 - 20.18 ) | | | 2.25 ( 0.56 ) | | | 2 ( 0.32 ) | | 1 ( -1.35 ) |
| Musculoskeletal pain | 1 (0.1%) | | 1.29 ( 0.16 - 10.47 ) | | | 1.29 ( 0.06 ) | | | 1.25 ( 0.22 ) | | 0.32 ( -1.93 ) |
| Tendon disorder | 1 (0.1%) | | 2.25 ( 0.25 - 20.18 ) | | | 2.25 ( 0.56 ) | | | 2 ( 0.32 ) | | 1 ( -1.35 ) |
| Ultrasound joint abnormal | 1 (0.1%) | | 9.02 ( 0.56 - 144.26 ) | | | 9.01 ( 3.56 ) | | | 5.01 ( 0.49 ) | | 2.32 ( -0.31 ) |
| Hypopnoea | 1 (0.1%) | | 9.02 ( 0.56 - 144.26 ) | | | 9.01 ( 3.56 ) | | | 5.01 ( 0.49 ) | | 2.32 ( -0.31 ) |
| Skin reaction | 1 (0.1%) | | 1.5 ( 0.18 - 12.49 ) | | | 1.5 ( 0.14 ) | | | 1.43 ( 0.24 ) | | 0.52 ( -1.77 ) |
| Facial paralysis | 1 (0.1%) | | 0.9 ( 0.12 - 7.05 ) | | | 0.9 ( 0.01 ) | | | 0.91 ( 0.16 ) | | -0.14 ( -2.34 ) |
| Bell's palsy | 1 (0.1%) | | 0.6 ( 0.08 - 4.55 ) | | | 0.6 ( 0.25 ) | | | 0.63 ( 0.11 ) | | -0.68 ( -2.83 ) |
| Limb mass | 1 (0.1%) | | 4.51 ( 0.41 - 49.75 ) | | | 4.51 ( 1.82 ) | | | 3.34 ( 0.45 ) | | 1.74 ( -0.76 ) |
| Ear discomfort | 1 (0.1%) | | 4.51 ( 0.41 - 49.75 ) | | | 4.51 ( 1.82 ) | | | 3.34 ( 0.45 ) | | 1.74 ( -0.76 ) |
| Axillary pain | 1 (0.1%) | | 0.75 ( 0.1 - 5.78 ) | | | 0.75 ( 0.08 ) | | | 0.77 ( 0.14 ) | | -0.38 ( -2.56 ) |
| Lung disorder | 1 (0.1%) | | 9.02 ( 0.56 - 144.26 ) | | | 9.01 ( 3.56 ) | | | 5.01 ( 0.49 ) | | 2.32 ( -0.31 ) |
| Choking | 1 (0.1%) | | 4.51 ( 0.41 - 49.75 ) | | | 4.51 ( 1.82 ) | | | 3.34 ( 0.45 ) | | 1.74 ( -0.76 ) |
| Dysphagia | 1 (0.1%) | | 0.9 ( 0.12 - 7.05 ) | | | 0.9 ( 0.01 ) | | | 0.91 ( 0.16 ) | | -0.14 ( -2.34 ) |
| Night sweats | 1 (0.1%) | | 0.75 ( 0.1 - 5.78 ) | | | 0.75 ( 0.08 ) | | | 0.77 ( 0.14 ) | | -0.38 ( -2.56 ) |
| Ageusia | 1 (0.1%) | | 0.64 ( 0.08 - 4.9 ) | | | 0.64 ( 0.18 ) | | | 0.67 ( 0.12 ) | | -0.58 ( -2.75 ) |
| Acne | 1 (0.1%) | | 9.02 ( 0.56 - 144.26 ) | | | 9.01 ( 3.56 ) | | | 5.01 ( 0.49 ) | | 2.32 ( -0.31 ) |
| C-reactive protein increased | 1 (0.1%) | | 1.8 ( 0.21 - 15.44 ) | | | 1.8 ( 0.3 ) | | | 1.67 ( 0.28 ) | | 0.74 ( -1.58 ) |
| Giant cell arteritis | 1 (0.1%) | | 4.51 ( 0.41 - 49.75 ) | | | 4.51 ( 1.82 ) | | | 3.34 ( 0.45 ) | | 1.74 ( -0.76 ) |
| Inflammatory marker increased | 1 (0.1%) | | 3.01 ( 0.31 - 28.91 ) | | | 3 ( 1 ) | | | 2.5 ( 0.38 ) | | 1.32 ( -1.09 ) |
| Polymyalgia rheumatica | 1 (0.1%) | | 1.29 ( 0.16 - 10.47 ) | | | 1.29 ( 0.06 ) | | | 1.25 ( 0.22 ) | | 0.32 ( -1.93 ) |
| Red blood cell sedimentation rate increased | 1 (0.1%) | | 4.51 ( 0.41 - 49.75 ) | | | 4.51 ( 1.82 ) | | | 3.34 ( 0.45 ) | | 1.74 ( -0.76 ) |
| Tachycardia | 1 (0.1%) | | 0.75 ( 0.1 - 5.78 ) | | | 0.75 ( 0.08 ) | | | 0.77 ( 0.14 ) | | -0.38 ( -2.56 ) |
| Nightmare | 1 (0.1%) | | 2.25 ( 0.25 - 20.18 ) | | | 2.25 ( 0.56 ) | | | 2 ( 0.32 ) | | 1 ( -1.35 ) |
| Diabetes mellitus | 1 (0.1%) | | 2.25 ( 0.25 - 20.18 ) | | | 2.25 ( 0.56 ) | | | 2 ( 0.32 ) | | 1 ( -1.35 ) |
| Injection site inflammation | 1 (0.1%) | | 0.9 ( 0.12 - 7.05 ) | | | 0.9 ( 0.01 ) | | | 0.91 ( 0.16 ) | | -0.14 ( -2.34 ) |
| Injection site discolouration | 1 (0.1%) | | 0.64 ( 0.08 - 4.9 ) | | | 0.64 ( 0.18 ) | | | 0.67 ( 0.12 ) | | -0.58 ( -2.75 ) |
| Cardiac murmur | 1 (0.1%) | | 9.02 ( 0.56 - 144.26 ) | | | 9.01 ( 3.56 ) | | | 5.01 ( 0.49 ) | | 2.32 ( -0.31 ) |
| Micturition urgency | 1 (0.1%) | | 9.02 ( 0.56 - 144.26 ) | | | 9.01 ( 3.56 ) | | | 5.01 ( 0.49 ) | | 2.32 ( -0.31 ) |
| Restless legs syndrome | 1 (0.1%) | | 9.02 ( 0.56 - 144.26 ) | | | 9.01 ( 3.56 ) | | | 5.01 ( 0.49 ) | | 2.32 ( -0.31 ) |
| Anxiety | 1 (0.1%) | | 0.82 ( 0.11 - 6.35 ) | | | 0.82 ( 0.04 ) | | | 0.83 ( 0.15 ) | | -0.26 ( -2.45 ) |
| Panic attack | 1 (0.1%) | | 9.02 ( 0.56 - 144.26 ) | | | 9.01 ( 3.56 ) | | | 5.01 ( 0.49 ) | | 2.32 ( -0.31 ) |
| White blood cell count decreased | 1 (0.1%) | | 3.01 ( 0.31 - 28.91 ) | | | 3 ( 1 ) | | | 2.5 ( 0.38 ) | | 1.32 ( -1.09 ) |
| Body temperature | 1 (0.1%) | | 0.45 ( 0.06 - 3.36 ) | | | 0.45 ( 0.64 ) | | | 0.48 ( 0.09 ) | | -1.07 ( -3.2 ) |
| Guillain-barre syndrome | 1 (0.1%) | | 1.5 ( 0.18 - 12.49 ) | | | 1.5 ( 0.14 ) | | | 1.43 ( 0.24 ) | | 0.52 ( -1.77 ) |
| Coordination abnormal | 1 (0.1%) | | 9.02 ( 0.56 - 144.26 ) | | | 9.01 ( 3.56 ) | | | 5.01 ( 0.49 ) | | 2.32 ( -0.31 ) |
| Muscle disorder | 1 (0.1%) | | 2.25 ( 0.25 - 20.18 ) | | | 2.25 ( 0.56 ) | | | 2 ( 0.32 ) | | 1 ( -1.35 ) |
| Wheezing | 1 (0.1%) | | 0.6 ( 0.08 - 4.55 ) | | | 0.6 ( 0.25 ) | | | 0.63 ( 0.11 ) | | -0.68 ( -2.83 ) |
| Gout | 1 (0.1%) | | 4.51 ( 0.41 - 49.75 ) | | | 4.51 ( 1.82 ) | | | 3.34 ( 0.45 ) | | 1.74 ( -0.76 ) |
| Unresponsive to stimuli | 1 (0.1%) | | 0.82 ( 0.11 - 6.35 ) | | | 0.82 ( 0.04 ) | | | 0.83 ( 0.15 ) | | -0.26 ( -2.45 ) |
| Injection site urticaria | 1 (0.1%) | | 1.5 ( 0.18 - 12.49 ) | | | 1.5 ( 0.14 ) | | | 1.43 ( 0.24 ) | | 0.52 ( -1.77 ) |
| Drug hypersensitivity | 1 (0.1%) | | 4.51 ( 0.41 - 49.75 ) | | | 4.51 ( 1.82 ) | | | 3.34 ( 0.45 ) | | 1.74 ( -0.76 ) |
| Injection site paraesthesia | 1 (0.1%) | | 4.51 ( 0.41 - 49.75 ) | | | 4.51 ( 1.82 ) | | | 3.34 ( 0.45 ) | | 1.74 ( -0.76 ) |
| Diplegia | 1 (0.1%) | | 4.51 ( 0.41 - 49.75 ) | | | 4.51 ( 1.82 ) | | | 3.34 ( 0.45 ) | | 1.74 ( -0.76 ) |
| Face injury | 1 (0.1%) | | 9.02 ( 0.56 - 144.26 ) | | | 9.01 ( 3.56 ) | | | 5.01 ( 0.49 ) | | 2.32 ( -0.31 ) |
| Troponin | 1 (0.1%) | | 3.01 ( 0.31 - 28.91 ) | | | 3 ( 1 ) | | | 2.5 ( 0.38 ) | | 1.32 ( -1.09 ) |
| Lipase | 1 (0.1%) | | 3.01 ( 0.31 - 28.91 ) | | | 3 ( 1 ) | | | 2.5 ( 0.38 ) | | 1.32 ( -1.09 ) |
| Lymph node pain | 1 (0.1%) | | 3.01 ( 0.31 - 28.91 ) | | | 3 ( 1 ) | | | 2.5 ( 0.38 ) | | 1.32 ( -1.09 ) |
| Limb discomfort | 1 (0.1%) | | 0.45 ( 0.06 - 3.36 ) | | | 0.45 ( 0.64 ) | | | 0.48 ( 0.09 ) | | -1.07 ( -3.2 ) |
| Dysstasia | 1 (0.1%) | | 0.38 ( 0.05 - 2.77 ) | | | 0.38 ( 1 ) | | | 0.4 ( 0.08 ) | | -1.32 ( -3.44 ) |
| Mammogram abnormal | 1 (0.1%) | | 9.02 ( 0.56 - 144.26 ) | | | 9.01 ( 3.56 ) | | | 5.01 ( 0.49 ) | | 2.32 ( -0.31 ) |
| Cold sweat | 1 (0.1%) | | 0.9 ( 0.12 - 7.05 ) | | | 0.9 ( 0.01 ) | | | 0.91 ( 0.16 ) | | -0.14 ( -2.34 ) |
| Throat tightness | 1 (0.1%) | | 1.5 ( 0.18 - 12.49 ) | | | 1.5 ( 0.14 ) | | | 1.43 ( 0.24 ) | | 0.52 ( -1.77 ) |
| Infection | 1 (0.1%) | | 1.29 ( 0.16 - 10.47 ) | | | 1.29 ( 0.06 ) | | | 1.25 ( 0.22 ) | | 0.32 ( -1.93 ) |
| Delirium | 1 (0.1%) | | 1.5 ( 0.18 - 12.49 ) | | | 1.5 ( 0.14 ) | | | 1.43 ( 0.24 ) | | 0.52 ( -1.77 ) |
| Dementia | 1 (0.1%) | | 4.51 ( 0.41 - 49.75 ) | | | 4.51 ( 1.82 ) | | | 3.34 ( 0.45 ) | | 1.74 ( -0.76 ) |
| Alopecia | 1 (0.1%) | | 2.25 ( 0.25 - 20.18 ) | | | 2.25 ( 0.56 ) | | | 2 ( 0.32 ) | | 1 ( -1.35 ) |
| Body height | 1 (0.1%) | | 1.8 ( 0.21 - 15.44 ) | | | 1.8 ( 0.3 ) | | | 1.67 ( 0.28 ) | | 0.74 ( -1.58 ) |
| Bursitis | 1 (0.1%) | | 2.25 ( 0.25 - 20.18 ) | | | 2.25 ( 0.56 ) | | | 2 ( 0.32 ) | | 1 ( -1.35 ) |
| Respiratory disorder | 1 (0.1%) | | 4.51 ( 0.41 - 49.75 ) | | | 4.51 ( 1.82 ) | | | 3.34 ( 0.45 ) | | 1.74 ( -0.76 ) |
| Skin exfoliation | 1 (0.1%) | | 0.9 ( 0.12 - 7.05 ) | | | 0.9 ( 0.01 ) | | | 0.91 ( 0.16 ) | | -0.14 ( -2.34 ) |
| Dry skin | 1 (0.1%) | | 1.13 ( 0.14 - 9.01 ) | | | 1.13 ( 0.01 ) | | | 1.11 ( 0.2 ) | | 0.15 ( -2.08 ) |
| Oral mucosal blistering | 1 (0.1%) | | 1.8 ( 0.21 - 15.44 ) | | | 1.8 ( 0.3 ) | | | 1.67 ( 0.28 ) | | 0.74 ( -1.58 ) |
| Injection site scab | 1 (0.1%) | | 3.01 ( 0.31 - 28.91 ) | | | 3 ( 1 ) | | | 2.5 ( 0.38 ) | | 1.32 ( -1.09 ) |
| Flatulence | 1 (0.1%) | | 1.8 ( 0.21 - 15.44 ) | | | 1.8 ( 0.3 ) | | | 1.67 ( 0.28 ) | | 0.74 ( -1.58 ) |
| Constipation | 1 (0.1%) | | 2.25 ( 0.25 - 20.18 ) | | | 2.25 ( 0.56 ) | | | 2 ( 0.32 ) | | 1 ( -1.35 ) |
| Lichen planus | 1 (0.1%) | | 4.51 ( 0.41 - 49.75 ) | | | 4.51 ( 1.82 ) | | | 3.34 ( 0.45 ) | | 1.74 ( -0.76 ) |
| Mouth swelling | 1 (0.1%) | | 3.01 ( 0.31 - 28.91 ) | | | 3 ( 1 ) | | | 2.5 ( 0.38 ) | | 1.32 ( -1.09 ) |
| Mass | 1 (0.1%) | | 1.5 ( 0.18 - 12.49 ) | | | 1.5 ( 0.14 ) | | | 1.43 ( 0.24 ) | | 0.52 ( -1.77 ) |
| **RSV + Influenza Vaccines** | | | | | | | | | | | |
| **PT** | **N(%)** | | | **ROR(95%Cl)** | | | **PRR(X^2^)** | | **EBGM(EBGM05)** | | **IC(IC025)** |
| Pain in extremity | 63 (3.4%) | | | 1.41 ( 1.07 - 1.85 ) | | | 1.39 ( 6.03 ) | | 1.33 ( 1.06 ) | | 0.41 ( 0.02 ) |
| Pain | 63 (3.4%) | | | 1.43 ( 1.08 - 1.88 ) | | | 1.41 ( 6.48 ) | | 1.34 ( 1.07 ) | | 0.43 ( 0.03 ) |
| Erythema | 49 (2.7%) | | | 1.83 ( 1.33 - 2.51 ) | | | 1.8 ( 14.26 ) | | 1.64 ( 1.26 ) | | 0.72 ( 0.27 ) |
| Injection site swelling | 38 (2.1%) | | | 1.6 ( 1.12 - 2.28 ) | | | 1.58 ( 6.81 ) | | 1.48 ( 1.1 ) | | 0.56 ( 0.06 ) |
| Peripheral swelling | 35 (1.9%) | | | 1.77 ( 1.22 - 2.57 ) | | | 1.75 ( 9.22 ) | | 1.61 ( 1.18 ) | | 0.68 ( 0.15 ) |
| Vomiting | 25 (1.4%) | | | 1.74 ( 1.12 - 2.71 ) | | | 1.73 ( 6.31 ) | | 1.59 ( 1.1 ) | | 0.67 ( 0.05 ) |
| Injection site warmth | 23 (1.3%) | | | 1.67 ( 1.06 - 2.63 ) | | | 1.66 ( 4.93 ) | | 1.54 ( 1.05 ) | | 0.62 ( -0.03 ) |
| Sleep disorder | 17 (0.9%) | | | 2.2 ( 1.28 - 3.8 ) | | | 2.19 ( 8.47 ) | | 1.91 ( 1.21 ) | | 0.94 ( 0.18 ) |
| Back pain | 16 (0.9%) | | | 2.23 ( 1.27 - 3.91 ) | | | 2.22 ( 8.23 ) | | 1.93 ( 1.21 ) | | 0.95 ( 0.17 ) |
| Contusion | 16 (0.9%) | | | 3.05 ( 1.7 - 5.49 ) | | | 3.04 ( 15.44 ) | | 2.43 ( 1.49 ) | | 1.28 ( 0.48 ) |
| Tremor | 15 (0.8%) | | | 1.29 ( 0.74 - 2.24 ) | | | 1.29 ( 0.82 ) | | 1.24 ( 0.78 ) | | 0.32 ( -0.46 ) |
| Mobility decreased | 14 (0.8%) | | | 1.01 ( 0.58 - 1.77 ) | | | 1.01 ( 0 ) | | 1.01 ( 0.63 ) | | 0.01 ( -0.78 ) |
| Skin warm | 14 (0.8%) | | | 1.41 ( 0.79 - 2.5 ) | | | 1.4 ( 1.36 ) | | 1.34 ( 0.83 ) | | 0.42 ( -0.39 ) |
| Condition aggravated | 13 (0.7%) | | | 1.52 ( 0.83 - 2.76 ) | | | 1.51 ( 1.87 ) | | 1.42 ( 0.86 ) | | 0.51 ( -0.33 ) |
| Urticaria | 13 (0.7%) | | | 0.91 ( 0.51 - 1.62 ) | | | 0.91 ( 0.1 ) | | 0.92 ( 0.57 ) | | -0.12 ( -0.94 ) |
| Fall | 13 (0.7%) | | | 1.81 ( 0.98 - 3.33 ) | | | 1.8 ( 3.74 ) | | 1.64 ( 0.99 ) | | 0.72 ( -0.13 ) |
| Injection site pruritus | 13 (0.7%) | | | 1.22 ( 0.68 - 2.2 ) | | | 1.22 ( 0.44 ) | | 1.19 ( 0.72 ) | | 0.25 ( -0.58 ) |
| Rash erythematous | 12 (0.7%) | | | 1.55 ( 0.83 - 2.9 ) | | | 1.55 ( 1.91 ) | | 1.45 ( 0.86 ) | | 0.54 ( -0.34 ) |
| Injection site rash | 12 (0.7%) | | | 1.77 ( 0.94 - 3.34 ) | | | 1.77 ( 3.22 ) | | 1.62 ( 0.95 ) | | 0.69 ( -0.19 ) |
| Swelling | 11 (0.6%) | | | 1.15 ( 0.61 - 2.18 ) | | | 1.15 ( 0.19 ) | | 1.13 ( 0.66 ) | | 0.18 ( -0.72 ) |
| Rash pruritic | 11 (0.6%) | | | 1.81 ( 0.93 - 3.51 ) | | | 1.8 ( 3.16 ) | | 1.64 ( 0.94 ) | | 0.72 ( -0.2 ) |
| Gait disturbance | 11 (0.6%) | | | 1.37 ( 0.72 - 2.62 ) | | | 1.37 ( 0.92 ) | | 1.31 ( 0.76 ) | | 0.39 ( -0.51 ) |
| Feeling abnormal | 11 (0.6%) | | | 1.42 ( 0.74 - 2.71 ) | | | 1.42 ( 1.13 ) | | 1.35 ( 0.78 ) | | 0.43 ( -0.47 ) |
| Influenza like illness | 11 (0.6%) | | | 1.5 ( 0.78 - 2.88 ) | | | 1.5 ( 1.51 ) | | 1.41 ( 0.82 ) | | 0.5 ( -0.41 ) |
| Syncope | 10 (0.5%) | | | 1.27 ( 0.65 - 2.49 ) | | | 1.27 ( 0.48 ) | | 1.23 ( 0.7 ) | | 0.29 ( -0.65 ) |
| Muscular weakness | 10 (0.5%) | | | 1.15 ( 0.59 - 2.24 ) | | | 1.14 ( 0.16 ) | | 1.13 ( 0.64 ) | | 0.17 ( -0.76 ) |
| Injection site bruising | 10 (0.5%) | | | 2.41 ( 1.18 - 4.94 ) | | | 2.4 ( 6.17 ) | | 2.05 ( 1.13 ) | | 1.04 ( 0.06 ) |
| Oropharyngeal pain | 9 (0.5%) | | | 1.12 ( 0.55 - 2.26 ) | | | 1.12 ( 0.1 ) | | 1.1 ( 0.61 ) | | 0.14 ( -0.84 ) |
| Gait inability | 9 (0.5%) | | | 4.65 ( 2.01 - 10.77 ) | | | 4.64 ( 15.65 ) | | 3.21 ( 1.59 ) | | 1.68 ( 0.6 ) |
| Injected limb mobility decreased | 9 (0.5%) | | | 1.1 ( 0.54 - 2.22 ) | | | 1.1 ( 0.07 ) | | 1.09 ( 0.6 ) | | 0.12 ( -0.86 ) |
| Abdominal pain upper | 8 (0.4%) | | | 3.22 ( 1.4 - 7.41 ) | | | 3.21 ( 8.42 ) | | 2.53 ( 1.26 ) | | 1.34 ( 0.23 ) |
| Loss of consciousness | 8 (0.4%) | | | 1.38 ( 0.64 - 2.93 ) | | | 1.37 ( 0.69 ) | | 1.31 ( 0.7 ) | | 0.39 ( -0.65 ) |
| Injection site mass | 8 (0.4%) | | | 4.45 ( 1.84 - 10.76 ) | | | 4.44 ( 13.21 ) | | 3.13 ( 1.5 ) | | 1.65 ( 0.5 ) |
| Hyperhidrosis | 8 (0.4%) | | | 0.89 ( 0.42 - 1.85 ) | | | 0.89 ( 0.1 ) | | 0.9 ( 0.49 ) | | -0.15 ( -1.17 ) |
| Palpitations | 8 (0.4%) | | | 1.48 ( 0.69 - 3.18 ) | | | 1.48 ( 1.03 ) | | 1.4 ( 0.74 ) | | 0.48 ( -0.57 ) |
| Vision blurred | 7 (0.4%) | | | 2.11 ( 0.91 - 4.9 ) | | | 2.1 ( 3.14 ) | | 1.85 ( 0.92 ) | | 0.89 ( -0.25 ) |
| Paraesthesia | 7 (0.4%) | | | 1.03 ( 0.47 - 2.28 ) | | | 1.03 ( 0.01 ) | | 1.03 ( 0.53 ) | | 0.04 ( -1.05 ) |
| Feeling hot | 7 (0.4%) | | | 1.4 ( 0.62 - 3.16 ) | | | 1.4 ( 0.68 ) | | 1.34 ( 0.68 ) | | 0.42 ( -0.69 ) |
| Decreased appetite | 7 (0.4%) | | | 0.83 ( 0.38 - 1.81 ) | | | 0.83 ( 0.23 ) | | 0.85 ( 0.44 ) | | -0.24 ( -1.32 ) |
| Tenderness | 7 (0.4%) | | | 1.33 ( 0.59 - 2.98 ) | | | 1.33 ( 0.48 ) | | 1.28 ( 0.65 ) | | 0.35 ( -0.75 ) |
| Chest discomfort | 7 (0.4%) | | | 1.37 ( 0.61 - 3.07 ) | | | 1.36 ( 0.57 ) | | 1.31 ( 0.66 ) | | 0.39 ( -0.72 ) |
| Respiratory tract congestion | 6 (0.3%) | | | 1.49 ( 0.62 - 3.6 ) | | | 1.49 ( 0.81 ) | | 1.41 ( 0.67 ) | | 0.49 ( -0.7 ) |
| Joint swelling | 6 (0.3%) | | | 2.41 ( 0.95 - 6.08 ) | | | 2.4 ( 3.7 ) | | 2.05 ( 0.95 ) | | 1.04 ( -0.2 ) |
| Neck pain | 5 (0.3%) | | | 0.9 ( 0.36 - 2.29 ) | | | 0.9 ( 0.05 ) | | 0.91 ( 0.42 ) | | -0.13 ( -1.39 ) |
| Discomfort | 5 (0.3%) | | | 1.9 ( 0.71 - 5.1 ) | | | 1.9 ( 1.68 ) | | 1.71 ( 0.75 ) | | 0.77 ( -0.54 ) |
| Lethargy | 5 (0.3%) | | | 0.97 ( 0.38 - 2.48 ) | | | 0.97 ( 0 ) | | 0.98 ( 0.45 ) | | -0.03 ( -1.29 ) |
| Injection site induration | 5 (0.3%) | | | 2.26 ( 0.83 - 6.17 ) | | | 2.25 ( 2.66 ) | | 1.96 ( 0.84 ) | | 0.97 ( -0.36 ) |
| Swelling face | 5 (0.3%) | | | 1.81 ( 0.68 - 4.82 ) | | | 1.8 ( 1.43 ) | | 1.64 ( 0.72 ) | | 0.72 ( -0.59 ) |
| Feeling cold | 5 (0.3%) | | | 1.34 ( 0.51 - 3.48 ) | | | 1.34 ( 0.36 ) | | 1.28 ( 0.58 ) | | 0.36 ( -0.92 ) |
| Chest pain | 5 (0.3%) | | | 0.68 ( 0.27 - 1.7 ) | | | 0.68 ( 0.69 ) | | 0.71 ( 0.33 ) | | -0.5 ( -1.74 ) |
| Muscle spasms | 5 (0.3%) | | | 1.29 ( 0.5 - 3.34 ) | | | 1.29 ( 0.27 ) | | 1.24 ( 0.56 ) | | 0.32 ( -0.96 ) |
| Hypoaesthesia | 4 (0.2%) | | | 0.64 ( 0.23 - 1.78 ) | | | 0.64 ( 0.74 ) | | 0.67 ( 0.28 ) | | -0.58 ( -1.93 ) |
| Burning sensation | 4 (0.2%) | | | 1.6 ( 0.54 - 4.75 ) | | | 1.6 ( 0.74 ) | | 1.49 ( 0.6 ) | | 0.58 ( -0.85 ) |
| Blister | 4 (0.2%) | | | 1.7 ( 0.57 - 5.05 ) | | | 1.7 ( 0.93 ) | | 1.56 ( 0.63 ) | | 0.65 ( -0.79 ) |
| Nasal congestion | 4 (0.2%) | | | 1.52 ( 0.52 - 4.47 ) | | | 1.52 ( 0.59 ) | | 1.43 ( 0.58 ) | | 0.51 ( -0.91 ) |
| Injection site vesicles | 4 (0.2%) | | | 5.78 ( 1.55 - 21.55 ) | | | 5.77 ( 8.77 ) | | 3.65 ( 1.21 ) | | 1.87 ( 0.29 ) |
| Inflammation | 4 (0.2%) | | | 1.31 ( 0.45 - 3.81 ) | | | 1.31 ( 0.25 ) | | 1.26 ( 0.52 ) | | 0.34 ( -1.07 ) |
| Musculoskeletal stiffness | 4 (0.2%) | | | 0.67 ( 0.24 - 1.87 ) | | | 0.67 ( 0.59 ) | | 0.7 ( 0.3 ) | | -0.52 ( -1.87 ) |
| Fear | 4 (0.2%) | | | 5.78 ( 1.55 - 21.55 ) | | | 5.77 ( 8.77 ) | | 3.65 ( 1.21 ) | | 1.87 ( 0.29 ) |
| Productive cough | 4 (0.2%) | | | 2.41 ( 0.78 - 7.47 ) | | | 2.4 ( 2.46 ) | | 2.05 ( 0.8 ) | | 1.04 ( -0.43 ) |
| Rhinorrhoea | 4 (0.2%) | | | 0.8 ( 0.28 - 2.25 ) | | | 0.8 ( 0.18 ) | | 0.82 ( 0.35 ) | | -0.28 ( -1.65 ) |
| Cellulitis | 4 (0.2%) | | | 1.37 ( 0.47 - 4.01 ) | | | 1.37 ( 0.34 ) | | 1.31 ( 0.54 ) | | 0.39 ( -1.02 ) |
| Swelling of eyelid | 3 (0.2%) | | | 4.33 ( 1.03 - 18.15 ) | | | 4.33 ( 4.8 ) | | 3.08 ( 0.93 ) | | 1.62 ( -0.11 ) |
| Limb discomfort | 3 (0.2%) | | | 1.2 ( 0.35 - 4.09 ) | | | 1.2 ( 0.09 ) | | 1.17 ( 0.42 ) | | 0.23 ( -1.34 ) |
| Oral mucosal blistering | 3 (0.2%) | | | 7.22 ( 1.46 - 35.81 ) | | | 7.21 ( 8.03 ) | | 4.11 ( 1.08 ) | | 2.04 ( 0.23 ) |
| Body temperature increased | 3 (0.2%) | | | 2.71 ( 0.72 - 10.22 ) | | | 2.7 ( 2.35 ) | | 2.24 ( 0.74 ) | | 1.16 ( -0.5 ) |
| Somnolence | 3 (0.2%) | | | 1.03 ( 0.31 - 3.46 ) | | | 1.03 ( 0 ) | | 1.03 ( 0.37 ) | | 0.04 ( -1.52 ) |
| Joint stiffness | 3 (0.2%) | | | 3.61 ( 0.9 - 14.45 ) | | | 3.61 ( 3.77 ) | | 2.74 ( 0.86 ) | | 1.45 ( -0.25 ) |
| Night sweats | 3 (0.2%) | | | 2.17 ( 0.6 - 7.88 ) | | | 2.16 ( 1.45 ) | | 1.9 ( 0.64 ) | | 0.92 ( -0.71 ) |
| Brain fog | 3 (0.2%) | | | 0.83 ( 0.25 - 2.75 ) | | | 0.83 ( 0.09 ) | | 0.85 ( 0.31 ) | | -0.24 ( -1.77 ) |
| Skin exfoliation | 3 (0.2%) | | | 2.71 ( 0.72 - 10.22 ) | | | 2.7 ( 2.35 ) | | 2.24 ( 0.74 ) | | 1.16 ( -0.5 ) |
| Neuralgia | 3 (0.2%) | | | 2.41 ( 0.65 - 8.9 ) | | | 2.4 ( 1.85 ) | | 2.05 ( 0.69 ) | | 1.04 ( -0.61 ) |
| Seizure | 3 (0.2%) | | | 1.44 ( 0.42 - 4.99 ) | | | 1.44 ( 0.34 ) | | 1.37 ( 0.48 ) | | 0.45 ( -1.14 ) |
| Confusional state | 3 (0.2%) | | | 0.68 ( 0.21 - 2.21 ) | | | 0.68 ( 0.43 ) | | 0.7 ( 0.26 ) | | -0.51 ( -2.03 ) |
| Muscle swelling | 3 (0.2%) | | | 21.67 ( 2.25 - 208.46 ) | | | 21.64 ( 14.77 ) | | 6.16 ( 0.93 ) | | 2.62 ( 0.69 ) |
| Injection site nodule | 3 (0.2%) | | | 2.71 ( 0.72 - 10.22 ) | | | 2.7 ( 2.35 ) | | 2.24 ( 0.74 ) | | 1.16 ( -0.5 ) |
| Atrial fibrillation | 3 (0.2%) | | | 0.8 ( 0.24 - 2.64 ) | | | 0.8 ( 0.13 ) | | 0.82 ( 0.3 ) | | -0.28 ( -1.82 ) |
| Wheezing | 3 (0.2%) | | | 1.67 ( 0.47 - 5.85 ) | | | 1.66 ( 0.65 ) | | 1.54 ( 0.54 ) | | 0.62 ( -0.98 ) |
| Nasopharyngitis | 3 (0.2%) | | | 0.75 ( 0.23 - 2.45 ) | | | 0.75 ( 0.24 ) | | 0.77 ( 0.28 ) | | -0.38 ( -1.91 ) |
| Memory impairment | 3 (0.2%) | | | 3.09 ( 0.8 - 11.98 ) | | | 3.09 ( 2.97 ) | | 2.46 ( 0.79 ) | | 1.3 ( -0.38 ) |
| Presyncope | 2 (0.1%) | | | 1.44 ( 0.32 - 6.59 ) | | | 1.44 ( 0.23 ) | | 1.37 ( 0.38 ) | | 0.45 ( -1.4 ) |
| Chronic obstructive pulmonary disease | 2 (0.1%) | | | 14.44 ( 1.31 - 159.33 ) | | | 14.43 ( 8.33 ) | | 5.48 ( 0.73 ) | | 2.45 ( 0.25 ) |
| Metabolic function test | 2 (0.1%) | | | 1.6 ( 0.35 - 7.43 ) | | | 1.6 ( 0.37 ) | | 1.49 ( 0.41 ) | | 0.58 ( -1.28 ) |
| Scab | 2 (0.1%) | | | 4.81 ( 0.8 - 28.82 ) | | | 4.81 ( 3.62 ) | | 3.29 ( 0.73 ) | | 1.72 ( -0.33 ) |
| Amnesia | 2 (0.1%) | | | 2.41 ( 0.49 - 11.93 ) | | | 2.4 ( 1.23 ) | | 2.05 ( 0.54 ) | | 1.04 ( -0.89 ) |
| Visual impairment | 2 (0.1%) | | | 0.96 ( 0.22 - 4.21 ) | | | 0.96 ( 0 ) | | 0.97 ( 0.28 ) | | -0.05 ( -1.85 ) |
| Skin discolouration | 2 (0.1%) | | | 1.31 ( 0.29 - 5.92 ) | | | 1.31 ( 0.13 ) | | 1.26 ( 0.36 ) | | 0.34 ( -1.5 ) |
| Middle insomnia | 2 (0.1%) | | | 14.44 ( 1.31 - 159.33 ) | | | 14.43 ( 8.33 ) | | 5.48 ( 0.73 ) | | 2.45 ( 0.25 ) |
| Balance disorder | 2 (0.1%) | | | 0.5 ( 0.12 - 2.08 ) | | | 0.5 ( 0.95 ) | | 0.53 ( 0.16 ) | | -0.92 ( -2.66 ) |
| Induration | 2 (0.1%) | | | 1.6 ( 0.35 - 7.43 ) | | | 1.6 ( 0.37 ) | | 1.49 ( 0.41 ) | | 0.58 ( -1.28 ) |
| Lip swelling | 2 (0.1%) | | | 0.72 ( 0.17 - 3.09 ) | | | 0.72 ( 0.2 ) | | 0.75 ( 0.22 ) | | -0.42 ( -2.19 ) |
| Insomnia | 2 (0.1%) | | | 0.69 ( 0.16 - 2.93 ) | | | 0.69 ( 0.26 ) | | 0.71 ( 0.21 ) | | -0.49 ( -2.25 ) |
| Vaccination site pain | 2 (0.1%) | | | 0.53 ( 0.13 - 2.25 ) | | | 0.53 ( 0.76 ) | | 0.57 ( 0.17 ) | | -0.82 ( -2.57 ) |
| Electric shock sensation | 2 (0.1%) | | | 7.22 ( 1.02 - 51.28 ) | | | 7.21 ( 5.35 ) | | 4.11 ( 0.8 ) | | 2.04 ( -0.07 ) |
| Joint range of motion decreased | 2 (0.1%) | | | 2.89 ( 0.56 - 14.89 ) | | | 2.89 ( 1.76 ) | | 2.35 ( 0.59 ) | | 1.23 ( -0.72 ) |
| Urinary incontinence | 2 (0.1%) | | | 1.6 ( 0.35 - 7.43 ) | | | 1.6 ( 0.37 ) | | 1.49 ( 0.41 ) | | 0.58 ( -1.28 ) |
| Platelet count decreased | 2 (0.1%) | | | 2.41 ( 0.49 - 11.93 ) | | | 2.4 ( 1.23 ) | | 2.05 ( 0.54 ) | | 1.04 ( -0.89 ) |
| Blood glucose increased | 2 (0.1%) | | | 2.06 ( 0.43 - 9.93 ) | | | 2.06 ( 0.85 ) | | 1.83 ( 0.49 ) | | 0.87 ( -1.03 ) |
| Thirst | 2 (0.1%) | | | 14.44 ( 1.31 - 159.33 ) | | | 14.43 ( 8.33 ) | | 5.48 ( 0.73 ) | | 2.45 ( 0.25 ) |
| Constipation | 2 (0.1%) | | | 4.81 ( 0.8 - 28.82 ) | | | 4.81 ( 3.62 ) | | 3.29 ( 0.73 ) | | 1.72 ( -0.33 ) |
| Dizziness postural | 2 (0.1%) | | | 2.41 ( 0.49 - 11.93 ) | | | 2.4 ( 1.23 ) | | 2.05 ( 0.54 ) | | 1.04 ( -0.89 ) |
| Gastrointestinal disorder | 2 (0.1%) | | | 7.22 ( 1.02 - 51.28 ) | | | 7.21 ( 5.35 ) | | 4.11 ( 0.8 ) | | 2.04 ( -0.07 ) |
| Injection site paraesthesia | 2 (0.1%) | | | 14.44 ( 1.31 - 159.33 ) | | | 14.43 ( 8.33 ) | | 5.48 ( 0.73 ) | | 2.45 ( 0.25 ) |
| Heart rate irregular | 2 (0.1%) | | | 2.41 ( 0.49 - 11.93 ) | | | 2.4 ( 1.23 ) | | 2.05 ( 0.54 ) | | 1.04 ( -0.89 ) |
| Ocular hyperaemia | 2 (0.1%) | | | 3.61 ( 0.66 - 19.72 ) | | | 3.61 ( 2.51 ) | | 2.74 ( 0.66 ) | | 1.45 ( -0.54 ) |
| Dry skin | 2 (0.1%) | | | 2.06 ( 0.43 - 9.93 ) | | | 2.06 ( 0.85 ) | | 1.83 ( 0.49 ) | | 0.87 ( -1.03 ) |
| Blood pressure decreased | 2 (0.1%) | | | 2.89 ( 0.56 - 14.89 ) | | | 2.89 ( 1.76 ) | | 2.35 ( 0.59 ) | | 1.23 ( -0.72 ) |
| Anxiety | 2 (0.1%) | | | 1.44 ( 0.32 - 6.59 ) | | | 1.44 ( 0.23 ) | | 1.37 ( 0.38 ) | | 0.45 ( -1.4 ) |
| Bone pain | 2 (0.1%) | | | 1.6 ( 0.35 - 7.43 ) | | | 1.6 ( 0.37 ) | | 1.49 ( 0.41 ) | | 0.58 ( -1.28 ) |
| Dysphonia | 2 (0.1%) | | | 1.2 ( 0.27 - 5.38 ) | | | 1.2 ( 0.06 ) | | 1.17 ( 0.34 ) | | 0.23 ( -1.6 ) |
| Throat irritation | 2 (0.1%) | | | 1.8 ( 0.38 - 8.5 ) | | | 1.8 ( 0.57 ) | | 1.64 ( 0.45 ) | | 0.72 ( -1.16 ) |
| Blood pressure increased | 2 (0.1%) | | | 0.5 ( 0.12 - 2.08 ) | | | 0.5 ( 0.95 ) | | 0.53 ( 0.16 ) | | -0.92 ( -2.66 ) |
| Lymphadenopathy | 2 (0.1%) | | | 0.76 ( 0.18 - 3.26 ) | | | 0.76 ( 0.14 ) | | 0.78 ( 0.23 ) | | -0.35 ( -2.13 ) |
| Injection site haemorrhage | 2 (0.1%) | | | 1.44 ( 0.32 - 6.59 ) | | | 1.44 ( 0.23 ) | | 1.37 ( 0.38 ) | | 0.45 ( -1.4 ) |
| Asthma | 2 (0.1%) | | | 2.89 ( 0.56 - 14.89 ) | | | 2.89 ( 1.76 ) | | 2.35 ( 0.59 ) | | 1.23 ( -0.72 ) |
| Retching | 2 (0.1%) | | | 3.61 ( 0.66 - 19.72 ) | | | 3.61 ( 2.51 ) | | 2.74 ( 0.66 ) | | 1.45 ( -0.54 ) |
| Heart rate increased | 2 (0.1%) | | | 0.44 ( 0.1 - 1.82 ) | | | 0.44 ( 1.37 ) | | 0.47 ( 0.14 ) | | -1.09 ( -2.83 ) |
| Hypersensitivity | 2 (0.1%) | | | 0.48 ( 0.11 - 2.01 ) | | | 0.48 ( 1.05 ) | | 0.51 ( 0.15 ) | | -0.96 ( -2.7 ) |
| Influenza | 2 (0.1%) | | | 1.44 ( 0.32 - 6.59 ) | | | 1.44 ( 0.23 ) | | 1.37 ( 0.38 ) | | 0.45 ( -1.4 ) |
| Tinnitus | 2 (0.1%) | | | 0.55 ( 0.13 - 2.34 ) | | | 0.55 ( 0.66 ) | | 0.59 ( 0.18 ) | | -0.77 ( -2.52 ) |
| Polymyalgia rheumatica | 2 (0.1%) | | | 2.41 ( 0.49 - 11.93 ) | | | 2.4 ( 1.23 ) | | 2.05 ( 0.54 ) | | 1.04 ( -0.89 ) |
| C-reactive protein increased | 2 (0.1%) | | | 3.61 ( 0.66 - 19.72 ) | | | 3.61 ( 2.51 ) | | 2.74 ( 0.66 ) | | 1.45 ( -0.54 ) |
| Musculoskeletal pain | 2 (0.1%) | | | 2.41 ( 0.49 - 11.93 ) | | | 2.4 ( 1.23 ) | | 2.05 ( 0.54 ) | | 1.04 ( -0.89 ) |
| Dry mouth | 2 (0.1%) | | | 7.22 ( 1.02 - 51.28 ) | | | 7.21 ( 5.35 ) | | 4.11 ( 0.8 ) | | 2.04 ( -0.07 ) |
| Transient ischaemic attack | 2 (0.1%) | | | 14.44 ( 1.31 - 159.33 ) | | | 14.43 ( 8.33 ) | | 5.48 ( 0.73 ) | | 2.45 ( 0.25 ) |
| Cerebrovascular accident | 2 (0.1%) | | | 2.06 ( 0.43 - 9.93 ) | | | 2.06 ( 0.85 ) | | 1.83 ( 0.49 ) | | 0.87 ( -1.03 ) |
| Feeding disorder | 2 (0.1%) | | | 1.44 ( 0.32 - 6.59 ) | | | 1.44 ( 0.23 ) | | 1.37 ( 0.38 ) | | 0.45 ( -1.4 ) |
| Vertigo | 2 (0.1%) | | | 0.38 ( 0.09 - 1.57 ) | | | 0.38 ( 1.93 ) | | 0.41 ( 0.12 ) | | -1.28 ( -3.01 ) |
| Odynophagia | 1 (0.1%) | | | 1.8 ( 0.2 - 16.15 ) | | | 1.8 ( 0.29 ) | | 1.64 ( 0.26 ) | | 0.72 ( -1.64 ) |
| Muscle tightness | 1 (0.1%) | | | 1.8 ( 0.2 - 16.15 ) | | | 1.8 ( 0.29 ) | | 1.64 ( 0.26 ) | | 0.72 ( -1.64 ) |
| Ear pruritus | 1 (0.1%) | | | 7.22 ( 0.45 - 115.42 ) | | | 7.21 ( 2.68 ) | | 4.11 ( 0.4 ) | | 2.04 ( -0.6 ) |
| Eyelids pruritus | 1 (0.1%) | | | 7.22 ( 0.45 - 115.42 ) | | | 7.21 ( 2.68 ) | | 4.11 ( 0.4 ) | | 2.04 ( -0.6 ) |
| Arthritis | 1 (0.1%) | | | 0.55 ( 0.07 - 4.24 ) | | | 0.55 ( 0.33 ) | | 0.59 ( 0.11 ) | | -0.77 ( -2.94 ) |
| Arthropathy | 1 (0.1%) | | | 2.4 ( 0.25 - 23.13 ) | | | 2.4 ( 0.62 ) | | 2.05 ( 0.31 ) | | 1.04 ( -1.38 ) |
| Mouth haemorrhage | 1 (0.1%) | | | 3.61 ( 0.33 - 39.81 ) | | | 3.61 ( 1.26 ) | | 2.74 ( 0.37 ) | | 1.45 ( -1.05 ) |
| Hypotonia | 1 (0.1%) | | | 1.8 ( 0.2 - 16.15 ) | | | 1.8 ( 0.29 ) | | 1.64 ( 0.26 ) | | 0.72 ( -1.64 ) |
| Cold sweat | 1 (0.1%) | | | 0.72 ( 0.09 - 5.64 ) | | | 0.72 ( 0.1 ) | | 0.75 ( 0.13 ) | | -0.42 ( -2.63 ) |
| Deep vein thrombosis | 1 (0.1%) | | | 1.8 ( 0.2 - 16.15 ) | | | 1.8 ( 0.29 ) | | 1.64 ( 0.26 ) | | 0.72 ( -1.64 ) |
| Abdominal discomfort | 1 (0.1%) | | | 0.3 ( 0.04 - 2.22 ) | | | 0.3 ( 1.57 ) | | 0.33 ( 0.06 ) | | -1.61 ( -3.72 ) |
| Rash papular | 1 (0.1%) | | | 0.55 ( 0.07 - 4.24 ) | | | 0.55 ( 0.33 ) | | 0.59 ( 0.11 ) | | -0.77 ( -2.94 ) |
| Migraine | 1 (0.1%) | | | 0.36 ( 0.05 - 2.69 ) | | | 0.36 ( 1.08 ) | | 0.39 ( 0.07 ) | | -1.35 ( -3.49 ) |
| Hypertension | 1 (0.1%) | | | 0.31 ( 0.04 - 2.32 ) | | | 0.31 ( 1.44 ) | | 0.34 ( 0.06 ) | | -1.55 ( -3.67 ) |
| Paralysis | 1 (0.1%) | | | 1.44 ( 0.17 - 12.36 ) | | | 1.44 ( 0.11 ) | | 1.37 ( 0.23 ) | | 0.45 ( -1.86 ) |
| Toothache | 1 (0.1%) | | | 1.2 ( 0.14 - 9.99 ) | | | 1.2 ( 0.03 ) | | 1.17 ( 0.2 ) | | 0.23 ( -2.05 ) |
| Vertigo positional | 1 (0.1%) | | | 3.61 ( 0.33 - 39.81 ) | | | 3.61 ( 1.26 ) | | 2.74 ( 0.37 ) | | 1.45 ( -1.05 ) |
| Musculoskeletal chest pain | 1 (0.1%) | | | 0.72 ( 0.09 - 5.64 ) | | | 0.72 ( 0.1 ) | | 0.75 ( 0.13 ) | | -0.42 ( -2.63 ) |
| Hyperventilation | 1 (0.1%) | | | 7.22 ( 0.45 - 115.42 ) | | | 7.21 ( 2.68 ) | | 4.11 ( 0.4 ) | | 2.04 ( -0.6 ) |
| Hypotension | 1 (0.1%) | | | 0.55 ( 0.07 - 4.24 ) | | | 0.55 ( 0.33 ) | | 0.59 ( 0.11 ) | | -0.77 ( -2.94 ) |
| Pulse abnormal | 1 (0.1%) | | | 3.61 ( 0.33 - 39.81 ) | | | 3.61 ( 1.26 ) | | 2.74 ( 0.37 ) | | 1.45 ( -1.05 ) |
| Skin reaction | 1 (0.1%) | | | 1.2 ( 0.14 - 9.99 ) | | | 1.2 ( 0.03 ) | | 1.17 ( 0.2 ) | | 0.23 ( -2.05 ) |
| Bursitis | 1 (0.1%) | | | 1.8 ( 0.2 - 16.15 ) | | | 1.8 ( 0.29 ) | | 1.64 ( 0.26 ) | | 0.72 ( -1.64 ) |
| Magnetic resonance imaging abnormal | 1 (0.1%) | | | 1.8 ( 0.2 - 16.15 ) | | | 1.8 ( 0.29 ) | | 1.64 ( 0.26 ) | | 0.72 ( -1.64 ) |
| Blood pressure systolic increased | 1 (0.1%) | | | 2.4 ( 0.25 - 23.13 ) | | | 2.4 ( 0.62 ) | | 2.05 ( 0.31 ) | | 1.04 ( -1.38 ) |
| Injection site cellulitis | 1 (0.1%) | | | 0.72 ( 0.09 - 5.64 ) | | | 0.72 ( 0.1 ) | | 0.75 ( 0.13 ) | | -0.42 ( -2.63 ) |
| Injection site discharge | 1 (0.1%) | | | 3.61 ( 0.33 - 39.81 ) | | | 3.61 ( 1.26 ) | | 2.74 ( 0.37 ) | | 1.45 ( -1.05 ) |
| Vaccination site rash | 1 (0.1%) | | | 7.22 ( 0.45 - 115.42 ) | | | 7.21 ( 2.68 ) | | 4.11 ( 0.4 ) | | 2.04 ( -0.6 ) |
| Renal pain | 1 (0.1%) | | | 1.44 ( 0.17 - 12.36 ) | | | 1.44 ( 0.11 ) | | 1.37 ( 0.23 ) | | 0.45 ( -1.86 ) |
| Spinal pain | 1 (0.1%) | | | 2.4 ( 0.25 - 23.13 ) | | | 2.4 ( 0.62 ) | | 2.05 ( 0.31 ) | | 1.04 ( -1.38 ) |
| Sensation of foreign body | 1 (0.1%) | | | 7.22 ( 0.45 - 115.42 ) | | | 7.21 ( 2.68 ) | | 4.11 ( 0.4 ) | | 2.04 ( -0.6 ) |
| Eye pruritus | 1 (0.1%) | | | 2.4 ( 0.25 - 23.13 ) | | | 2.4 ( 0.62 ) | | 2.05 ( 0.31 ) | | 1.04 ( -1.38 ) |
| Skin irritation | 1 (0.1%) | | | 1.44 ( 0.17 - 12.36 ) | | | 1.44 ( 0.11 ) | | 1.37 ( 0.23 ) | | 0.45 ( -1.86 ) |
| Blood test abnormal | 1 (0.1%) | | | 0.8 ( 0.1 - 6.33 ) | | | 0.8 ( 0.04 ) | | 0.82 ( 0.15 ) | | -0.28 ( -2.5 ) |
| Hypophagia | 1 (0.1%) | | | 2.4 ( 0.25 - 23.13 ) | | | 2.4 ( 0.62 ) | | 2.05 ( 0.31 ) | | 1.04 ( -1.38 ) |
| Ear discomfort | 1 (0.1%) | | | 3.61 ( 0.33 - 39.81 ) | | | 3.61 ( 1.26 ) | | 2.74 ( 0.37 ) | | 1.45 ( -1.05 ) |
| Facial pain | 1 (0.1%) | | | 1.44 ( 0.17 - 12.36 ) | | | 1.44 ( 0.11 ) | | 1.37 ( 0.23 ) | | 0.45 ( -1.86 ) |
| Hot flush | 1 (0.1%) | | | 3.61 ( 0.33 - 39.81 ) | | | 3.61 ( 1.26 ) | | 2.74 ( 0.37 ) | | 1.45 ( -1.05 ) |
| Petechiae | 1 (0.1%) | | | 2.4 ( 0.25 - 23.13 ) | | | 2.4 ( 0.62 ) | | 2.05 ( 0.31 ) | | 1.04 ( -1.38 ) |
| Papule | 1 (0.1%) | | | 3.61 ( 0.33 - 39.81 ) | | | 3.61 ( 1.26 ) | | 2.74 ( 0.37 ) | | 1.45 ( -1.05 ) |
| Disorientation | 1 (0.1%) | | | 1.03 ( 0.13 - 8.38 ) | | | 1.03 ( 0 ) | | 1.03 ( 0.18 ) | | 0.04 ( -2.22 ) |
| Dysstasia | 1 (0.1%) | | | 0.3 ( 0.04 - 2.22 ) | | | 0.3 ( 1.57 ) | | 0.33 ( 0.06 ) | | -1.61 ( -3.72 ) |
| Blindness | 1 (0.1%) | | | 7.22 ( 0.45 - 115.42 ) | | | 7.21 ( 2.68 ) | | 4.11 ( 0.4 ) | | 2.04 ( -0.6 ) |
| Dehydration | 1 (0.1%) | | | 1.44 ( 0.17 - 12.36 ) | | | 1.44 ( 0.11 ) | | 1.37 ( 0.23 ) | | 0.45 ( -1.86 ) |
| Listless | 1 (0.1%) | | | 3.61 ( 0.33 - 39.81 ) | | | 3.61 ( 1.26 ) | | 2.74 ( 0.37 ) | | 1.45 ( -1.05 ) |
| Crying | 1 (0.1%) | | | 2.4 ( 0.25 - 23.13 ) | | | 2.4 ( 0.62 ) | | 2.05 ( 0.31 ) | | 1.04 ( -1.38 ) |
| Depression | 1 (0.1%) | | | 3.61 ( 0.33 - 39.81 ) | | | 3.61 ( 1.26 ) | | 2.74 ( 0.37 ) | | 1.45 ( -1.05 ) |
| Initial insomnia | 1 (0.1%) | | | 3.61 ( 0.33 - 39.81 ) | | | 3.61 ( 1.26 ) | | 2.74 ( 0.37 ) | | 1.45 ( -1.05 ) |
| Skin swelling | 1 (0.1%) | | | 7.22 ( 0.45 - 115.42 ) | | | 7.21 ( 2.68 ) | | 4.11 ( 0.4 ) | | 2.04 ( -0.6 ) |
| Breast swelling | 1 (0.1%) | | | 7.22 ( 0.45 - 115.42 ) | | | 7.21 ( 2.68 ) | | 4.11 ( 0.4 ) | | 2.04 ( -0.6 ) |
| Vaccination site erythema | 1 (0.1%) | | | 0.45 ( 0.06 - 3.4 ) | | | 0.45 ( 0.63 ) | | 0.48 ( 0.09 ) | | -1.05 ( -3.2 ) |
| Vaccination site pruritus | 1 (0.1%) | | | 1.03 ( 0.13 - 8.38 ) | | | 1.03 ( 0 ) | | 1.03 ( 0.18 ) | | 0.04 ( -2.22 ) |
| Pollakiuria | 1 (0.1%) | | | 0.9 ( 0.11 - 7.21 ) | | | 0.9 ( 0.01 ) | | 0.91 ( 0.16 ) | | -0.13 ( -2.37 ) |
| Dyspnoea exertional | 1 (0.1%) | | | 1.44 ( 0.17 - 12.36 ) | | | 1.44 ( 0.11 ) | | 1.37 ( 0.23 ) | | 0.45 ( -1.86 ) |
| Hypersomnia | 1 (0.1%) | | | 0.38 ( 0.05 - 2.83 ) | | | 0.38 ( 0.96 ) | | 0.41 ( 0.08 ) | | -1.28 ( -3.42 ) |
| Pain in jaw | 1 (0.1%) | | | 0.6 ( 0.08 - 4.62 ) | | | 0.6 ( 0.24 ) | | 0.63 ( 0.11 ) | | -0.66 ( -2.84 ) |
| Ecchymosis | 1 (0.1%) | | | 7.22 ( 0.45 - 115.42 ) | | | 7.21 ( 2.68 ) | | 4.11 ( 0.4 ) | | 2.04 ( -0.6 ) |
| Injection site inflammation | 1 (0.1%) | | | 0.72 ( 0.09 - 5.64 ) | | | 0.72 ( 0.1 ) | | 0.75 ( 0.13 ) | | -0.42 ( -2.63 ) |
| Oedema peripheral | 1 (0.1%) | | | 0.6 ( 0.08 - 4.62 ) | | | 0.6 ( 0.24 ) | | 0.63 ( 0.11 ) | | -0.66 ( -2.84 ) |
| Oral herpes | 1 (0.1%) | | | 2.4 ( 0.25 - 23.13 ) | | | 2.4 ( 0.62 ) | | 2.05 ( 0.31 ) | | 1.04 ( -1.38 ) |
| Cardiac disorder | 1 (0.1%) | | | 2.4 ( 0.25 - 23.13 ) | | | 2.4 ( 0.62 ) | | 2.05 ( 0.31 ) | | 1.04 ( -1.38 ) |
| Electrocardiogram abnormal | 1 (0.1%) | | | 0.9 ( 0.11 - 7.21 ) | | | 0.9 ( 0.01 ) | | 0.91 ( 0.16 ) | | -0.13 ( -2.37 ) |
| Monoplegia | 1 (0.1%) | | | 2.4 ( 0.25 - 23.13 ) | | | 2.4 ( 0.62 ) | | 2.05 ( 0.31 ) | | 1.04 ( -1.38 ) |
| Joint effusion | 1 (0.1%) | | | 7.22 ( 0.45 - 115.42 ) | | | 7.21 ( 2.68 ) | | 4.11 ( 0.4 ) | | 2.04 ( -0.6 ) |
| Eczema | 1 (0.1%) | | | 7.22 ( 0.45 - 115.42 ) | | | 7.21 ( 2.68 ) | | 4.11 ( 0.4 ) | | 2.04 ( -0.6 ) |
| Ultrasound doppler abnormal | 1 (0.1%) | | | 3.61 ( 0.33 - 39.81 ) | | | 3.61 ( 1.26 ) | | 2.74 ( 0.37 ) | | 1.45 ( -1.05 ) |
| Rash macular | 1 (0.1%) | | | 0.6 ( 0.08 - 4.62 ) | | | 0.6 ( 0.24 ) | | 0.63 ( 0.11 ) | | -0.66 ( -2.84 ) |
| Injection site oedema | 1 (0.1%) | | | 7.22 ( 0.45 - 115.42 ) | | | 7.21 ( 2.68 ) | | 4.11 ( 0.4 ) | | 2.04 ( -0.6 ) |
| Poor quality sleep | 1 (0.1%) | | | 1.8 ( 0.2 - 16.15 ) | | | 1.8 ( 0.29 ) | | 1.64 ( 0.26 ) | | 0.72 ( -1.64 ) |
| Sinus disorder | 1 (0.1%) | | | 2.4 ( 0.25 - 23.13 ) | | | 2.4 ( 0.62 ) | | 2.05 ( 0.31 ) | | 1.04 ( -1.38 ) |
| Abnormal dreams | 1 (0.1%) | | | 2.4 ( 0.25 - 23.13 ) | | | 2.4 ( 0.62 ) | | 2.05 ( 0.31 ) | | 1.04 ( -1.38 ) |
| Therapeutic response unexpected | 1 (0.1%) | | | 2.4 ( 0.25 - 23.13 ) | | | 2.4 ( 0.62 ) | | 2.05 ( 0.31 ) | | 1.04 ( -1.38 ) |
| Viral infection | 1 (0.1%) | | | 7.22 ( 0.45 - 115.42 ) | | | 7.21 ( 2.68 ) | | 4.11 ( 0.4 ) | | 2.04 ( -0.6 ) |
| Underdose | 1 (0.1%) | | | 0.26 ( 0.03 - 1.89 ) | | | 0.26 ( 2.07 ) | | 0.28 ( 0.05 ) | | -1.82 ( -3.93 ) |
| Skin texture abnormal | 1 (0.1%) | | | 7.22 ( 0.45 - 115.42 ) | | | 7.21 ( 2.68 ) | | 4.11 ( 0.4 ) | | 2.04 ( -0.6 ) |
| Antibody test positive | 1 (0.1%) | | | 7.22 ( 0.45 - 115.42 ) | | | 7.21 ( 2.68 ) | | 4.11 ( 0.4 ) | | 2.04 ( -0.6 ) |
| Red blood cell sedimentation rate normal | 1 (0.1%) | | | 7.22 ( 0.45 - 115.42 ) | | | 7.21 ( 2.68 ) | | 4.11 ( 0.4 ) | | 2.04 ( -0.6 ) |
| Rotator cuff syndrome | 1 (0.1%) | | | 1.8 ( 0.2 - 16.15 ) | | | 1.8 ( 0.29 ) | | 1.64 ( 0.26 ) | | 0.72 ( -1.64 ) |
| Tendon disorder | 1 (0.1%) | | | 1.8 ( 0.2 - 16.15 ) | | | 1.8 ( 0.29 ) | | 1.64 ( 0.26 ) | | 0.72 ( -1.64 ) |
| Ultrasound joint | 1 (0.1%) | | | 7.22 ( 0.45 - 115.42 ) | | | 7.21 ( 2.68 ) | | 4.11 ( 0.4 ) | | 2.04 ( -0.6 ) |
| Hypopnoea | 1 (0.1%) | | | 7.22 ( 0.45 - 115.42 ) | | | 7.21 ( 2.68 ) | | 4.11 ( 0.4 ) | | 2.04 ( -0.6 ) |
| Lung disorder | 1 (0.1%) | | | 7.22 ( 0.45 - 115.42 ) | | | 7.21 ( 2.68 ) | | 4.11 ( 0.4 ) | | 2.04 ( -0.6 ) |
| Hand deformity | 1 (0.1%) | | | 7.22 ( 0.45 - 115.42 ) | | | 7.21 ( 2.68 ) | | 4.11 ( 0.4 ) | | 2.04 ( -0.6 ) |
| Rheumatoid arthritis | 1 (0.1%) | | | 1.2 ( 0.14 - 9.99 ) | | | 1.2 ( 0.03 ) | | 1.17 ( 0.2 ) | | 0.23 ( -2.05 ) |
| Haemorrhage subcutaneous | 1 (0.1%) | | | 7.22 ( 0.45 - 115.42 ) | | | 7.21 ( 2.68 ) | | 4.11 ( 0.4 ) | | 2.04 ( -0.6 ) |
| Administration site pain | 1 (0.1%) | | | 7.22 ( 0.45 - 115.42 ) | | | 7.21 ( 2.68 ) | | 4.11 ( 0.4 ) | | 2.04 ( -0.6 ) |
| Haematoma | 1 (0.1%) | | | 1.44 ( 0.17 - 12.36 ) | | | 1.44 ( 0.11 ) | | 1.37 ( 0.23 ) | | 0.45 ( -1.86 ) |
| Ageusia | 1 (0.1%) | | | 0.51 ( 0.07 - 3.92 ) | | | 0.52 ( 0.43 ) | | 0.55 ( 0.1 ) | | -0.87 ( -3.03 ) |
| Giant cell arteritis | 1 (0.1%) | | | 3.61 ( 0.33 - 39.81 ) | | | 3.61 ( 1.26 ) | | 2.74 ( 0.37 ) | | 1.45 ( -1.05 ) |
| Red blood cell sedimentation rate increased | 1 (0.1%) | | | 3.61 ( 0.33 - 39.81 ) | | | 3.61 ( 1.26 ) | | 2.74 ( 0.37 ) | | 1.45 ( -1.05 ) |
| Cardiac monitoring | 1 (0.1%) | | | 1.8 ( 0.2 - 16.15 ) | | | 1.8 ( 0.29 ) | | 1.64 ( 0.26 ) | | 0.72 ( -1.64 ) |
| Echocardiogram | 1 (0.1%) | | | 0.66 ( 0.08 - 5.08 ) | | | 0.66 ( 0.17 ) | | 0.68 ( 0.12 ) | | -0.55 ( -2.74 ) |
| Upper-airway cough syndrome | 1 (0.1%) | | | 1.8 ( 0.2 - 16.15 ) | | | 1.8 ( 0.29 ) | | 1.64 ( 0.26 ) | | 0.72 ( -1.64 ) |
| Musculoskeletal discomfort | 1 (0.1%) | | | 1.44 ( 0.17 - 12.36 ) | | | 1.44 ( 0.11 ) | | 1.37 ( 0.23 ) | | 0.45 ( -1.86 ) |
| Guillain-barre syndrome | 1 (0.1%) | | | 1.2 ( 0.14 - 9.99 ) | | | 1.2 ( 0.03 ) | | 1.17 ( 0.2 ) | | 0.23 ( -2.05 ) |
| Coordination abnormal | 1 (0.1%) | | | 7.22 ( 0.45 - 115.42 ) | | | 7.21 ( 2.68 ) | | 4.11 ( 0.4 ) | | 2.04 ( -0.6 ) |
| Sensory disturbance | 1 (0.1%) | | | 1.44 ( 0.17 - 12.36 ) | | | 1.44 ( 0.11 ) | | 1.37 ( 0.23 ) | | 0.45 ( -1.86 ) |
| Muscle contractions involuntary | 1 (0.1%) | | | 7.22 ( 0.45 - 115.42 ) | | | 7.21 ( 2.68 ) | | 4.11 ( 0.4 ) | | 2.04 ( -0.6 ) |
| Abdominal distension | 1 (0.1%) | | | 2.4 ( 0.25 - 23.13 ) | | | 2.4 ( 0.62 ) | | 2.05 ( 0.31 ) | | 1.04 ( -1.38 ) |
| Photopsia | 1 (0.1%) | | | 1.2 ( 0.14 - 9.99 ) | | | 1.2 ( 0.03 ) | | 1.17 ( 0.2 ) | | 0.23 ( -2.05 ) |
| Unresponsive to stimuli | 1 (0.1%) | | | 0.66 ( 0.08 - 5.08 ) | | | 0.66 ( 0.17 ) | | 0.68 ( 0.12 ) | | -0.55 ( -2.74 ) |
| Injection site urticaria | 1 (0.1%) | | | 1.2 ( 0.14 - 9.99 ) | | | 1.2 ( 0.03 ) | | 1.17 ( 0.2 ) | | 0.23 ( -2.05 ) |
| Groin pain | 1 (0.1%) | | | 3.61 ( 0.33 - 39.81 ) | | | 3.61 ( 1.26 ) | | 2.74 ( 0.37 ) | | 1.45 ( -1.05 ) |
| Drug hypersensitivity | 1 (0.1%) | | | 3.61 ( 0.33 - 39.81 ) | | | 3.61 ( 1.26 ) | | 2.74 ( 0.37 ) | | 1.45 ( -1.05 ) |
| Sneezing | 1 (0.1%) | | | 1.03 ( 0.13 - 8.38 ) | | | 1.03 ( 0 ) | | 1.03 ( 0.18 ) | | 0.04 ( -2.22 ) |
| Ear pain | 1 (0.1%) | | | 0.72 ( 0.09 - 5.64 ) | | | 0.72 ( 0.1 ) | | 0.75 ( 0.13 ) | | -0.42 ( -2.63 ) |
| Herpes zoster | 1 (0.1%) | | | 0.29 ( 0.04 - 2.13 ) | | | 0.29 ( 1.69 ) | | 0.32 ( 0.06 ) | | -1.66 ( -3.78 ) |
| Muscle disorder | 1 (0.1%) | | | 1.8 ( 0.2 - 16.15 ) | | | 1.8 ( 0.29 ) | | 1.64 ( 0.26 ) | | 0.72 ( -1.64 ) |
| Musculoskeletal disorder | 1 (0.1%) | | | 1.2 ( 0.14 - 9.99 ) | | | 1.2 ( 0.03 ) | | 1.17 ( 0.2 ) | | 0.23 ( -2.05 ) |
| Diplegia | 1 (0.1%) | | | 3.61 ( 0.33 - 39.81 ) | | | 3.61 ( 1.26 ) | | 2.74 ( 0.37 ) | | 1.45 ( -1.05 ) |
| Heart rate decreased | 1 (0.1%) | | | 2.4 ( 0.25 - 23.13 ) | | | 2.4 ( 0.62 ) | | 2.05 ( 0.31 ) | | 1.04 ( -1.38 ) |
| Face injury | 1 (0.1%) | | | 7.22 ( 0.45 - 115.42 ) | | | 7.21 ( 2.68 ) | | 4.11 ( 0.4 ) | | 2.04 ( -0.6 ) |
| Head injury | 1 (0.1%) | | | 2.4 ( 0.25 - 23.13 ) | | | 2.4 ( 0.62 ) | | 2.05 ( 0.31 ) | | 1.04 ( -1.38 ) |
| Epistaxis | 1 (0.1%) | | | 1.44 ( 0.17 - 12.36 ) | | | 1.44 ( 0.11 ) | | 1.37 ( 0.23 ) | | 0.45 ( -1.86 ) |
| Fluid retention | 1 (0.1%) | | | 7.22 ( 0.45 - 115.42 ) | | | 7.21 ( 2.68 ) | | 4.11 ( 0.4 ) | | 2.04 ( -0.6 ) |
| Aphonia | 1 (0.1%) | | | 7.22 ( 0.45 - 115.42 ) | | | 7.21 ( 2.68 ) | | 4.11 ( 0.4 ) | | 2.04 ( -0.6 ) |
| Mammogram abnormal | 1 (0.1%) | | | 7.22 ( 0.45 - 115.42 ) | | | 7.21 ( 2.68 ) | | 4.11 ( 0.4 ) | | 2.04 ( -0.6 ) |
| Eye pain | 1 (0.1%) | | | 0.8 ( 0.1 - 6.33 ) | | | 0.8 ( 0.04 ) | | 0.82 ( 0.15 ) | | -0.28 ( -2.5 ) |
| Sinus congestion | 1 (0.1%) | | | 1.8 ( 0.2 - 16.15 ) | | | 1.8 ( 0.29 ) | | 1.64 ( 0.26 ) | | 0.72 ( -1.64 ) |
| Delirium | 1 (0.1%) | | | 1.2 ( 0.14 - 9.99 ) | | | 1.2 ( 0.03 ) | | 1.17 ( 0.2 ) | | 0.23 ( -2.05 ) |
| Dementia | 1 (0.1%) | | | 3.61 ( 0.33 - 39.81 ) | | | 3.61 ( 1.26 ) | | 2.74 ( 0.37 ) | | 1.45 ( -1.05 ) |
| Alopecia | 1 (0.1%) | | | 1.8 ( 0.2 - 16.15 ) | | | 1.8 ( 0.29 ) | | 1.64 ( 0.26 ) | | 0.72 ( -1.64 ) |
| Abdominal pain | 1 (0.1%) | | | 0.45 ( 0.06 - 3.4 ) | | | 0.45 ( 0.63 ) | | 0.48 ( 0.09 ) | | -1.05 ( -3.2 ) |
| Covid-19 | 1 (0.1%) | | | 0.01 ( 0 - 0.08 ) | | | 0.01 ( 86.25 ) | | 0.01 ( 0 ) | | -6.22 ( -8.27 ) |
| Respiratory disorder | 1 (0.1%) | | | 3.61 ( 0.33 - 39.81 ) | | | 3.61 ( 1.26 ) | | 2.74 ( 0.37 ) | | 1.45 ( -1.05 ) |
| Sars-cov-2 test positive | 1 (0.1%) | | | 0.09 ( 0.01 - 0.63 ) | | | 0.09 ( 9.39 ) | | 0.1 ( 0.02 ) | | -3.34 ( -5.4 ) |
| Lichen planus | 1 (0.1%) | | | 3.61 ( 0.33 - 39.81 ) | | | 3.61 ( 1.26 ) | | 2.74 ( 0.37 ) | | 1.45 ( -1.05 ) |
| **RSV + COVID-19 + Influenza Vaccines** | | | | | | | | | | | |
| **PT** | | **N(%)** | | | **ROR(95%Cl)** | | | **PRR(X^2^)** | | **EBGM(EBGM05)** | **IC(IC025)** |
| Vomiting | | 13 (2.3%) | | | 2.88 ( 1.61 - 5.14 ) | | | 2.84 ( 14.03 ) | | 2.65 ( 1.63 ) | 1.41 ( 0.59 ) |
| Sleep disorder | | 8 (1.4%) | | | 3.15 ( 1.5 - 6.59 ) | | | 3.12 ( 10.29 ) | | 2.88 ( 1.55 ) | 1.53 ( 0.5 ) |
| Injection site rash | | 7 (1.2%) | | | 3.31 ( 1.5 - 7.31 ) | | | 3.28 ( 9.87 ) | | 3.02 ( 1.56 ) | 1.59 ( 0.5 ) |
| Gait inability | | 6 (1.1%) | | | 9.02 ( 3.54 - 22.97 ) | | | 8.94 ( 31.31 ) | | 6.87 ( 3.14 ) | 2.78 ( 1.53 ) |
| Injection site mass | | 4 (0.7%) | | | 5.99 ( 2.01 - 17.87 ) | | | 5.96 ( 13.38 ) | | 5.01 ( 2.01 ) | 2.33 ( 0.89 ) |
| Injection site induration | | 3 (0.5%) | | | 4.24 ( 1.24 - 14.43 ) | | | 4.22 ( 6.33 ) | | 3.76 ( 1.35 ) | 1.91 ( 0.34 ) |
| Induration | | 2 (0.4%) | | | 5.64 ( 1.22 - 26.18 ) | | | 5.63 ( 6.23 ) | | 4.79 ( 1.33 ) | 2.26 ( 0.39 ) |
| Urinary incontinence | | 2 (0.4%) | | | 5.64 ( 1.22 - 26.18 ) | | | 5.63 ( 6.23 ) | | 4.79 ( 1.33 ) | 2.26 ( 0.39 ) |
| Dysphonia | | 2 (0.4%) | | | 4.23 ( 0.94 - 18.95 ) | | | 4.22 ( 4.22 ) | | 3.76 ( 1.07 ) | 1.91 ( 0.08 ) |
| Injection site bruising | | 2 (0.4%) | | | 1.33 ( 0.32 - 5.54 ) | | | 1.33 ( 0.16 ) | | 1.32 ( 0.4 ) | 0.4 ( -1.33 ) |
| Lethargy | | 2 (0.4%) | | | 1.27 ( 0.31 - 5.26 ) | | | 1.27 ( 0.11 ) | | 1.25 ( 0.38 ) | 0.33 ( -1.4 ) |
| Contusion | | 2 (0.4%) | | | 0.97 ( 0.24 - 4.01 ) | | | 0.97 ( 0 ) | | 0.97 ( 0.3 ) | -0.04 ( -1.75 ) |
| Injection site haemorrhage | | 2 (0.4%) | | | 5.08 ( 1.11 - 23.23 ) | | | 5.06 ( 5.44 ) | | 4.39 ( 1.23 ) | 2.13 ( 0.28 ) |
| Asthma | | 2 (0.4%) | | | 10.16 ( 1.97 - 52.48 ) | | | 10.13 ( 11.76 ) | | 7.52 ( 1.9 ) | 2.91 ( 0.95 ) |
| Productive cough | | 2 (0.4%) | | | 3.63 ( 0.82 - 15.99 ) | | | 3.62 ( 3.32 ) | | 3.29 ( 0.95 ) | 1.72 ( -0.09 ) |
| Retching | | 2 (0.4%) | | | 12.7 ( 2.32 - 69.49 ) | | | 12.66 ( 14.32 ) | | 8.77 ( 2.12 ) | 3.13 ( 1.14 ) |
| Tinnitus | | 2 (0.4%) | | | 1.95 ( 0.46 - 8.24 ) | | | 1.95 ( 0.86 ) | | 1.88 ( 0.56 ) | 0.91 ( -0.84 ) |
| Rash pruritic | | 2 (0.4%) | | | 0.96 ( 0.23 - 3.93 ) | | | 0.96 ( 0 ) | | 0.96 ( 0.29 ) | -0.06 ( -1.78 ) |
| Skin warm | | 2 (0.4%) | | | 0.6 ( 0.15 - 2.45 ) | | | 0.6 ( 0.51 ) | | 0.61 ( 0.19 ) | -0.71 ( -2.41 ) |
| Paraesthesia | | 2 (0.4%) | | | 0.94 ( 0.23 - 3.86 ) | | | 0.94 ( 0.01 ) | | 0.94 ( 0.29 ) | -0.09 ( -1.8 ) |
| Transient ischaemic attack | | 2 (0.4%) | | | 50.82 ( 4.6 - 561.25 ) | | | 50.64 ( 32.45 ) | | 17.55 ( 2.35 ) | 4.13 ( 1.93 ) |
| Neuralgia | | 2 (0.4%) | | | 5.08 ( 1.11 - 23.23 ) | | | 5.06 ( 5.44 ) | | 4.39 ( 1.23 ) | 2.13 ( 0.28 ) |
| Memory impairment | | 2 (0.4%) | | | 6.35 ( 1.35 - 29.97 ) | | | 6.33 ( 7.19 ) | | 5.26 ( 1.44 ) | 2.4 ( 0.51 ) |
| Hypoaesthesia | | 2 (0.4%) | | | 1.08 ( 0.26 - 4.45 ) | | | 1.08 ( 0.01 ) | | 1.07 ( 0.33 ) | 0.1 ( -1.62 ) |
| Feeding disorder | | 2 (0.4%) | | | 5.08 ( 1.11 - 23.23 ) | | | 5.06 ( 5.44 ) | | 4.39 ( 1.23 ) | 2.13 ( 0.28 ) |
| Vertigo | | 2 (0.4%) | | | 1.33 ( 0.32 - 5.54 ) | | | 1.33 ( 0.16 ) | | 1.32 ( 0.4 ) | 0.4 ( -1.33 ) |
| Hyperventilation | | 1 (0.2%) | | | 25.36 ( 1.58 - 406.03 ) | | | 25.32 ( 11.68 ) | | 13.16 ( 1.29 ) | 3.72 ( 1.08 ) |
| Fear | | 1 (0.2%) | | | 3.17 ( 0.4 - 25.38 ) | | | 3.17 ( 1.32 ) | | 2.92 ( 0.51 ) | 1.55 ( -0.69 ) |
| Electric shock sensation | | 1 (0.2%) | | | 8.45 ( 0.88 - 81.39 ) | | | 8.44 ( 4.92 ) | | 6.58 ( 0.99 ) | 2.72 ( 0.3 ) |
| Joint range of motion decreased | | 1 (0.2%) | | | 4.23 ( 0.51 - 35.16 ) | | | 4.22 ( 2.11 ) | | 3.76 ( 0.64 ) | 1.91 ( -0.37 ) |
| Renal pain | | 1 (0.2%) | | | 5.07 ( 0.59 - 43.48 ) | | | 5.06 ( 2.72 ) | | 4.39 ( 0.73 ) | 2.13 ( -0.18 ) |
| Spinal pain | | 1 (0.2%) | | | 8.45 ( 0.88 - 81.39 ) | | | 8.44 ( 4.92 ) | | 6.58 ( 0.99 ) | 2.72 ( 0.3 ) |
| Palpitations | | 1 (0.2%) | | | 0.55 ( 0.08 - 3.99 ) | | | 0.55 ( 0.36 ) | | 0.56 ( 0.11 ) | -0.84 ( -2.92 ) |
| Presyncope | | 1 (0.2%) | | | 2.3 ( 0.3 - 17.88 ) | | | 2.3 ( 0.68 ) | | 2.19 ( 0.39 ) | 1.13 ( -1.06 ) |
| Abdominal pain upper | | 1 (0.2%) | | | 1.01 ( 0.14 - 7.49 ) | | | 1.01 ( 0 ) | | 1.01 ( 0.19 ) | 0.02 ( -2.1 ) |
| Ocular hyperaemia | | 1 (0.2%) | | | 5.07 ( 0.59 - 43.48 ) | | | 5.06 ( 2.72 ) | | 4.39 ( 0.73 ) | 2.13 ( -0.18 ) |
| Swelling face | | 1 (0.2%) | | | 1.06 ( 0.14 - 7.81 ) | | | 1.06 ( 0 ) | | 1.05 ( 0.2 ) | 0.07 ( -2.05 ) |
| Bone pain | | 1 (0.2%) | | | 2.53 ( 0.32 - 19.84 ) | | | 2.53 ( 0.84 ) | | 2.39 ( 0.43 ) | 1.26 ( -0.95 ) |
| Throat irritation | | 1 (0.2%) | | | 2.82 ( 0.36 - 22.27 ) | | | 2.81 ( 1.05 ) | | 2.63 ( 0.47 ) | 1.4 ( -0.82 ) |
| Breast swelling | | 1 (0.2%) | | | 25.36 ( 1.58 - 406.03 ) | | | 25.32 ( 11.68 ) | | 13.16 ( 1.29 ) | 3.72 ( 1.08 ) |
| Pollakiuria | | 1 (0.2%) | | | 3.17 ( 0.4 - 25.38 ) | | | 3.17 ( 1.32 ) | | 2.92 ( 0.51 ) | 1.55 ( -0.69 ) |
| Feeling hot | | 1 (0.2%) | | | 0.6 ( 0.08 - 4.38 ) | | | 0.6 ( 0.26 ) | | 0.61 ( 0.12 ) | -0.71 ( -2.8 ) |
| Musculoskeletal stiffness | | 1 (0.2%) | | | 0.55 ( 0.08 - 3.99 ) | | | 0.55 ( 0.36 ) | | 0.56 ( 0.11 ) | -0.84 ( -2.92 ) |
| Hypersomnia | | 1 (0.2%) | | | 1.33 ( 0.18 - 9.98 ) | | | 1.33 ( 0.08 ) | | 1.32 ( 0.24 ) | 0.4 ( -1.74 ) |
| Feeling cold | | 1 (0.2%) | | | 0.82 ( 0.11 - 5.99 ) | | | 0.82 ( 0.04 ) | | 0.82 ( 0.16 ) | -0.28 ( -2.39 ) |
| Body temperature increased | | 1 (0.2%) | | | 2.53 ( 0.32 - 19.84 ) | | | 2.53 ( 0.84 ) | | 2.39 ( 0.43 ) | 1.26 ( -0.95 ) |
| Oedema peripheral | | 1 (0.2%) | | | 2.11 ( 0.27 - 16.27 ) | | | 2.11 ( 0.54 ) | | 2.02 ( 0.37 ) | 1.02 ( -1.17 ) |
| Somnolence | | 1 (0.2%) | | | 1.1 ( 0.15 - 8.17 ) | | | 1.1 ( 0.01 ) | | 1.1 ( 0.21 ) | 0.13 ( -1.99 ) |
| Muscle swelling | | 1 (0.2%) | | | 8.45 ( 0.88 - 81.39 ) | | | 8.44 ( 4.92 ) | | 6.58 ( 0.99 ) | 2.72 ( 0.3 ) |
| Chronic obstructive pulmonary disease | | 1 (0.2%) | | | 12.68 ( 1.15 - 140.06 ) | | | 12.66 ( 7.16 ) | | 8.77 ( 1.18 ) | 3.13 ( 0.63 ) |
| Ultrasound doppler abnormal | | 1 (0.2%) | | | 12.68 ( 1.15 - 140.06 ) | | | 12.66 ( 7.16 ) | | 8.77 ( 1.18 ) | 3.13 ( 0.63 ) |
| Respiratory tract congestion | | 1 (0.2%) | | | 0.74 ( 0.1 - 5.45 ) | | | 0.74 ( 0.09 ) | | 0.75 ( 0.14 ) | -0.41 ( -2.51 ) |
| Blood glucose increased | | 1 (0.2%) | | | 3.17 ( 0.4 - 25.38 ) | | | 3.17 ( 1.32 ) | | 2.92 ( 0.51 ) | 1.55 ( -0.69 ) |
| Heart rate increased | | 1 (0.2%) | | | 0.74 ( 0.1 - 5.45 ) | | | 0.74 ( 0.09 ) | | 0.75 ( 0.14 ) | -0.41 ( -2.51 ) |
| Viral infection | | 1 (0.2%) | | | 25.36 ( 1.58 - 406.03 ) | | | 25.32 ( 11.68 ) | | 13.16 ( 1.29 ) | 3.72 ( 1.08 ) |
| Nasopharyngitis | | 1 (0.2%) | | | 0.82 ( 0.11 - 5.99 ) | | | 0.82 ( 0.04 ) | | 0.82 ( 0.16 ) | -0.28 ( -2.39 ) |
| Discomfort | | 1 (0.2%) | | | 1.1 ( 0.15 - 8.17 ) | | | 1.1 ( 0.01 ) | | 1.1 ( 0.21 ) | 0.13 ( -1.99 ) |
| Skin texture abnormal | | 1 (0.2%) | | | 25.36 ( 1.58 - 406.03 ) | | | 25.32 ( 11.68 ) | | 13.16 ( 1.29 ) | 3.72 ( 1.08 ) |
| Rotator cuff syndrome | | 1 (0.2%) | | | 6.34 ( 0.71 - 56.81 ) | | | 6.33 ( 3.59 ) | | 5.26 ( 0.84 ) | 2.4 ( 0.04 ) |
| Musculoskeletal pain | | 1 (0.2%) | | | 3.62 ( 0.44 - 29.49 ) | | | 3.62 ( 1.66 ) | | 3.29 ( 0.57 ) | 1.72 ( -0.54 ) |
| Tendon disorder | | 1 (0.2%) | | | 6.34 ( 0.71 - 56.81 ) | | | 6.33 ( 3.59 ) | | 5.26 ( 0.84 ) | 2.4 ( 0.04 ) |
| Immunisation reaction | | 1 (0.2%) | | | 1.69 ( 0.22 - 12.81 ) | | | 1.69 ( 0.26 ) | | 1.65 ( 0.3 ) | 0.72 ( -1.44 ) |
| Ultrasound joint | | 1 (0.2%) | | | 25.36 ( 1.58 - 406.03 ) | | | 25.32 ( 11.68 ) | | 13.16 ( 1.29 ) | 3.72 ( 1.08 ) |
| Ultrasound joint abnormal | | 1 (0.2%) | | | 25.36 ( 1.58 - 406.03 ) | | | 25.32 ( 11.68 ) | | 13.16 ( 1.29 ) | 3.72 ( 1.08 ) |
| Confusional state | | 1 (0.2%) | | | 0.74 ( 0.1 - 5.45 ) | | | 0.74 ( 0.09 ) | | 0.75 ( 0.14 ) | -0.41 ( -2.51 ) |
| Hypopnoea | | 1 (0.2%) | | | 25.36 ( 1.58 - 406.03 ) | | | 25.32 ( 11.68 ) | | 13.16 ( 1.29 ) | 3.72 ( 1.08 ) |
| Nasal congestion | | 1 (0.2%) | | | 1.15 ( 0.15 - 8.56 ) | | | 1.15 ( 0.02 ) | | 1.14 ( 0.21 ) | 0.19 ( -1.93 ) |
| Lung disorder | | 1 (0.2%) | | | 25.36 ( 1.58 - 406.03 ) | | | 25.32 ( 11.68 ) | | 13.16 ( 1.29 ) | 3.72 ( 1.08 ) |
| Neck pain | | 1 (0.2%) | | | 0.57 ( 0.08 - 4.18 ) | | | 0.58 ( 0.31 ) | | 0.58 ( 0.11 ) | -0.77 ( -2.86 ) |
| Ageusia | | 1 (0.2%) | | | 1.81 ( 0.24 - 13.79 ) | | | 1.81 ( 0.34 ) | | 1.75 ( 0.32 ) | 0.81 ( -1.36 ) |
| C-reactive protein increased | | 1 (0.2%) | | | 5.07 ( 0.59 - 43.48 ) | | | 5.06 ( 2.72 ) | | 4.39 ( 0.73 ) | 2.13 ( -0.18 ) |
| Giant cell arteritis | | 1 (0.2%) | | | 12.68 ( 1.15 - 140.06 ) | | | 12.66 ( 7.16 ) | | 8.77 ( 1.18 ) | 3.13 ( 0.63 ) |
| Inflammatory marker increased | | 1 (0.2%) | | | 8.45 ( 0.88 - 81.39 ) | | | 8.44 ( 4.92 ) | | 6.58 ( 0.99 ) | 2.72 ( 0.3 ) |
| Polymyalgia rheumatica | | 1 (0.2%) | | | 3.62 ( 0.44 - 29.49 ) | | | 3.62 ( 1.66 ) | | 3.29 ( 0.57 ) | 1.72 ( -0.54 ) |
| Red blood cell sedimentation rate increased | | 1 (0.2%) | | | 12.68 ( 1.15 - 140.06 ) | | | 12.66 ( 7.16 ) | | 8.77 ( 1.18 ) | 3.13 ( 0.63 ) |
| Injection site reaction | | 1 (0.2%) | | | 0.48 ( 0.07 - 3.45 ) | | | 0.48 ( 0.56 ) | | 0.49 ( 0.09 ) | -1.04 ( -3.12 ) |
| Guillain-barre syndrome | | 1 (0.2%) | | | 4.23 ( 0.51 - 35.16 ) | | | 4.22 ( 2.11 ) | | 3.76 ( 0.64 ) | 1.91 ( -0.37 ) |
| Coordination abnormal | | 1 (0.2%) | | | 25.36 ( 1.58 - 406.03 ) | | | 25.32 ( 11.68 ) | | 13.16 ( 1.29 ) | 3.72 ( 1.08 ) |
| Unresponsive to stimuli | | 1 (0.2%) | | | 2.3 ( 0.3 - 17.88 ) | | | 2.3 ( 0.68 ) | | 2.19 ( 0.39 ) | 1.13 ( -1.06 ) |
| Injection site urticaria | | 1 (0.2%) | | | 4.23 ( 0.51 - 35.16 ) | | | 4.22 ( 2.11 ) | | 3.76 ( 0.64 ) | 1.91 ( -0.37 ) |
| Drug hypersensitivity | | 1 (0.2%) | | | 12.68 ( 1.15 - 140.06 ) | | | 12.66 ( 7.16 ) | | 8.77 ( 1.18 ) | 3.13 ( 0.63 ) |
| Herpes zoster | | 1 (0.2%) | | | 1.01 ( 0.14 - 7.49 ) | | | 1.01 ( 0 ) | | 1.01 ( 0.19 ) | 0.02 ( -2.1 ) |
| Injection site paraesthesia | | 1 (0.2%) | | | 12.68 ( 1.15 - 140.06 ) | | | 12.66 ( 7.16 ) | | 8.77 ( 1.18 ) | 3.13 ( 0.63 ) |
| Diplegia | | 1 (0.2%) | | | 12.68 ( 1.15 - 140.06 ) | | | 12.66 ( 7.16 ) | | 8.77 ( 1.18 ) | 3.13 ( 0.63 ) |
| Face injury | | 1 (0.2%) | | | 25.36 ( 1.58 - 406.03 ) | | | 25.32 ( 11.68 ) | | 13.16 ( 1.29 ) | 3.72 ( 1.08 ) |
| Brain fog | | 1 (0.2%) | | | 0.9 ( 0.12 - 6.66 ) | | | 0.9 ( 0.01 ) | | 0.91 ( 0.17 ) | -0.14 ( -2.25 ) |
| Burning sensation | | 1 (0.2%) | | | 1.21 ( 0.16 - 8.98 ) | | | 1.21 ( 0.03 ) | | 1.2 ( 0.22 ) | 0.26 ( -1.87 ) |
| Lymphadenopathy | | 1 (0.2%) | | | 1.27 ( 0.17 - 9.45 ) | | | 1.27 ( 0.05 ) | | 1.25 ( 0.23 ) | 0.33 ( -1.81 ) |
| Mammogram abnormal | | 1 (0.2%) | | | 25.36 ( 1.58 - 406.03 ) | | | 25.32 ( 11.68 ) | | 13.16 ( 1.29 ) | 3.72 ( 1.08 ) |
| Eye pain | | 1 (0.2%) | | | 2.82 ( 0.36 - 22.27 ) | | | 2.81 ( 1.05 ) | | 2.63 ( 0.47 ) | 1.4 ( -0.82 ) |
| Delirium | | 1 (0.2%) | | | 4.23 ( 0.51 - 35.16 ) | | | 4.22 ( 2.11 ) | | 3.76 ( 0.64 ) | 1.91 ( -0.37 ) |
| Dementia | | 1 (0.2%) | | | 12.68 ( 1.15 - 140.06 ) | | | 12.66 ( 7.16 ) | | 8.77 ( 1.18 ) | 3.13 ( 0.63 ) |
| Tenderness | | 1 (0.2%) | | | 0.57 ( 0.08 - 4.18 ) | | | 0.58 ( 0.31 ) | | 0.58 ( 0.11 ) | -0.77 ( -2.86 ) |
| Alopecia | | 1 (0.2%) | | | 6.34 ( 0.71 - 56.81 ) | | | 6.33 ( 3.59 ) | | 5.26 ( 0.84 ) | 2.4 ( 0.04 ) |
| Abdominal pain | | 1 (0.2%) | | | 1.58 ( 0.21 - 11.96 ) | | | 1.58 ( 0.2 ) | | 1.55 ( 0.29 ) | 0.63 ( -1.52 ) |
| Covid-19 | | 1 (0.2%) | | | 0.04 ( 0.01 - 0.28 ) | | | 0.04 ( 23.12 ) | | 0.04 ( 0.01 ) | -4.54 ( -6.59 ) |
| Respiratory disorder | | 1 (0.2%) | | | 12.68 ( 1.15 - 140.06 ) | | | 12.66 ( 7.16 ) | | 8.77 ( 1.18 ) | 3.13 ( 0.63 ) |
| Sars-cov-2 test positive | | 1 (0.2%) | | | 0.31 ( 0.04 - 2.21 ) | | | 0.31 ( 1.54 ) | | 0.32 ( 0.06 ) | -1.66 ( -3.72 ) |
| Dry skin | | 1 (0.2%) | | | 3.17 ( 0.4 - 25.38 ) | | | 3.17 ( 1.32 ) | | 2.92 ( 0.51 ) | 1.55 ( -0.69 ) |
| Oral mucosal blistering | | 1 (0.2%) | | | 5.07 ( 0.59 - 43.48 ) | | | 5.06 ( 2.72 ) | | 4.39 ( 0.73 ) | 2.13 ( -0.18 ) |
| Constipation | | 1 (0.2%) | | | 6.34 ( 0.71 - 56.81 ) | | | 6.33 ( 3.59 ) | | 5.26 ( 0.84 ) | 2.4 ( 0.04 ) |
| Lichen planus | | 1 (0.2%) | | | 12.68 ( 1.15 - 140.06 ) | | | 12.66 ( 7.16 ) | | 8.77 ( 1.18 ) | 3.13 ( 0.63 ) |
